# Twenty-eight new species of the spider genus *Merizocera* Fage, 1912 (Araneae, Psilodercidae) from South and Southeast Asia

**DOI:** 10.3897/zookeys.961.53058

**Published:** 2020-08-19

**Authors:** Wan-Jin Chang, Zhiyuan Yao, Shuqiang Li

**Affiliations:** 1 Institute of Zoology, Chinese Academy of Sciences, Beijing 100101, China Chinese Academy of Sciences Beijing China; 2 Southeast Asia Biological Diversity Research Institute, Chinese Academy of Sciences, Yezin, Nay Pyi Taw 05282, Myanmar University of Chinese Academy of Sciences Beijing China; 3 College of Life Science, Shenyang Normal University, Shenyang 110034, Liaoning, China Southeast Asia Biological Diversity Research Institute Yezin Myanmar; 4 University of Chinese Academy of Sciences, Beijing 100049, China Shenyang Normal University Shenyang China

**Keywords:** biodiversity, morphology, Ochyroceratidae, taxonomy, tropics

## Abstract

Previously, the genus *Merizocera* Fage, 1912 comprised only seven species from Indonesia, Malaysia, Sri Lanka, and Thailand. In this study, 28 new species are described from South and Southeast Asia: *M. baoshan* Li, **sp. nov.** (♂♀), *M. betong* Li, **sp. nov.** (♂♀), *M. colombo* Li, **sp. nov.** (♂♀), *M. galle* Li, **sp. nov.** (♂♀), *M. hponkanrazi* Li, **sp. nov.** (♂), *M. kachin* Li, **sp. nov.** (♂♀), *M. kandy* Li, **sp. nov.** (♂♀), *M. mandai* Li, **sp. nov.** (♂♀), *M. krabi* Li, **sp. nov.** (♂♀), *M. kurunegala* Li, **sp. nov.** (♂♀), *M. lincang* Li, **sp. nov.** (♀), *M. mainling* Li, **sp. nov.** (♂♀), *M. nyingchi* Li, **sp. nov.** (♀), *M. peraderiya* Li, **sp. nov.** (♂♀), *M. phuket* Li, **sp. nov.** (♂♀), *M. putao* Li, **sp. nov.** (♂♀), *M. ranong* Li, **sp. nov.** (♂♀), *M. ratnapura* Li, **sp. nov.** (♂♀), *M. salawa* Li, **sp. nov.** (♂), *M. tak* Li, **sp. nov.** (♀), *M. tanintharyi* Li, **sp. nov.** (♂♀), *M. tengchong* Li, **sp. nov.** (♂), *M. thenna* Li, **sp. nov.** (♂♀), *M. uva* Li, **sp. nov.** (♀), *M. wenshan* Li, **sp. nov.** (♂♀), *M. wui* Li, **sp. nov.** (♂♀), *M. yala* Li, **sp. nov.** (♀), and *M. yuxi* Li, **sp. nov.** (♂♀). Among them the genus *Merizocera* is reported for the first time from China, Myanmar, and Singapore.

## Introduction

The spider family Psilodercidae was proposed as Psilodercinae by Machado (1951) under the family Ochyroceratidae Fage, 1912. Deeleman-Reinhold (1995) studied the Indo-Pacific ochyroceratids and erected two subfamilies, Theotiminae and Psilodercinae: Psilodercidae was elevated to family rank by Wunderlich (2008), and Pérez-González et al. (2016) formally confirmed that Psilodercinae Machado, 1951, has priority over Psilodercinae Deeleman-Reinhold, 1995.

The family is restricted to tropical South Asia, southern China, and Southeast Asia (WSC 2020). It currently includes eleven genera and 196 species, of which *Luzonacera*, *Qiongocera*, *Relictocera*, *Sinoderces*, and *Thaiderces*, all authored by Li & Li, 2017, and *Priscaleclercera* Wunderlich, 2017, were described only recently (Liu et al. 2017; Li 2020; WSC 2020). Currently, the genus *Merizocera* contains only seven species: *M. brincki* Brignoli, 1975, *M. cruciata* (Simon, 1893), *M. oryzae* Brignoli, 1975, and *M. picturata* (Simon, 1893) from Sri Lanka; *M. pygmaea* Deeleman-Reinhold, 1995 from Thailand; *M. crinita* (Fage, 1929) from Malaysia; and *M. stellata* (Simon, 1905) from Indonesia (Simon 1893a, b; WSC 2020). Of these seven known species, four were described from only a single male or female specimen.

In this paper, 28 new species of *Merizocera* collected in southern China, Myanmar, Singapore, Sri Lanka, and Thailand are described and illustrated. This is the first record of the genus in China, Myanmar, and Singapore.

## Materials and methods

Types are deposited in the Institute of Zoology, Chinese Academy of Sciences (**IZCAS**) in Beijing. All specimens collected were studied and preserved in 75% ethanol. The specimens were measured and examined with a Leica M205 C stereomicroscope and further morphological details were observed with an Olympus BX41 compound microscope. Male palps were detached from the left side of the animal for further examination. Carapace length was measured excluding the clypeus. Internal genitalia of the female and palpal bulbs were dissected and immersed in lactic acid. An Olympus C7070 wide zoom digital camera (7.1 megapixels) mounted on an Olympus SZX12 stereomicroscope was used to take photos at different focal planes. The photos were then transferred to the image stacking software Helicon Focus 6.7.1 to generate photos with a greater depth of field before further processing with Adobe Photoshop CC 2014. Leg measurements are shown as total length: femur, patella, tibia, metatarsus and tarsus. Leg segments were measured from their retrolateral side. All measurements are given in millimetres (mm). All terminology follows that of Li et al (2014). The distribution map was generated with Google Earth Pro 7.3.2 (Google Limited Liability Company).

## Taxonomy

### Family Psilodercidae Machado, 1951

#### Genus Merizocera

Taxon classificationAnimaliaAraneaePsilodercidae

Fage, 1912

7CEE1EF7-62D8-57B2-A986-88CA8071746B

##### Type species.

*Ochyrocera cruciata*Simon 1893a: 282, fig. 245, from Sri Lanka.

##### Diagnosis.

*Merizocera* can be recognised by the following combination of characters: 1) presence of cymbial protrusion (except *M. mainling* sp. nov. and *M. tanintharyi* sp. nov.); 2) bulb and cymbium almost similar in length or bulb longer than cymbium; 3) absence of clypeal protrusion (except *M. mainling* sp. nov. and *M. putao* sp. nov.); 4) presence or absence of conductor, if present, connected basally with embolus; 5) elongated pyriform bulb with embolus and conductor (if present) arising distally; 6) cheliceral promargin with lamina having three triangular extensions, retromargin with two small teeth.

##### Composition.

*Merizocera cruciata* (♂♀) (the type species), *M. baoshan* sp. nov. (♂♀), *M. betong* sp. nov. (♂♀), *M. brincki* (♂), *M. colombo* sp. nov. (♂♀), *M. crinita* (♂♀), *M. galle* sp. nov. (♂♀), *M. hponkanrazi* sp. nov. (♂), *M. kachin* sp. nov. (♂♀), *M. kandy* sp. nov. (♂♀), *M. mandai* sp. nov. (♂♀), *M. krabi* sp. nov. (♂♀), *M. kurunegala* sp. nov. (♂♀), *M. lincang* sp. nov. (♀), *M. mainling* sp. nov. (♂♀), *M. nyingchi* sp. nov. (♀), *M. oryzae* (♀), *M. peraderiya* sp. nov. (♂♀), *M. phuket* sp. nov. (♂♀), *M. picturata* (♂♀), *M. putao* sp. nov. (♂♀), *M. pygmaea* (♀), *M. ranong* sp. nov. (♂♀), *M. ratnapura* sp. nov. (♂♀), *M. salawa* sp. nov. (♂), *M. stellata* (♀), *M. tak* sp. nov. (♀), *M. tanintharyi* sp. nov. (♂♀), *M. tengchong* sp. nov. (♂), *M. thenna* sp. nov. (♂♀), *M. uva* sp. nov. (♀), *M. wenshan* sp. nov. (♂♀), *M. wui* sp. nov. (♂♀), *M. yala* sp. nov. (♀) and *M. yuxi* sp. nov. (♂♀).

##### Remarks.

Although the genus *Merizocera* cannot be sufficiently delineated by features of their female genitalia, the somatic morphology and male palp structures are consistent with those of *Merizocera* sensu Li & Li, 2018.

##### Distribution.

The genus is represented by species ranging from Sri Lanka to China’s western and southern provinces and to parts of mainland Southeast Asia and beyond, with Java in Indonesia as its currently known southern limit.

#### Merizocera baoshan

Taxon classificationAnimaliaAraneaePsilodercidae

Li sp. nov.

F613E874-E52C-5069-AE21-302A42B2DAAB

http://zoobank.org/6B67BB8B-DDA0-419D-BCD6-675144ECB22E

Figures 1 , 2 , 53

##### Type material.

***Holotype***: male (IZCAS), Luoshui Cave (25°20.35'N, 98°32.28'E, elevation 1937 m), Jiangdong Mountain, Jiangdong Village, Gudong Town, Tengchong County, Baoshan, **Yunnan**, **China**, 15 July 2016, Y. Li leg. ***Paratypes***: 3 males and 3 females (IZCAS), same data as holotype.

##### Etymology.

The specific name refers to the type locality; noun in apposition.

##### Diagnosis.

**Figure 1.**
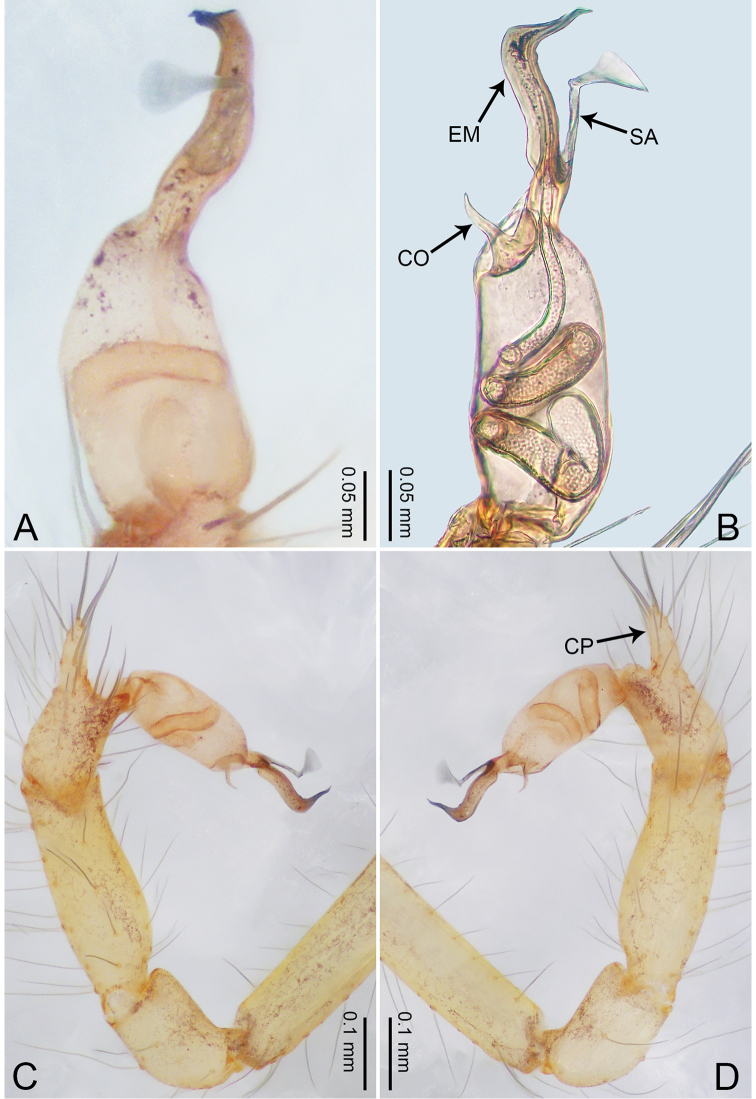
*Merizocera baoshan* sp. nov., holotype male. **A** Bulb, dorsal view **B** bulb, retrolateral view **C** palp, prolateral view **D** palp, retrolateral view. Abbreviations: CO = conductor, CP = cymbial protrusion, EM = embolus, SA = stalked apophysis.

Males of *M. baoshan* sp. nov. resemble those of *M. tengchong* sp. nov., but can be distinguished by the pointed and bent tip of the embolus (Fig. 1B) (vs. blunt and upright tip of embolus (Fig. 40B)); a distinct stalked apophysis bearing a pointed distal tip adjacent to the embolus (Fig. 1B) (vs. stalked apophysis bearing globose distal tip (Fig. 40B)); a cymbial protrusion half the length of the tegular (Fig. 1D) (vs. cymbial protrusion 1/4 the length of tegular (Fig. 40D)). The female can be distinguished from congeners by a pair of flattened ovoid spermathecae.

##### Description.

**Figure 2.**
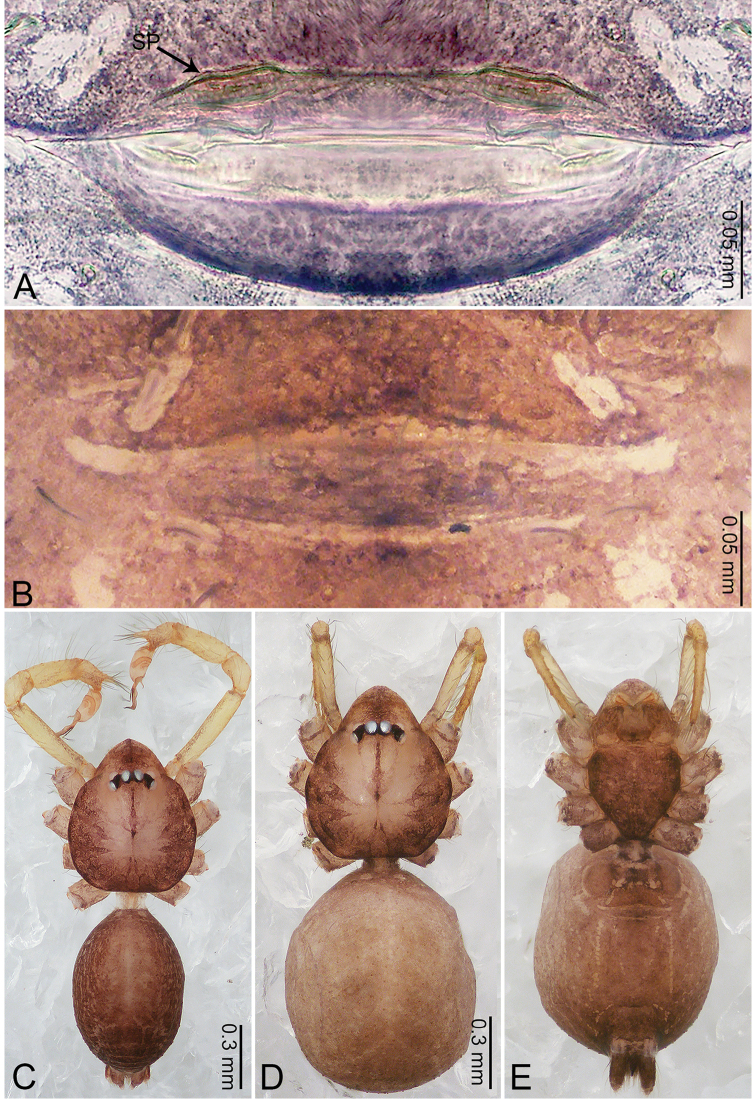
*Merizocera baoshan* sp. nov., holotype male and paratype female. **A** Endogyne, dorsal view **B** female epigastric area, ventral view **C** male habitus, dorsal view **D** female habitus, dorsal view **E** female habitus, ventral view. Abbreviation: SP = spermatheca.

**Male** (holotype). Total length 1.65; carapace 0.75 long, 0.61 wide; abdomen 0.80 long, 0.55 wide. Carapace circular, brownish, with dark brown marks laterally and dark brown median line on anterior half (Fig. 2C). Fovea shallow. Thoracic region distinctly elevated medially. Clypeus, labium and sternum dark brown. Abdomen ovoid, dark brown (Fig. 2C). Legs light brown; measurements: I and II missing, III 4.02 (1.13, 0.22, 1.13, 0.98, 0.56), IV 5.70 (1.48, 0.25, 1.70, 1.44, 0.83). Palp (Fig. 1A–D): femur slender, three times longer than patella; patella not swollen; tibia 2/3 length of femur; cymbium with distal protrusion, 1/3 length of femur, length ratio of dorsal protrusion and cymbium 0.57; bulb light brown, elongated pyriform with embolus, conductor and stalked apophysis emerging distally; embolus similar in length and 1/3 the width of tegular, with darkened pointed tip and bent at right angle; conductor tentacle-like, basally attached with embolus; stalked apophysis basally attached with embolus, bearing triangular pointed distal part.

**Female** (paratype). General features and colouration similar to those of male (Fig. 2D, E). Measurements: total length 1.68; carapace 0.71 long, 0.61 wide; abdomen 0.94 long, 0.80 wide. Leg measurements: I 4.75 (1.24, 0.25, 1.42, 1.14, 0.70), II 3.92 (1.04, 0.25, 1.10, 0.82, 0.61), III 3.27 (0.87, 0.22, 0.88, 0.80, 0.50), IV 4.76 (1.24, 0.24, 1.39, 1.13, 0.76). Epigastric area (Fig. 2B): brown, with a pair of slanting, short, pale brown bands anteriorly, posterior spanned with pale brown horizontal band. Endogyne (Fig. 2A) with pair of connected, flattened, ovoid spermathecae.

##### Distribution.

Known only from the type locality (China; Fig. 53).

#### Merizocera betong

Taxon classificationAnimaliaAraneaePsilodercidae

Li sp. nov.

AF4B8E36-7445-5714-A215-C9C749B380E0

http://zoobank.org/AC63AF3E-1F1B-4A27-BBE4-0AF87A7D831E

Figures 3 , 4 , 54

##### Type material.

***Holotype***: male (IZCAS), Ban Bo Nam Ron Village (5°49.96'N, 101°4.08'E, elevation 384 m), Betong District, **Yala**, **Thailand**, 24 October 2015, P. Wongprom leg. ***Paratypes***: 1 male and 3 females (IZCAS), same data as holotype.

##### Etymology.

The specific name refers to the type locality; noun in apposition.

##### Diagnosis.

**Figure 3.**
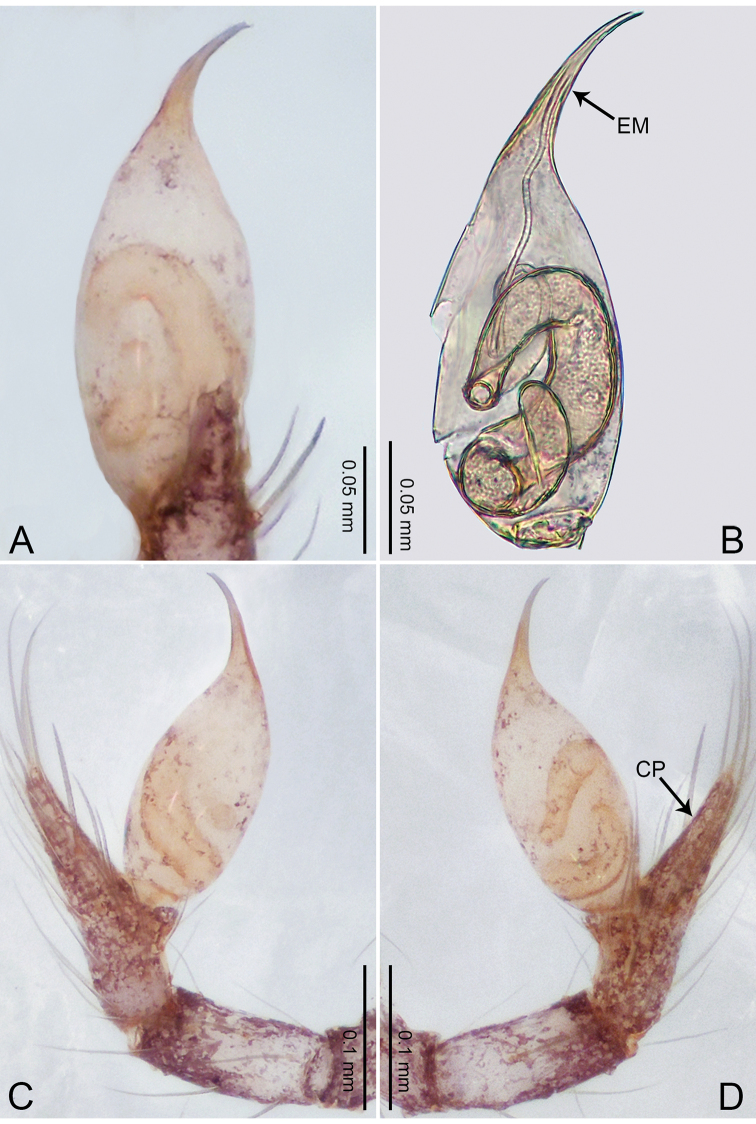
*Merizocera betong* sp. nov., holotype male. **A** Bulb, dorsal view **B** bulb, retrolatero-dorsal view **C** palp, prolateral view **D** palp, retrolateral view. Abbreviations: CP = cymbial protrusion, EM = embolus.

**Figure 4.**
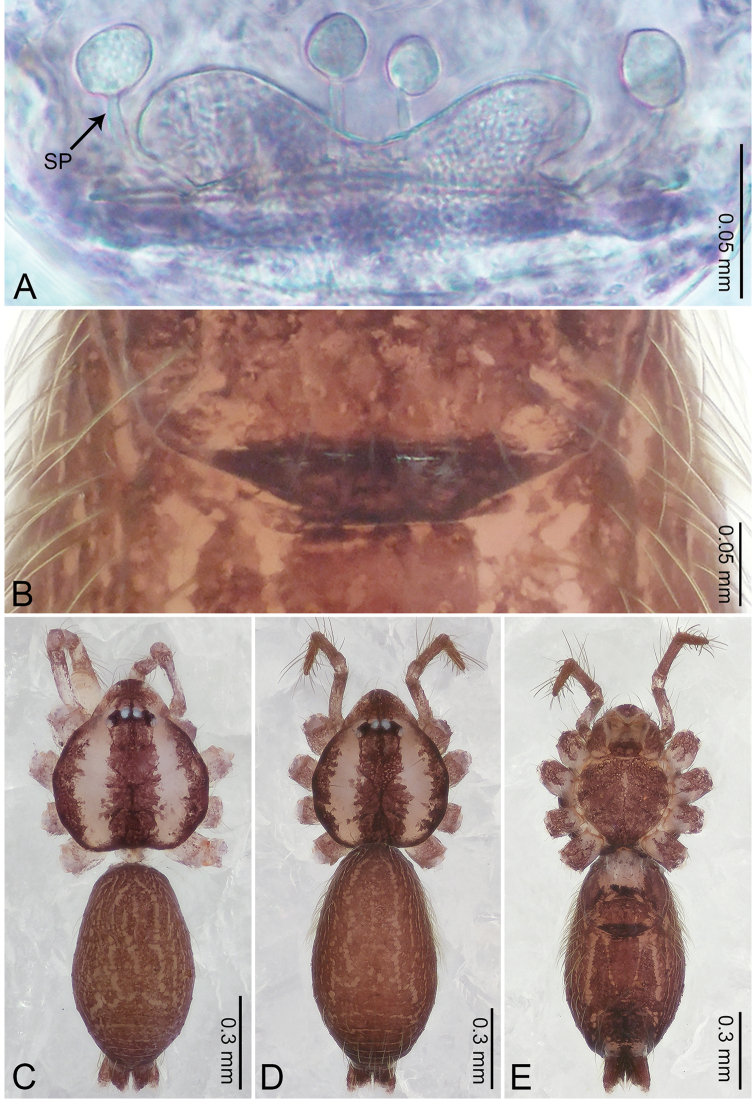
*Merizocera betong* sp. nov., holotype male and paratype female. **A** Endogyne, dorsal view **B** female epigastric area, ventral view **C** male habitus, dorsal view **D** female habitus, dorsal view **E** female habitus, ventral view. Abbreviation: SP = spermatheca.

Males of *M. betong* sp. nov. resemble those of *M. ranong* sp. nov. and *M. yuxi* sp. nov., but can be distinguished by the smooth distally arising embolus (Fig. 3B) (vs. the crinkly distally arising embolus in *M. ranong* sp. nov. (Fig. 31B) and *M. yuxi* sp. nov. (Fig. 50B)), slender pyriform bulb (Fig. 3B) (vs. swollen pyriform bulb in *M. ranong* sp. nov. (Fig. 31B) and *M. yuxi* sp. nov. (Fig. 50B)), pointed embolus tip (Fig. 3B) (vs. lamina-like embolus tip in *M. ranong* sp. nov. (Fig. 31B) and flattened tip in *M. yuxi* sp. nov. (Fig. 50B)), embolus stalk 1/2 the length of tegular (Fig. 3B) (vs. embolus stalk 1/3 length of tegular in *M. ranong* sp. nov. (Fig. 31B), and similar in length in *M. yuxi* sp. nov. (Fig. 50B)), cymbial protrusion half the length of tegular in *M. betong* sp. nov. (Fig. 3D) and *M. ranong* sp. nov. (Fig. 31D) (vs. cymbial protrusion similar length with tegular in *M. yuxi* sp. nov. (Fig. 50D)). These species appear similar to those in the *septentrionalis* group of the genus *Psiloderces*, but can be distinguished by the more distinct cymbial protrusion (longer than the bulb or at least half the bulb’s length) (vs. cymbial protrusion inconspicuous or shorter than half the length of bulb in *Psiloderces*). The female can be distinguished by having two pairs of stalked spermathecae each bearing a globose distal part (Fig. 4A) (vs. one pair of posteriorly directed tubular spermathecae in *M. ranong* sp. nov. (Fig. 32A) and two pairs of tubular spermathecae in *M. yuxi* sp. nov. (Fig. 51A)).

##### Description.

**Male** (holotype). Total length 1.33; carapace 0.56 long, 0.51 wide; abdomen 0.71 long, 0.40 wide. Carapace circular, brownish, with dark brown marks laterally and dark brown median stripe (Fig. 4C). Fovea shallow. Thoracic region distinctly elevated medially. Clypeus, labium, and sternum dark brown. Abdomen slightly elongated, dark brown (Fig. 4E). Legs brown; measurements: I 6.85 (1.84, 0.19, 2.13, 1.86, 0.83), II 5.05 (1.38, 0.19, 1.53, 1.28, 0.67), III 3.79 (1.04, 0.18, 1.11, 0.94, 0.52), IV 5.50 (1.48, 0.18, 1.74, 1.42, 0.68). Palp (Fig. 3A–D): femur slender, three times longer than patella; patella not swollen; tibia half as long as femur; cymbium with distal protrusion, half as long as femur, length ratio of dorsal protrusion and cymbium 1.45; bulb pale yellow, pyriform with embolus arising distally; embolus stalk slightly bent with pointed embolus tip, embolus approx. half the length of tegular.

**Female** (paratype). General features and colouration similar to those of the male (Fig. 4D, E). Measurements: total length 1.52; carapace 0.61 long, 0.51 wide; abdomen 0.92 long, 0.48 wide. Leg measurements: I 5.09 (1.33, 0.17, 1.60, 1.28, 0.71), II 4.54 (1.23, 0.18, 1.34, 1.13, 0.66), III 3.46 (0.93, 0.18, 0.98, 0.86, 0.51), IV 5.11 (1.31, 0.19, 1.62, 1.28, 0.71). Epigastric area (Fig. 4B) with oval dark brown patch. Endogyne (Fig. 4A) with two pairs of stalked spermathecae, globose distally with medially curved receptacle.

##### Distribution.

Known only from the type locality (Thailand; Fig. 54).

#### Merizocera colombo

Taxon classificationAnimaliaAraneaePsilodercidae

Li sp. nov.

BCBD302B-0CC1-5333-BEB4-119DBD56DB33

http://zoobank.org/77AD96FF-8BBD-4474-AF0A-0681EB037B41

Figures 5 , 6 , 52

##### Type material.

***Holotype***: male (IZCAS), Mahawafa Hill (6°55.92'N, 80°14.68'E, elevation 38 m), Mahawafa Village, Avissawella Town, Maniyangama, Colombo District, **Western Province**, **Sri Lanka**, 26 September 2014, S. Kosala leg. ***Paratype***: 1 female (IZCAS), same data as holotype.

##### Etymology.

The specific name refers to the type locality; noun in apposition.

##### Diagnosis.

**Figure 5.**
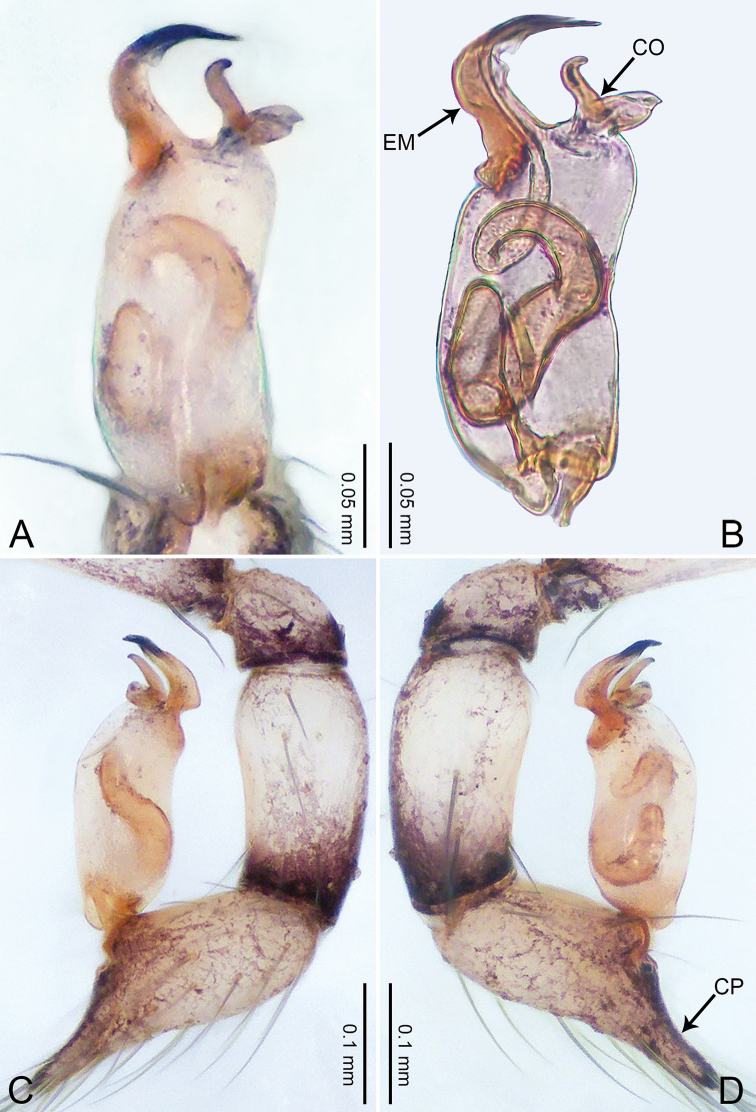
*Merizocera colombo* sp. nov., holotype male. **A** Bulb, dorsal view **B** bulb, dorsal view **C** palp, prolateral view **D** palp, retrolateral view. Abbreviations: CO = conductor, CP = cymbial protrusion, EM = embolus.

**Figure 6.**
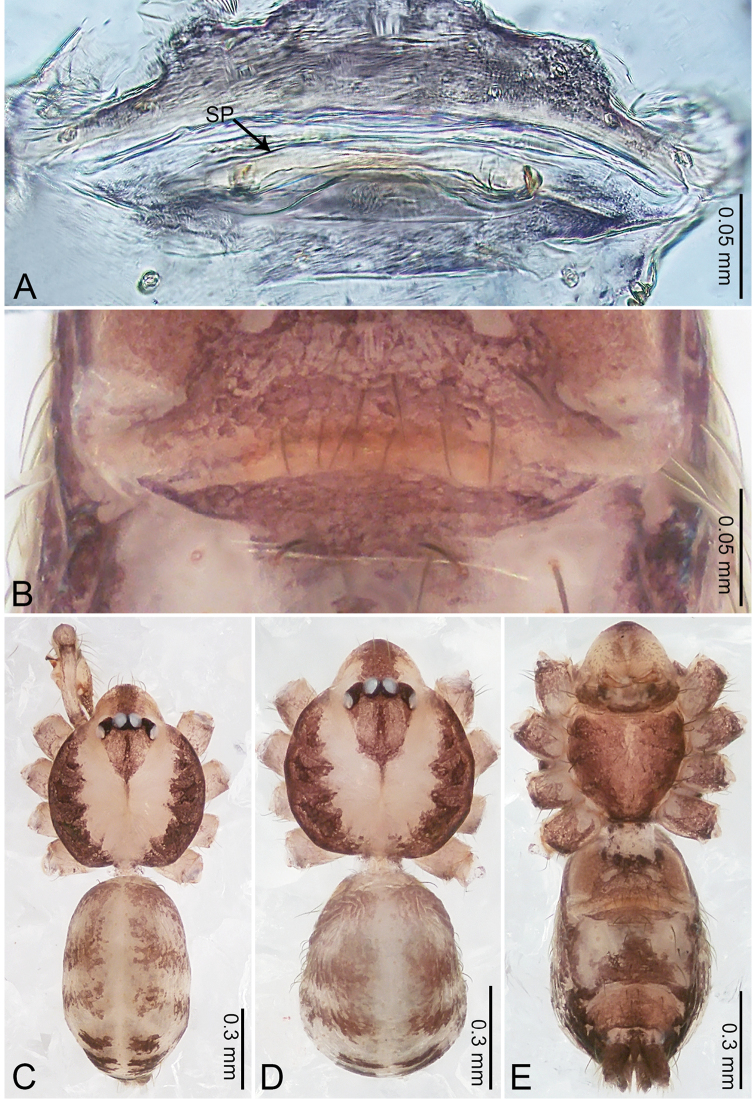
*Merizocera colombo* sp. nov., holotype male and paratype female. **A** Endogyne, dorsal view **B** female epigastric area, ventral view **C** male habitus, dorsal view **D** female habitus, dorsal view **E** female habitus, ventral view. Abbreviation: SP = spermatheca.

Males of *M. colombo* sp. nov. resemble *M. oryzae*, but can be distinguished by a distinct cymbial protrusion approx. 1/2 length of bulb (Fig. 5D) (vs. inconspicuous cymbial protrusion approx. 1/3 the length of bulb), conductor bifurcate and distinctly shorter than embolus (Fid. 5B) (vs. conductor not bifurcate and almost similar length with embolus). Females can be distinguished by having a pair of elongated spermathecae concaving posteriorly (Fig. 6A).

##### Description.

**Male** (holotype). Total length 1.48; carapace 0.69 long, 0.59 wide; abdomen 0.75 long, 0.49 wide. Carapace circular, brownish, with dark brown marks laterally and dark median stripe on anterior half (Fig. 6C). Fovea shallow. Thoracic region distinctly elevated medially. Clypeus brownish, with dark brown marks medially. Labium and sternum dark brown. Abdomen slightly elongated, brownish, with dark brown marks dorsally and ventrally (Fig. 6E). Legs light brown; measurements: I 6.07 (1.62, 0.23, 1.80, 1.60, 0.82), II 4.46 (1.20, 0.21, 1.31, 1.13, 0.61), III 3.67 (1.03, 0.20, 1.03, 0.95, 0.46), IV 5.74 (1.48, 0.21, 1.78, 1.50, 0.77). Palp (Fig. 5A–D): femur four times longer than patella; patella not swollen; tibia 2/3 length of femur; cymbium with distal protrusion, half length of femur, length ratio of dorsal protrusion and cymbium 0.76; bulb light brown, slender pyriform-shaped with embolus and conductor arising distally; embolus hooked with pointed tip; conductor bifurcated, one narrower and with slightly hooked tip, adjacent to embolus.

**Female** (paratype). General features and colouration similar to those of the male (Fig. 6D, E). Measurements: total length 1.28; carapace 0.65 long, 0.56 wide; abdomen 0.59 long, 0.50 wide. Leg measurements: I 4.33 (1.08, 0.21, 1.31, 1.06, 0.67), II 3.36 (0.84, 0.20, 0.93, 0.82, 0.57), III 2.98 (0.78, 0.20, 0.80, 0.75, 0.45), IV 4.39 (1.11, 0.20, 1.31, 1.11, 0.66). Epigastric area (Fig. 6B): dark brown oval patch with light brown slit medially. Endogyne (Fig. 6A) with pair of elongated spermathecae slightly concave toward posterior.

##### Distribution.

Known only from the type locality (Sri Lanka; Fig. 52).

#### Merizocera galle

Taxon classificationAnimaliaAraneaePsilodercidae

Li sp. nov.

F09781D9-7224-5822-A403-972C646D6E44

http://zoobank.org/17373A33-AC34-4331-A192-2F6C85188E39

Figures 7 , 8 , 52

##### Type material.

***Holotype***: male (IZCAS), Rumassala Mountain (6°1.48'N, 80°14.55'E, elevation 51 m), Unawatuna Village, Galle District, **Southern Province**, **Sri Lanka**, 12–13 October 2014, S. Kosala leg. ***Paratype***: 1 female (IZCAS), same data as holotype.

##### Etymology.

The specific name refers to the type locality; noun in apposition.

##### Diagnosis.

**Figure 7.**
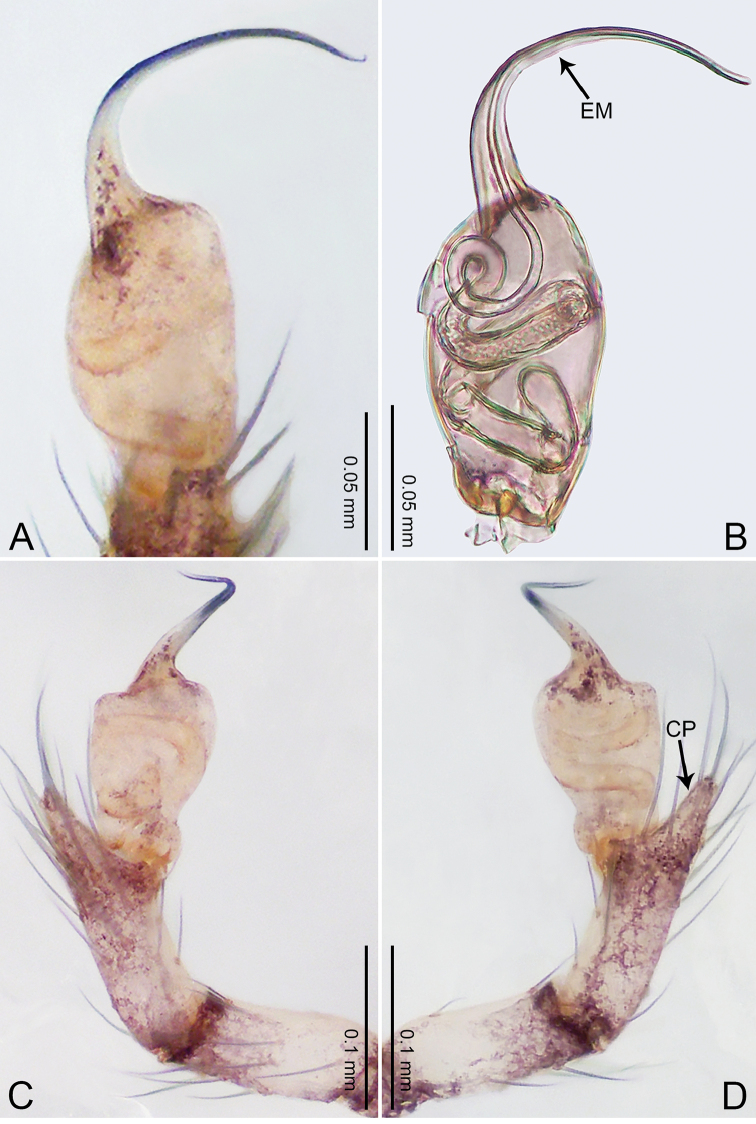
*Merizocera galle* sp. nov., holotype male. **A** Bulb, dorsal view **B** bulb, dorsal view **C** palp, prolateral view **D** palp, retrolateral view. Abbreviations: CP = cymbial protrusion, EM = embolus.

**Figure 8.**
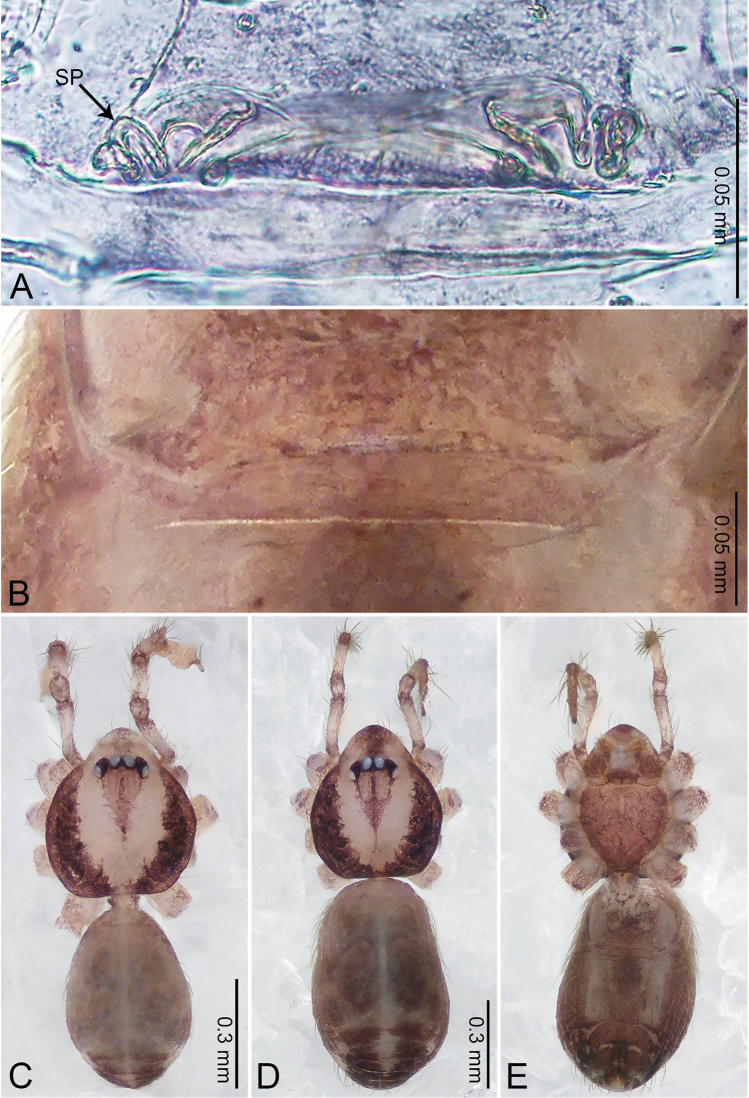
*Merizocera galle* sp. nov., holotype male and paratype female. **A** Endogyne, dorsal view **B** female epigastric area, ventral view **C** male habitus, dorsal view **D** female habitus, dorsal view **E** female habitus, ventral view. Abbreviation: SP = spermatheca.

**Figure 9.**
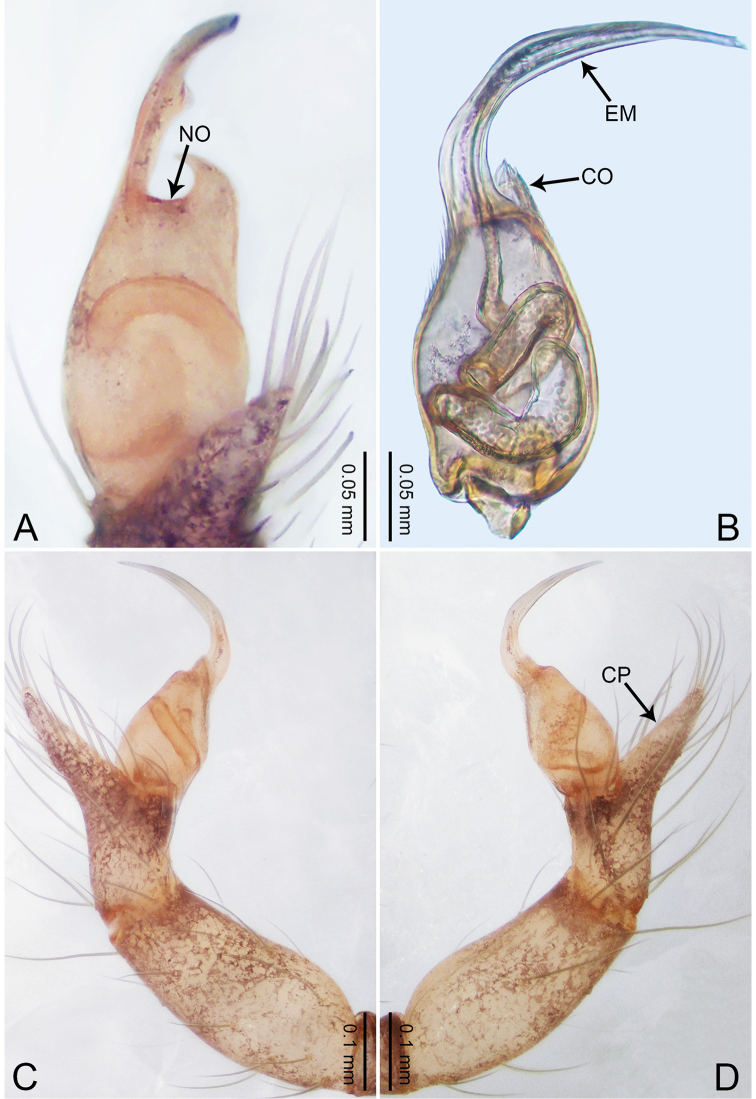
*Merizocera hponkanrazi* sp. nov., holotype male. **A** Bulb, dorsal view **B** bulb, retrolatero-dorsal view **C** palp, prolateral view **D** palp, retrolateral view. Abbreviations: CO = conductor, CP = cymbial protrusion, EM = embolus, NO = notch.

Males can be distinguished from congeners by the distinctly longer (longer than tegular) and bent embolus (Fig. 7B); from *M. ratnapura* sp. nov. (Fig. 33B), *M. phuket* sp. nov. (Fig. 27B), and *M. hponkanrazi* sp. nov. (Fig. 9B) by the absence of conductor (vs. presence of distinct conductor projected from the base of embolus). The females can be distinguished by a pair of horizontally twisted spermathecae (Fig. 8A).

##### Description.

**Male** (holotype). Total length 1.00; carapace 0.48 long, 0.42 wide; abdomen 0.51 long, 0.36 wide. Carapace circular, brownish, with dark brown marks laterally and brown median stripe on anterior half (Fig. 8C). Fovea shallow. Thoracic region distinctly elevated medially. Clypeus brownish, with dark brown marks medially. Labium and sternum dark brown. Abdomen slightly elongated, dark grey, with dark marks posteriorly and ventrally. Legs brown; measurements: I 3.73 (1.00, 0.17, 1.13, 0.90, 0.53), II 2.98 (0.79, 0.16, 0.85, 0.72, 0.46), III 2.49 (0.70, 0.13, 0.67, 0.63, 0.36), IV 3.74 (0.98, 0.16, 1.13, 0.96, 0.51). Palp (Fig. 7A–D): femur slender, thrice longer than patella; patella not swollen; tibia similar length as femur; cymbium with distal protrusion, half length of femur, length ratio of dorsal protrusion and cymbium 0.54; bulb pale yellow, pyriform with embolus arising distally, conductor absent; embolus distinctly elongated and bent, 1.5 times longer than the tegular.

**Female** (paratype). General features and colouration similar to those of male (Fig. 8D, E). Measurements: total length 1.24; carapace 0.53 long, 0.45 wide; abdomen 0.70 long, 0.48 wide. Leg measurements: I 3.62 (0.94, 0.17, 1.08, 0.89, 0.54), II 3.03 (0.78, 0.17, 0.87, 0.73, 0.48), III missing, IV 3.70 (0.95, 0.16, 1.13, 0.92, 0.54). Epigastric area (Fig. 8B): brown, lanceolate patch. Endogyne (Fig. 8A) with pair of horizontally twisted spermathecae, ratio of the width of twisted spermatheca and the interdistance of spermathecae 1:4.

##### Distribution.

Known only from the type locality (Sri Lanka; Fig. 52).

#### Merizocera hponkanrazi

Taxon classificationAnimaliaAraneaePsilodercidae

Li sp. nov.

1CC26426-F344-516B-8F13-45C4399A37AE

http://zoobank.org/6059ACD5-748E-4F8B-B8A0-6EB09F36BFF0

Figures 9 , 10 , 53

##### Type material.

***Holotype***: male (IZCAS), Roadside between Camp 2 and Camp 3 (27°37.15'N, 96°58.92'E, elevation 2806 m), Hponkanrazi Wildlife Sanctuary, Putao, **Kachin State**, **Myanmar**, 16 December 2016, J. Wu leg.

##### Etymology.

The specific name refers to the type locality; noun in apposition.

##### Diagnosis.

Males resemble *M. krabi* sp. nov. but can be distinguished by the embolus longer than the tegular (Fig. 9B) (vs. embolus length half the tegular (Fig. 17B)), presence of notch at tegular tip (Fig. 9A) (vs. absence of notch), bulb shortened pyriform (vs. elongated pyriform bulb).

##### Description.

**Figure 10.**
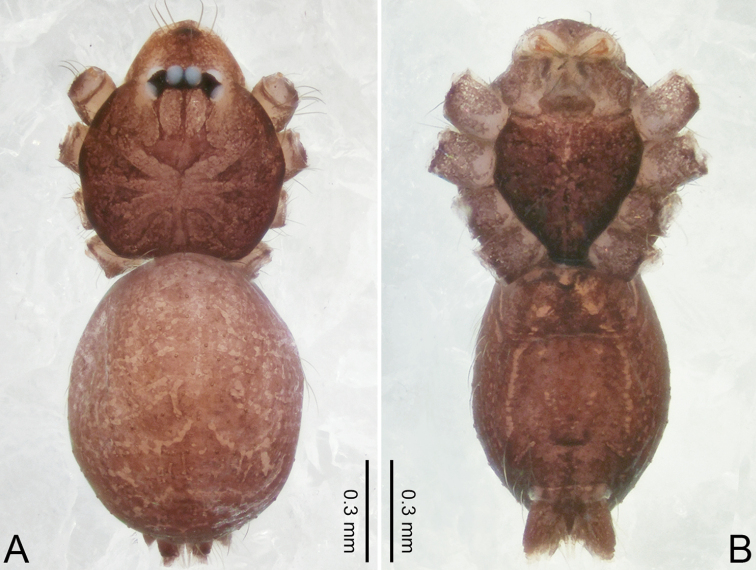
*Merizocera hponkanrazi* sp. nov., holotype male. **A** Habitus, dorsal view **B** habitus, ventral view.

**Male** (holotype). Total length 1.56; carapace 0.71 long, 0.62 wide; abdomen 0.85 long, 0.71 wide. Carapace circular, brown, with dark brown radiating marks (Fig. 10A). Fovea shallow. Thoracic region distinctly elevated medially. Clypeus, labium and sternum dark brown. Abdomen ovoid, dark brown (Fig. 10B). Legs brown; measurements: I 5.43 (1.44, 0.25, 1.66, 1.33, 0.75), II 4.65 (1.28, 0.24, 1.41, 1.11, 0.61), III 3.46 (0.95, 0.20, 0.92, 0.85, 0.54), IV 4.83 (1.25, 0.22, 1.42, 1.21, 0.73). Palp (Fig. 9A–D): tibia swollen proximally; cymbium with distal protrusion, length ratio of dorsal elongation and cymbium 1.13; bulb brown, pyriform, with embolus and conductor arising distally; embolus distinctly bent and 1.2 times longer than tegular; conductor with pointed tip, 1/6 length of embolus, adjacent to a notch.

**Female.** Unknown.

##### Distribution.

Known only from the type locality (Myanmar; Fig. 53).

#### Merizocera kachin

Taxon classificationAnimaliaAraneaePsilodercidae

Li sp. nov.

97018FF1-3BB1-5419-84F3-9D05758CB15B

http://zoobank.org/17A235EF-2414-43F7-97AC-5AD66FC8314A

**Figure 11.**
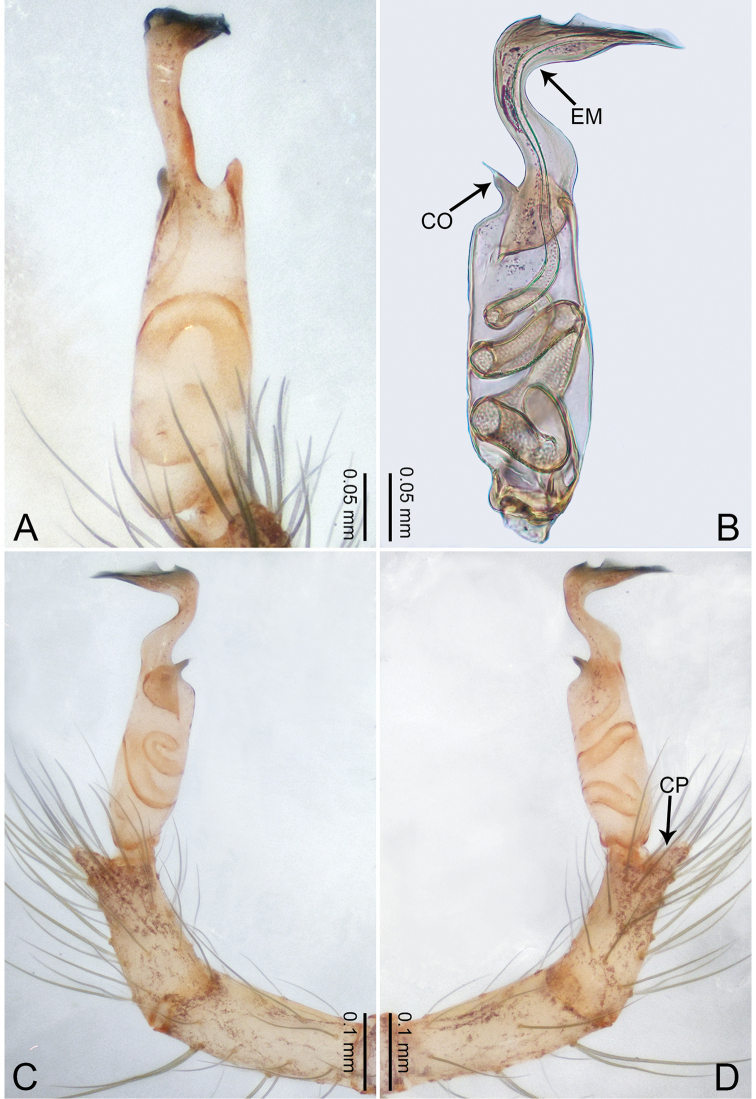
*Merizocera kachin* sp. nov., holotype male. **A** Bulb, dorsal view **B** bulb, retrolateral view **C** palp, prolateral view **D** palp, retrolateral view. Abbreviations: CO = conductor, CP = cymbial protrusion, EM = embolus.

**Figure 12.**
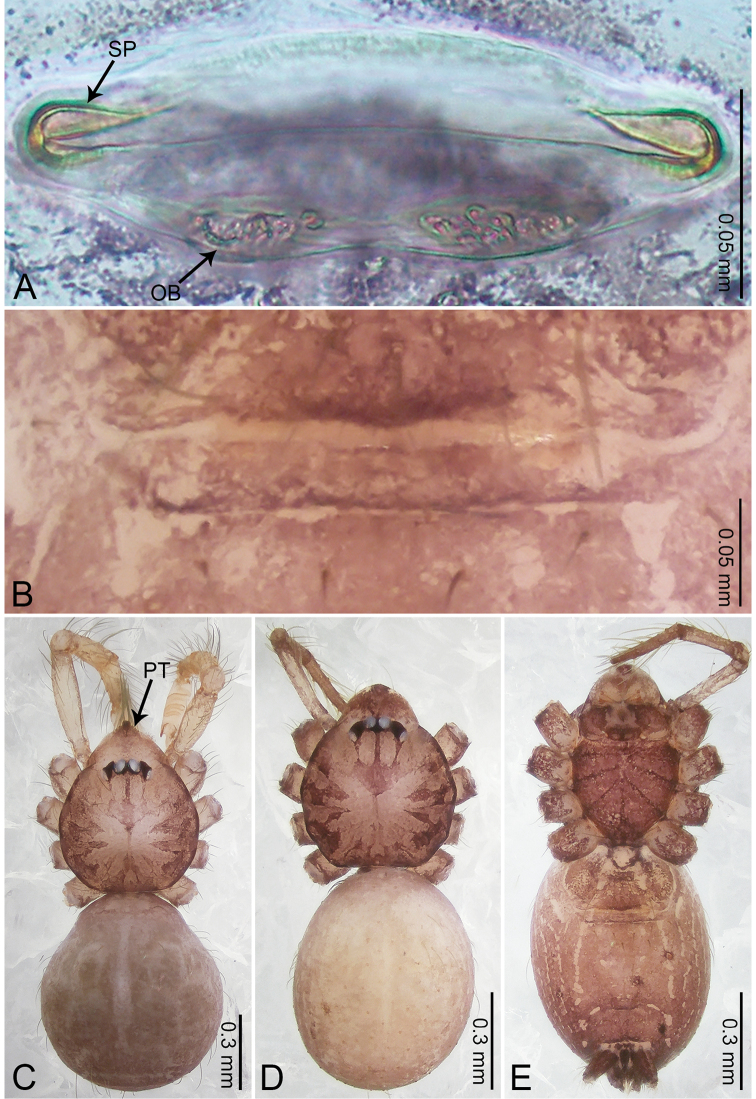
*Merizocera kachin* sp. nov., holotype male and paratype female. **A** Endogyne, dorsal view **B** female epigastric area, ventral view **C** male habitus, dorsal view **D** female habitus, dorsal view **E** female habitus, ventral view. Abbreviations: OB = ovoid body, PT = clypeal protrusion, SP = spermatheca.

Figures 11 , 12 , 53

##### Type material.

***Holotype***: male (IZCAS), Roadside between Nahteukhu and BaAve (27°18.00'N, 97°23.27'E, elevation 535 m), Putao, **Kachin State**, **Myanmar**, 8 December 2016, J. Wu leg. ***Paratype***: 1 female (IZCAS), same data as holotype.

##### Etymology.

The specific name refers to the type locality; noun in apposition.

##### Diagnosis.

Males resemble *M. putao* sp. nov. but can be distinguished by the absence of a pit on the bulb (vs. presence of a distinct pit resulting from the basal connection of embolus and conductor (Fig. 29B)), presence of a relatively short clypeal protrusion (Fig. 12C) (vs. relatively long clypeal protrusion (Fig. 30C). The females can be distinguished by a pair of conically tipped spermathecae (Fig. 12A) (vs. spermathecae each with a rounded tip (Fig. 30A)).

##### Description.

**Male** (holotype). Total length 1.44; carapace 0.67 long, 0.56 wide; abdomen 0.79 long, 0.74 wide. Carapace circular, brownish, with dark brown radiating marks (Fig. 12C). Fovea shallow. Thoracic region distinctly elevated medially. Clypeus brownish, with small protrusion provided with long setae. Labium dark brown. Sternum dark brown, with distinct dark radiating lines. Abdomen ovoid, dark grey (Fig. 12C). Legs light brown; measurements: I 6.87 (1.86, 0.21, 2.10, 1.92, 0.78), II 5.25 (1.44, 0.20, 1.60, 1.38, 0.63), III 3.85 (1.05, 0.19, 1.10, 1.00, 0.51), IV 5.58 (1.45, 0.21, 1.72, 1.45, 0.75). Palp (Fig. 11A–D): femur slender, four times longer than patella; patella not swollen; tibia half length of femur; cymbium with distal protrusion, half length of femur, length ratio of dorsal elongation and cymbium 0.28; bulb pale yellow, elongated pyriform with embolus and conductor arising distally; embolus hooked, almost similar in length with tegular, with pointed tip, width of anterior horizontal hooked part half width of tegular; conductor short with pointed tip, basally connected with embolus.

**Female** (paratype). General features and colouration similar to those of male except for the absence of clypeus protrusion (Fig. 12D, E). Measurements: total length 1.25; carapace 0.58 long, 0.49 wide; abdomen 0.69 long, 0.57 wide. Leg measurements: I 3.52 (0.89, 0.19, 1.06, 0.85, 0.53), II 2.95 (0.75, 0.19, 0.85, 0.70, 0.46), III 2.41 (0.63, 0.16, 0.63, 0.59, 0.40), IV 3.49 (0.87, 0.18, 1.05, 0.83, 0.56). Epigastric area (Fig. 12B): brown semi-circular patch with pale slit medially. Endogyne (Fig. 12A) with a pair of spermathecae each with a conical tip, posteriorly with a pair of ovoid bodies, ratio of the width of conical tip spermathecae to the interdistance of spermathecae 1:7.

##### Distribution.

Known only from the type locality (Myanmar; Fig. 53).

#### Merizocera kandy

Taxon classificationAnimaliaAraneaePsilodercidae

Li sp. nov.

880168BF-93C5-5121-99EF-8602599499AF

http://zoobank.org/6D7BEBE2-66DE-4F46-BBF3-A0212C24E21D

**Figure 13.**
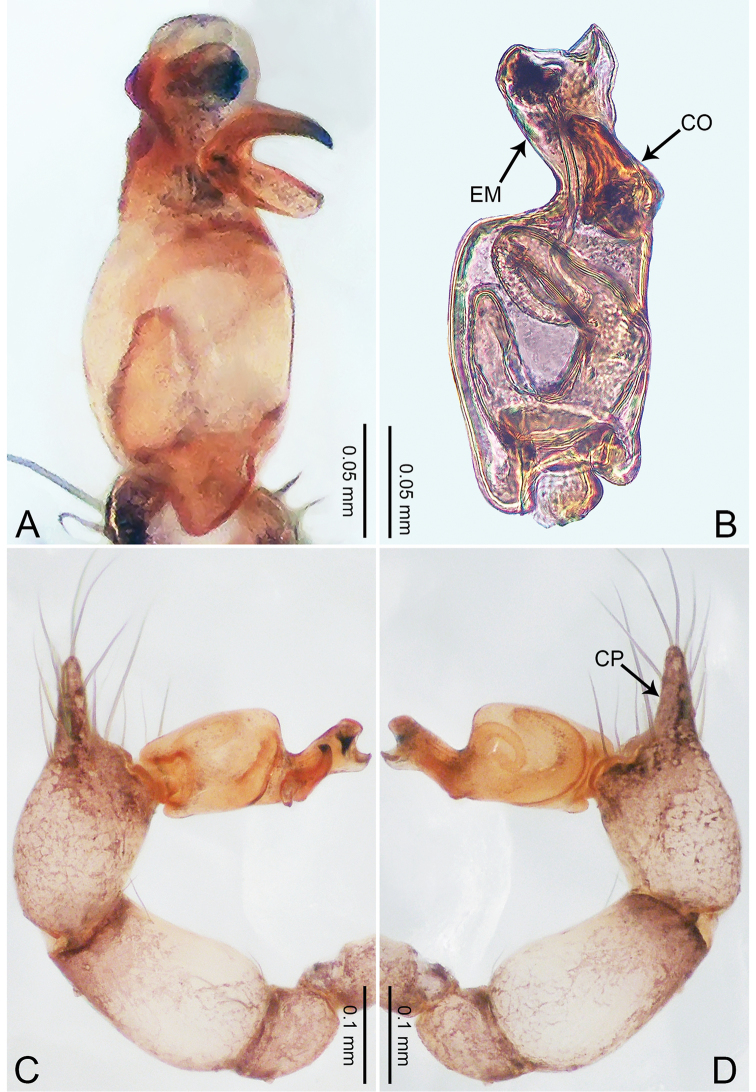
*Merizocera kandy* sp. nov., holotype male. **A** Bulb, dorsal view **B** bulb, prolateral view **C** palp, prolateral view **D** palp, retrolateral view. Abbreviations: CO = conductor, CP = cymbial protrusion, EM = embolus.

**Figure 14.**
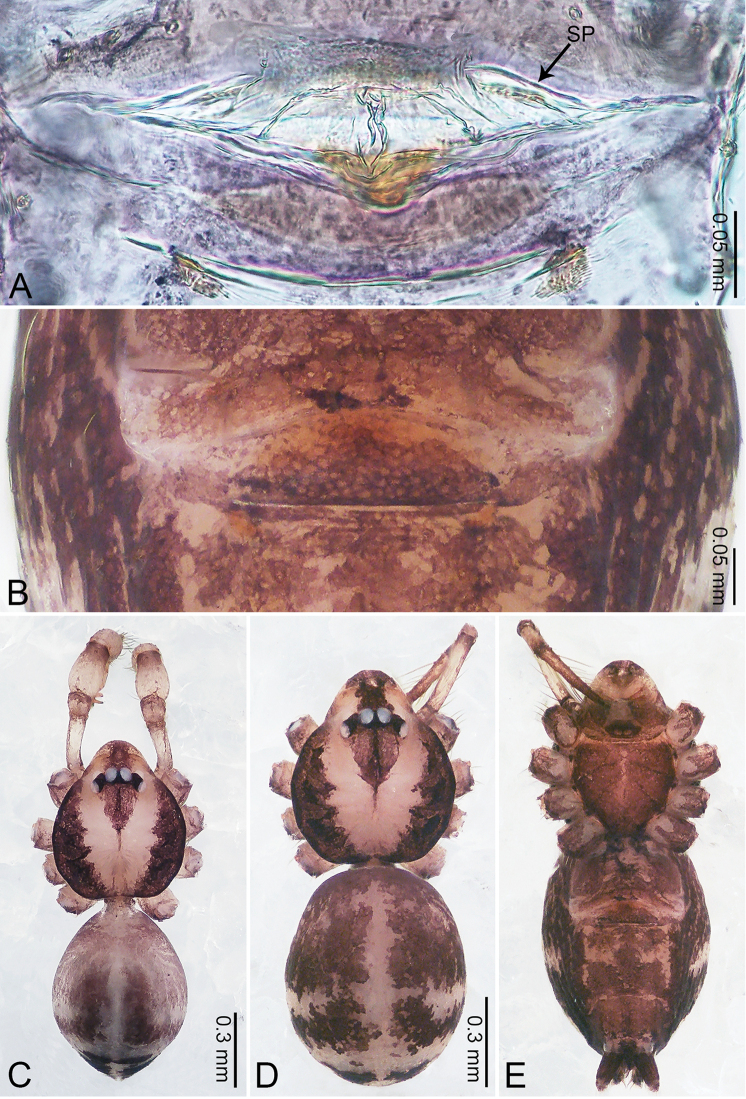
*Merizocera kandy* sp. nov., holotype male and paratype female. **A** Endogyne, dorsal view **B** female epigastric area, ventral view **C** male habitus, dorsal view **D** female habitus, dorsal view **E** female habitus, ventral view. Abbreviation: SP = spermatheca.

Figures 13 , 14 , 52

##### Type material.

***Holotype***: male (IZCAS), Koththol Lena (= cave) (6°54.22'N, 80°29.88'E, elevation 669 m), Abagamuwa Division, Maskeliya Oya Village, Maskeliya, Adam’s Peak Area, Kandy District, **Central Province**, **Sri Lanka**, 6 October 2014, S. Kosala leg. ***Paratypes***: 1 male and 1 female (IZCAS), same data as holotype.

##### Etymology.

The specific name refers to the type locality; noun in apposition.

##### Diagnosis.

Males can be distinguished from other congeners by the blunt bifurcate embolus tip, two similar components of conductor, and swollen bulb with a notch anteriorly (Fig. 13B). The females can be distinguished by a pair of angular shaped tubular spermathecae (Fig. 14A).

##### Description.

**Male** (holotype). Total length 1.33; carapace 0.63 long, 0.54 wide; abdomen 0.63 long, 0.56 wide. Carapace rounded, brownish, with dark brown marks laterally and dark median stripe on anterior half (Fig. 14C). Fovea shallow. Thoracic region distinctly elevated medially. Clypeus brownish with dark brown marks medially. Labium dark brown. Sternum dark brown with distinct dark radiating lines. Abdomen ovoid, brownish, with dark brown marks dorsally and ventrally. Legs brown; measurements: I 6.48 (1.70, 0.19, 1.96, 1.76, 0.87), II 4.51 (1.19, 0.20, 1.30, 1.18, 0.64), III 3.66 (0.98, 0.20, 1.05, 0.96, 0.47), IV missing. Palp (Fig. 13A–D): femur slender, thrice longer than patella, patella not swollen; tibia swollen, twice wider than and almost similar in length to femur; cymbium with distal protrusion, half as long as femur, length ratio of dorsal elongation and cymbium 0.59; bulb brown, with embolus and conductor arising distally, tegular with a notch anteriorly; embolus bifurcated with blunt tips, two similar components of conductor resemble a C-shape attached adjacent to embolus.

**Female** (paratype). General features and colouration similar to those of male (Fig. 14D, E). Measurements: total length 1.39; carapace 0.65 long, 0.56 wide; abdomen 0.72 long, 0.61 wide. Leg measurements: I missing, II 3.46 (0.90, 0.19, 0.99, 0.85, 0.53), III 2.96 (0.76, 0.19, 0.81, 0.75, 0.45), IV 4.40 (1.10, 0.20, 1.30, 1.14, 0.66). Epigastric area (Fig. 14B): elliptical dark brown patch. Endogyne (Fig. 14A) with a pair sloped tubular spermathecae, medially with vertical spiral ducts.

##### Distribution.

Known only from the type locality (Sri Lanka; Fig. 52).

#### Merizocera mandai

Taxon classificationAnimaliaAraneaePsilodercidae

Li sp. nov.

7D71FA60-8339-52C1-8151-6E174CD7D3AF

http://zoobank.org/F8BB7D25-0E42-443C-A087-F16B0C4CB036

**Figure 15.**
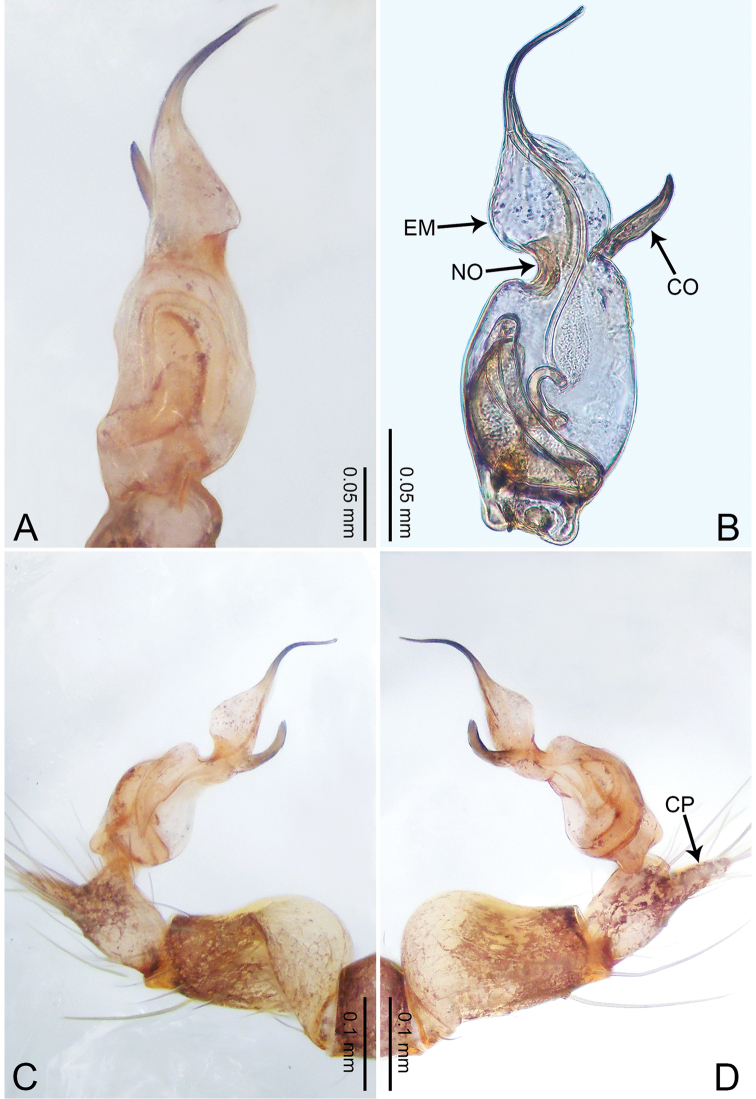
*Merizocera mandai* sp. nov., holotype male. **A** Bulb, dorsal view **B** bulb, prolateral view **C** palp, prolateral view **D** palp, retrolateral view. Abbreviations: CO = conductor, CP = cymbial protrusion, EM = embolus, NO = notch.

**Figure 16.**
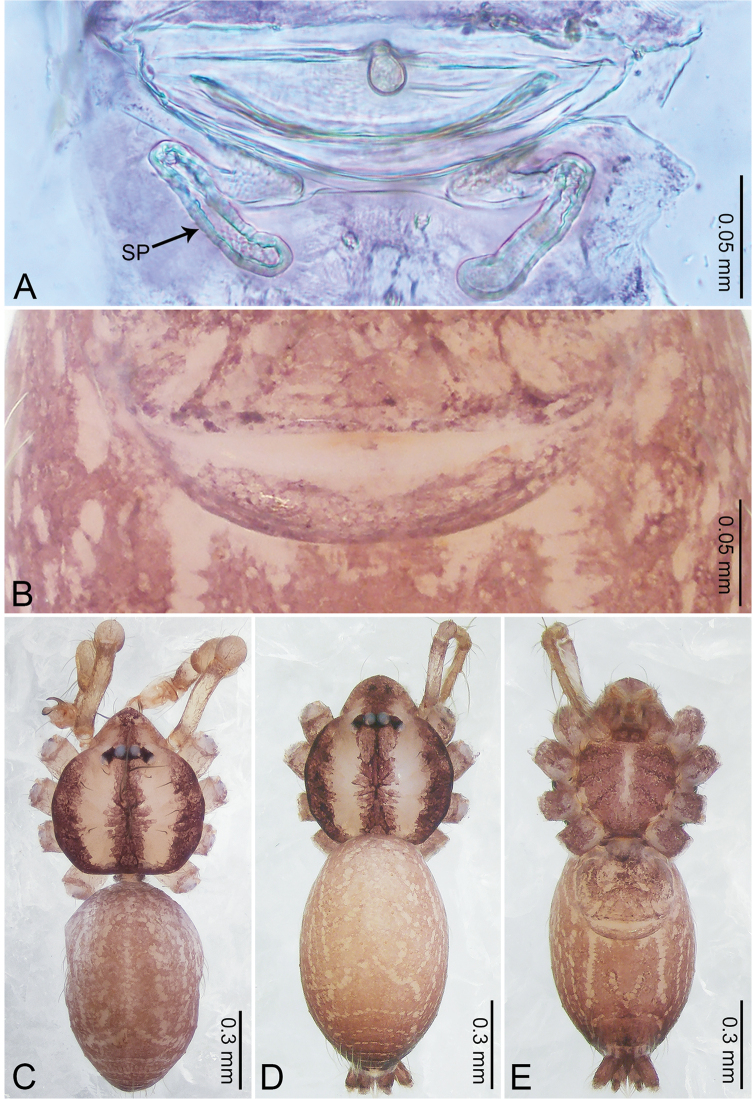
*Merizocera mandai* sp. nov., holotype male and paratype female. **A** Endogyne, dorsal view **B** female epigastric area, ventral view **C** male habitus, dorsal view **D** female habitus, dorsal view **E** female habitus, ventral view. Abbreviation: SP = spermatheca.

Figures 15 , 16 , 54

Merizocera sp. 279: Li and Li 2018 (molecular data).

##### Type material.

***Holotype***: male (IZCAS), near Mandai Agrotechnology Park (1°24.90'N, 103°47.94'E, elevation 46 m), Central Catchment Nature Reserve, **Singapore**, 1 September 2015, S. Li and Y. Tong leg. ***Paratypes***: 2 females (IZCAS), same data as holotype.

##### Etymology.

The specific name refers to the type locality; noun in apposition. Mandai is an important biodiversity conservation area in Singapore.

##### Diagnosis.

Males resemble *M. salawa* sp. nov. but can be distinguished by strongly swollen palpal tibia (Fig. 15D) (vs. palpal tibia not swollen (Fig. 35D)), presence of swollen embolus base (Fig. 15B) (vs. normal embolus base (Fig. 35B)), conductor tip not divided (Fig. 15B) (vs. bifurcate (Fig. 35B)), bulb with a notch (Fig. 15B) (vs. without notch (Fig. 35B)). The females can be distinguished by a pair of slight twisted, stalked spermathecae each bearing a globose distal part, directed downwards (Fig. 16A).

##### Description.

**Male** (holotype). Total length 1.41; carapace 0.62 long, 0.59 wide; abdomen 0.76 long, 0.54 wide. Carapace circular, brownish, with dark brown marks laterally and dark brown median stripe (Fig. 16C). Fovea shallow. Thoracic region distinctly elevated medially. Clypeus and labium dark brown. Sternum dark brown but lighter medially, with dark radiating lines. Abdomen slightly elongated, dark brown. Legs brown; measurements: I and II missing, III 4.09 (1.11, 0.19, 1.24, 1.05, 0.50), IV 6.30 (1.64, 0.20, 1.94, 1.68, 0.84). Palp (Fig. 15A–D): femur slender, thrice longer than patella, patella not swollen; tibia swollen proximally, half as long as femur; cymbium with distal protrusion, 1/3 femur length, length ratio of dorsal elongation and cymbium 0.67; bulb pale yellow, embolus and conductor arising distally, tegular with a notch anteriorly; embolus basally swollen, swollen part occupies half length of embolus and almost half width of bulb; conductor basally connected with embolus, tentacle-like, 1/3 length of embolus.

**Female** (paratype). General features and colouration similar to those of male (Fig. 16D, E). Measurements: total length 1.41; carapace 0.58 long, 0.51 wide; abdomen 0.84 long, 0.51 wide. Leg measurements: I and IV missing, II 3.97 (1.05, 0.19, 1.14, 1.00, 0.59), III 3.10 (0.83, 0.18, 0.82, 0.79, 0.48). Epigastric area (Fig. 16B): brown crescent-shaped patch. Endogyne (Fig. 16A) with a pair of slightly twisted stalked spermathecae, bearing globose distal ends pointed posteriorly, anterior-medially with a spherical structure.

##### Distribution.

Known only from the type locality (Singapore; Fig. 54).

#### Merizocera krabi

Taxon classificationAnimaliaAraneaePsilodercidae

Li sp. nov.

57938962-DE99-5620-94AC-D51DD8C5B47A

http://zoobank.org/6EDA6125-F3EF-4D4D-AF5C-F78E2301B303

**Figure 17.**
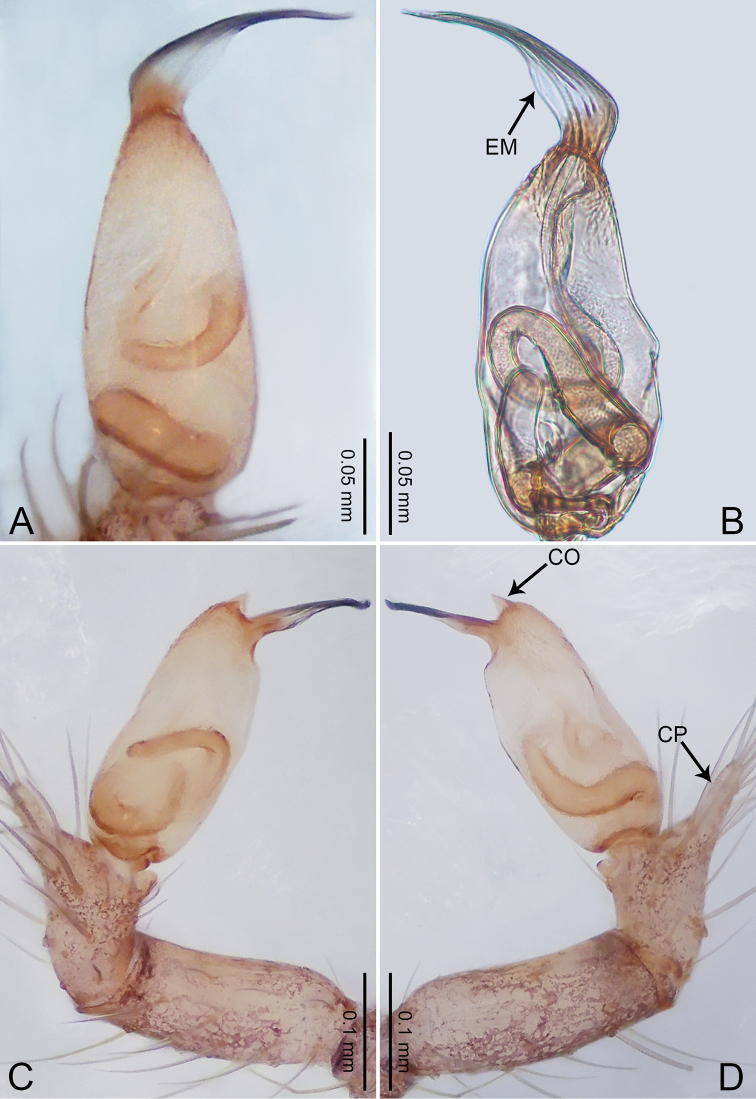
*Merizocera krabi* sp. nov., holotype male. **A** Bulb, dorsal view **B** bulb, ventral view **C** palp, prolateral view **D** palp, retrolateral view. Abbreviations: CO = conductor, CP = cymbial protrusion, EM = embolus.

**Figure 18.**
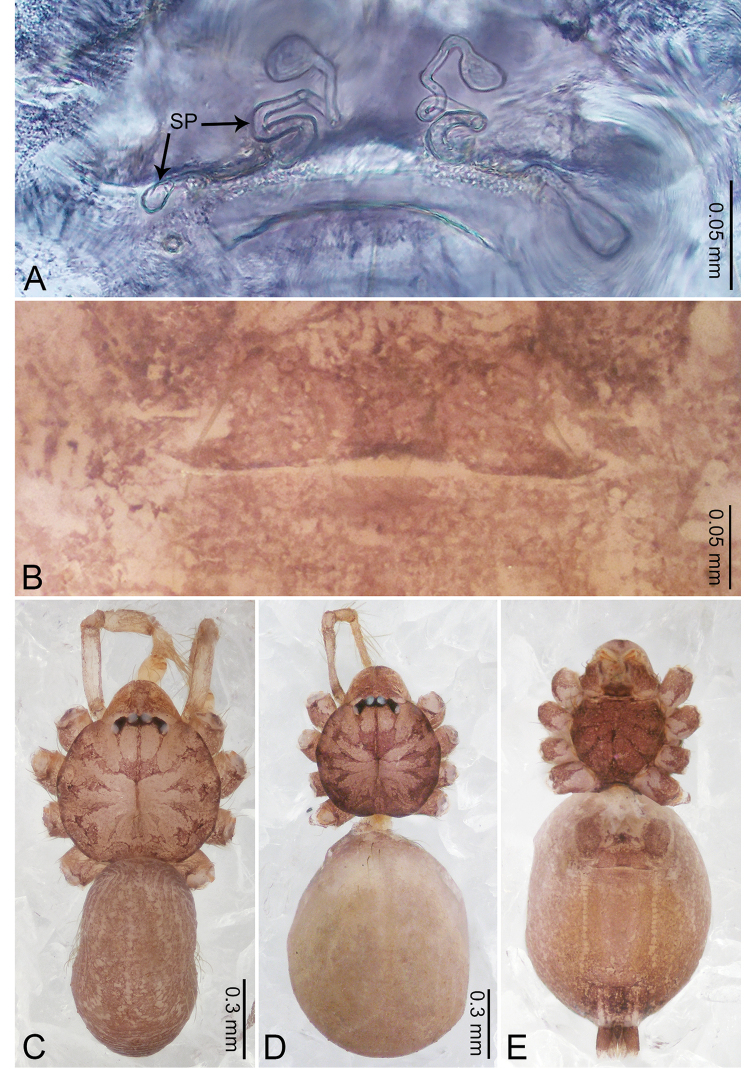
*Merizocera krabi* sp. nov., holotype male and paratype female. **A** Endogyne, dorsal view **B** female epigastric area, ventral view **C** male habitus, dorsal view **D** female habitus, dorsal view **E** female habitus, ventral view. Abbreviation: SP = spermatheca.

Figures 17 , 18 , 54

Merizocera sp. 185: Li and Li 2018 (molecular data).

##### Type material.

***Holotype***: male (IZCAS), Ban Chong Plee Village (8°5.12'N, 98°51.22'E, elevation 442 m), Muang District, **Krabi**, **Thailand**, 25 October 2014, P. Wongprom leg. ***Paratype***: 1 female (IZCAS), same data as holotype.

##### Etymology.

The specific name refers to the type locality; noun in apposition.

##### Diagnosis.

Diagnosis features of males are discussed under *M. hponkanrazi* sp. nov. Males can be distinguished by an elongated pyriform bulb with a triangular conductor adjacent to the base of the embolus (Fig. 17D), embolus approx. half as long as the tegulum. The females can be distinguished from congeners by two pairs of twisted stalked spermathecae bearing globose distal ends (Fig. 18A).

##### Description.

**Male** (holotype). Total length 1.44; carapace 0.72 long, 0.63 wide; abdomen 0.72 long, 0.48 wide. Carapace circular, brown, with dark brown radiating marks (Fig. 18C). Fovea shallow. Thoracic region distinctly elevated medially. Clypeus and labium dark brown. Sternum dark brown, with dark radiating lines. Abdomen slightly elongated, dark brown. Legs brown; measurements: I and IV missing, II 6.50 (1.84, 0.23, 2.00, 1.68, 0.75), III 4.83 (1.39, 0.23, 1.47, 1.19, 0.55). Palp (Fig. 17A–D): femur slender, four times longer than patella; patella not swollen; tibia not swollen; cymbium with distal protrusion, 1/3 femur length, length ratio of dorsal elongation and cymbium 0.88; bulb pale yellow, elongated pyriform-shaped with embolus and conductor arising distally; embolus bent with laminar base, laminar part 1/2 length of embolus, entire embolus 1/2 length of tegular; conductor triangular, adjacent to embolus, 1/5 length of embolus.

**Female** (paratype). General features and colouration similar to those of male (Fig. 18D, E). Measurements: total length 1.70; carapace 0.67 long, 0.54 wide; abdomen 0.98 long, 0.82 wide. Leg measurements: I 5.44 (1.44, 0.19, 1.66, 1.38, 0.77), II missing, III 3.43 (0.90, 0.19, 1.00, 0.83, 0.51), IV 4.89 (1.28, 0.19, 1.48, 1.25, 0.69). Epigastric area (Fig. 18B): dark brown, semi-circular. Endogyne (Fig. 18A) with two pairs of twisted stalked spermathecae with globose distal ends, lateral pairs almost horizontal, median pairs upright.

##### Distribution.

Known only from the type locality (Thailand; Fig. 54).

#### Merizocera kurunegala

Taxon classificationAnimaliaAraneaePsilodercidae

Li sp. nov.

98C2AE35-D4F3-5233-82C0-390BA848F9DD

http://zoobank.org/B7C22440-1A06-4347-9F4E-900002716789

**Figure 19.**
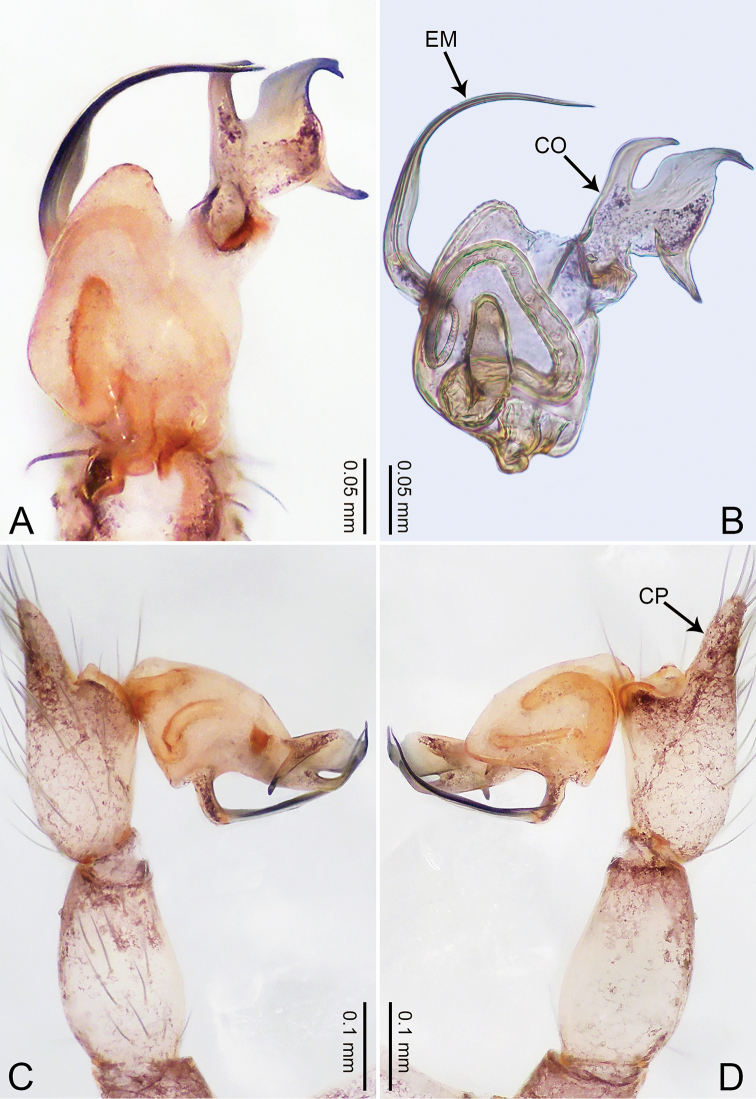
*Merizocera kurunegala* sp. nov., holotype male. **A** Bulb, dorsal view **B** bulb, dorsal view **C** palp, prolateral view **D** palp, retrolateral view. Abbreviations: CO = conductor, CP = cymbial protrusion, EM = embolus.

**Figure 20.**
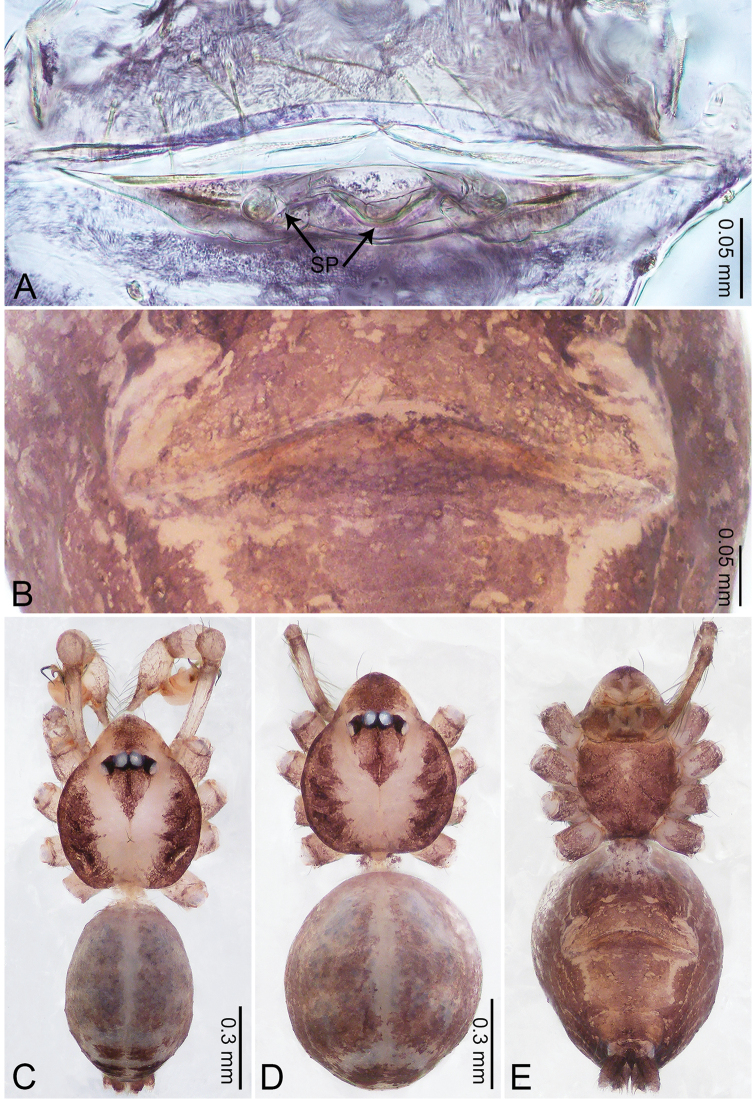
*Merizocera kurunegala* sp. nov., holotype male and paratype female. **A** Endogyne, dorsal view **B** female epigastric area, ventral view **C** male habitus, dorsal view **D** female habitus, dorsal view **E** female habitus, ventral view. Abbreviation: SP = spermatheca.

Figures 19 , 20 , 52

##### Type material.

***Holotype***: male (IZCAS), near Arankele Cave (7°38.42'N, 80°25.33'E, elevation 114 m), Kubukwewa Village, Hiripitiya, Kurunegala District, **Northwestern Province**, **Sri Lanka**, 11 October 2014, S. Kosala leg. ***Paratype***: 1 female (IZCAS), same data as holotype.

##### Etymology.

The specific name refers to the type locality; noun in apposition.

##### Diagnosis.

Males resemble *M. peraderiya* sp. nov. and *M. picturata* but can be distinguished by a relatively shorter bulb (Fig. 19B) (1/2 length of that in *M. peraderiya* sp. nov. (Fig. 25B) and *M. picturata*), an evenly bent embolus in *M. kurunegala* sp. nov. (Fig. 19B) and *M. picturata* (vs. an angularly bent embolus in *M. peraderiya* sp. nov. (Fig. 25B)), furcate conductor in *M. kurunegala* sp. nov. (Fig. 19B) and *M. peraderiya* sp. nov. (Fig. 25B) (vs. conductor not furcate in *M. picturata*), conductor arising medially in *M. picturata* (vs. conductor not arising medially in *M. kurunegala* sp. nov. (Fig. 19B) and *M. peraderiya* sp. nov. (Fig. 25B)). The females can be distinguished by having sessile wavy spermathecae in *M. kurunegala* sp. nov. (Fig. 20A) and *M. picturata* (vs. a pair of S-shaped spermathecae in *M. peraderiya* sp. nov. (Fig. 26A)).

##### Description.

**Male** (holotype). Total length 1.33; carapace 0.67 long, 0.53 wide; abdomen 0.65 long, 0.49 wide. Carapace circular, brownish, with dark brown marks laterally and dark brown median stripe on anterior half (Fig. 20C). Fovea shallow. Thoracic region distinctly elevated medially. Clypeus brownish, with dark brown marks medially. Labium and sternum dark brown. Abdomen ovoid, dark grey, with dark brown marks dorsally and ventrally. Legs brown; measurements: I 7.14 (1.94, 0.22, 2.20, 1.96, 0.82), II missing, III 3.66 (1.03, 0.19, 1.05, 0.95, 0.44), IV 5.87 (1.53, 0.19, 1.80, 1.62, 0.73). Palp (Fig. 19A–D): femur slender, thrice longer than patella, patella not swollen; tibia slightly swollen proximally, twice wider and half length of femur; cymbium with distal protrusion, half length of femur, length ratio of dorsal elongation and cymbium 0.64; bulb pale yellow; embolus dark, evenly bent, twice as long as bulb; conductor trifurcate with two pointed upwards, and third pointed downwards, stem of conductor thrice wider than that of embolus.

**Female** (paratype). General features and colouration similar to those of male (Fig. 20D, E). Measurements: total length 1.41; carapace 0.62 long, 0.53 wide; abdomen 0.76 long, 0.67 wide. Leg measurements: I 3.99 (1.00, 0.19, 1.20, 0.98, 0.62), II 3.11 (0.82, 0.19, 0.87, 0.75, 0.48), III missing, IV 3.84 (0.98, 0.18, 1.11, 0.99, 0.58). Epigastric area (Fig. 20B): dark brown nearly trapezoidal patch. Endogyne (Fig. 20A) with a pair of wavy spermathecae with globose distal ends.

##### Distribution.

Known only from the type locality (Sri Lanka; Fig. 52).

#### Merizocera lincang

Taxon classificationAnimaliaAraneaePsilodercidae

Li sp. nov.

F4A7E7FF-3EB3-585F-BCE5-C6B11AA45292

http://zoobank.org/DA6E2AD1-B1E6-4144-9DFA-11718FD84CE6

Figures 21 , 53

##### Type material.

***Holotype***: female (IZCAS), Qingquan Cave (23°52.16'N, 99°12.42'E, elevation 295 m), Minglang Town, Yongde County, Lincang, **Yunnan**, **China**, 7 August 2015, Y. Li and Z. Chen leg. ***Paratypes***: 2 females (IZCAS), same data as holotype.

##### Etymology.

The specific name refers to the type locality; noun in apposition.

##### Diagnosis.

**Figure 21.**
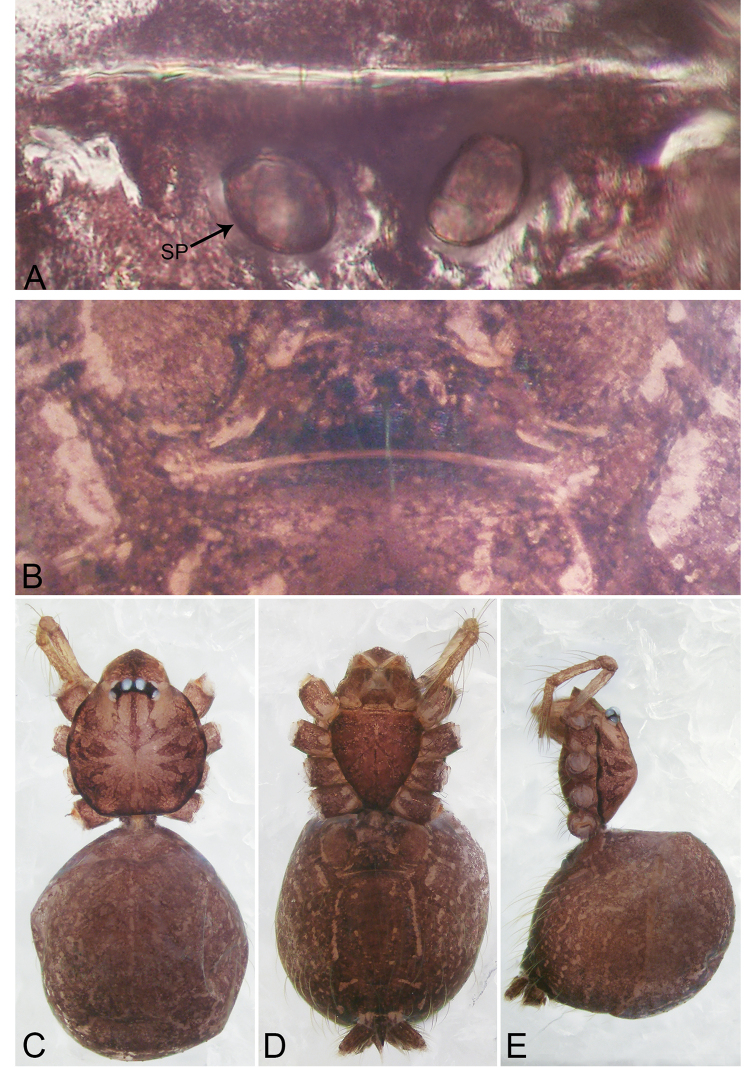
*Merizocera lincang* sp. nov., holotype female. **A** Endogyne, dorsal view **B** female epigastric area, ventral view **C** female habitus, dorsal view **D** female habitus, ventral view **E** female habitus, lateral view. Abbreviation: SP = spermatheca.

Females of *M. lincang* sp. nov. can be distinguished from all congeners by a pair of large bulbous spermathecae (Fig. 21A).

##### Description.

**Female** (holotype). Total length 1.58; carapace 0.64 long, 0.53 wide; abdomen 0.92 long, 0.81 wide. Carapace circular, brown, with dark brown radiating marks (Fig. 21C). Fovea shallow. Thoracic region distinctly elevated medially. Clypeus, labium, and sternum dark brown. Abdomen ovoid, dark brown (Fig. 21B). Legs brown; measurements: I 3.50 (0.88, 0.20, 1.05, 0.81, 0.56), II 3.04 (0.79, 0.20, 0.87, 0.70, 0.48), III 2.58 (0.67, 0.17, 0.67, 0.63, 0.44), IV 3.71 (0.92, 0.19, 1.10, 0.88, 0.62). Epigastric area (Fig. 21B): dark brown, nearly trapezoidal patch. Endogyne (Fig. 21A): ratio of the width of spermathecae to the interdistance of spermathecae 1:3.

**Male.** Unknown.

##### Distribution.

Known only from the type locality (China; Fig. 53).

#### Merizocera mainling

Taxon classificationAnimaliaAraneaePsilodercidae

Li sp. nov.

E522561B-A91F-5EE4-B5D8-38F71113A721

http://zoobank.org/598BF801-F7D7-42BB-9219-1A2838068922

**Figure 22.**
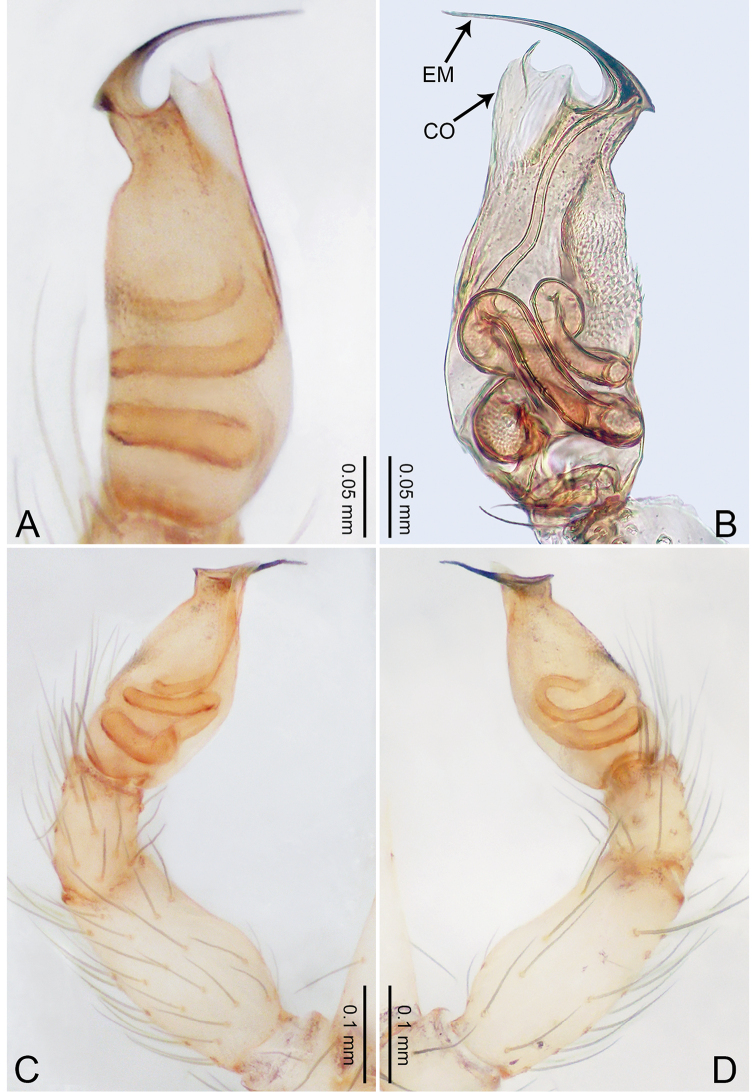
*Merizocera mainling* sp. nov., holotype male. **A** Bulb, dorsal view **B** bulb, ventral view **C** palp, prolateral view **D** palp, retrolateral view. Abbreviations: CO = conductor, EM = embolus.

**Figure 23.**
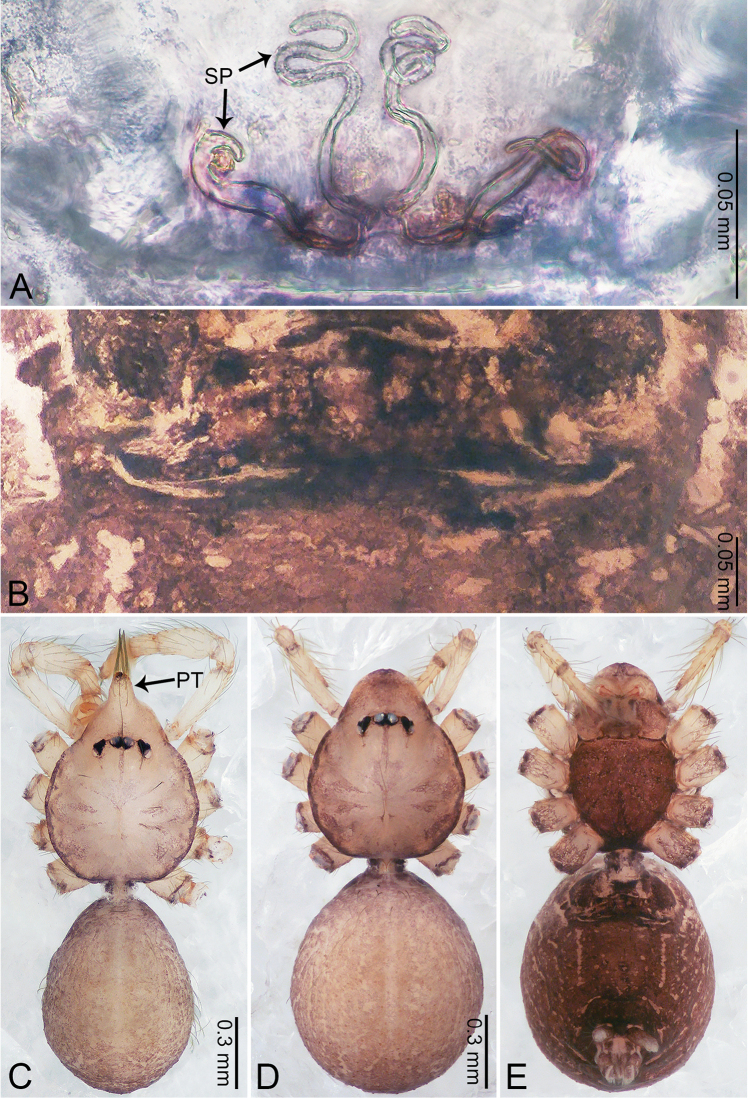
*Merizocera mainling* sp. nov., holotype male and paratype female. **A** Endogyne, dorsal view **B** female epigastric area, ventral view **C** male habitus, dorsal view **D** female habitus, dorsal view **E** female habitus, ventral view. Abbreviations: PT = clypeal protrusion, SP = spermatheca.

Figures 22 , 23 , 53

Merizocera sp. 46: Li and Li 2018 (molecular data).

##### Type material.

***Holotype***: male (IZCAS), northern Mainling County (29°13.31'N, 94°13.31'E, elevation 3050 m), Nyingchi, **Tibet**, **China**, 13 August 2013, L. Lin leg. ***Paratypes***: 1 male and 1 female (IZCAS), same data as holotype.

##### Etymology.

The specific name refers to the type locality; noun in apposition.

##### Diagnosis.

Males resemble *M. tanintharyi* sp. nov. but can be distinguished by a thin darkened embolus (Fig. 22B) (vs. thick and dark only at tip (Fig. 38A)), conductor lamina-like and shorter than embolus (Fig. 22B) (vs. conductor appendage-like and similar length as embolus (Fig. 38B)), presence of clypeus protrusion (Fig. 23C) (vs. absence of clypeal protrusion), absence of cymbial protrusion (vs. presence of cymbial protrusion (Fig. 38D)). The females can be distinguished by two pairs of twisted spermathecae (Fig. 23A) (vs. three pairs of short tubular spermathecae (Fig. 39A)).

##### Description.

**Male** (holotype). Total length 1.72; carapace 0.84 long, 0.65 wide; abdomen 0.82 long, 0.66 wide. Carapace circular, brownish, with dark brown radiating marks (Fig. 23C). Fovea shallow. Thoracic region distinctly elevated medially. Clypeus brownish, with large protrusion provided with long setae (Fig. 23C). Labium and sternum dark brown. Abdomen ovoid, dark brown (Fig. 23E). Legs light brown; measurements: I 6.34 (1.70, 0.26, 1.92, 1.68, 0.78), II missing, III 3.74 (1.05, 0.22, 1.05, 0.93, 0.49), IV 5.10 (1.36, 0.24, 1.53, 1.27, 0.70). Palp (Fig. 22A–D): femur slender, thrice longer than patella; patella not swollen; tibia slightly swollen proximally; cymbium without dorsal protrusion, 1/3 femur length; bulb pale yellow, pyriform with embolus and conductor arising distally; embolus thin and dark, arising laterally, longer than the width of bulb; conductor lamina-like, half the width of bulb.

**Female** (paratype). General features and colouration similar to those of male (Fig. 23D, E). Measurements: total length 1.76; carapace 0.79 long, 0.65 wide; abdomen 0.94 long, 0.82 wide. Leg measurements: I 4.48 (1.18, 0.25, 1.38, 1.04, 0.63), II 3.70 (1.00, 0.24, 1.08, 0.85, 0.53), III 3.19 (0.89, 0.23, 0.87, 0.75, 0.45), IV 4.38 (1.15, 0.23, 1.33, 1.04, 0.63). Epigastric area (Fig. 23B): dark brown, nearly trapezoidal patch. Endogyne (Fig. 23A) with two pairs of long, twisted, stalked spermathecae with blunt ends.

##### Distribution.

Known only from the type locality (China; Fig. 53).

#### Merizocera nyingchi sp. nov.

Taxon classificationAnimaliaAraneaePsilodercidae

5E7A813C-917E-5007-A863-D25AED5A843D

http://zoobank.org/B1A4B9F0-9C4F-4E0E-B5D8-9EAC98A73FA6

Figures 24 , 53

##### Type material.

***Holotype***: female (IZCAS), mountain behind a farmhouse resort (29°19.09'N, 95°18.88'E, elevation 1280 m), Medog County, Nyingchi, **Tibet**, **China**, 4 August 2013, L. Lin leg. ***Paratype***: 1 female (IZCAS), same data as holotype.

##### Etymology.

The specific name refers to the type locality; noun in apposition.

##### Diagnosis.

**Figure 24.**
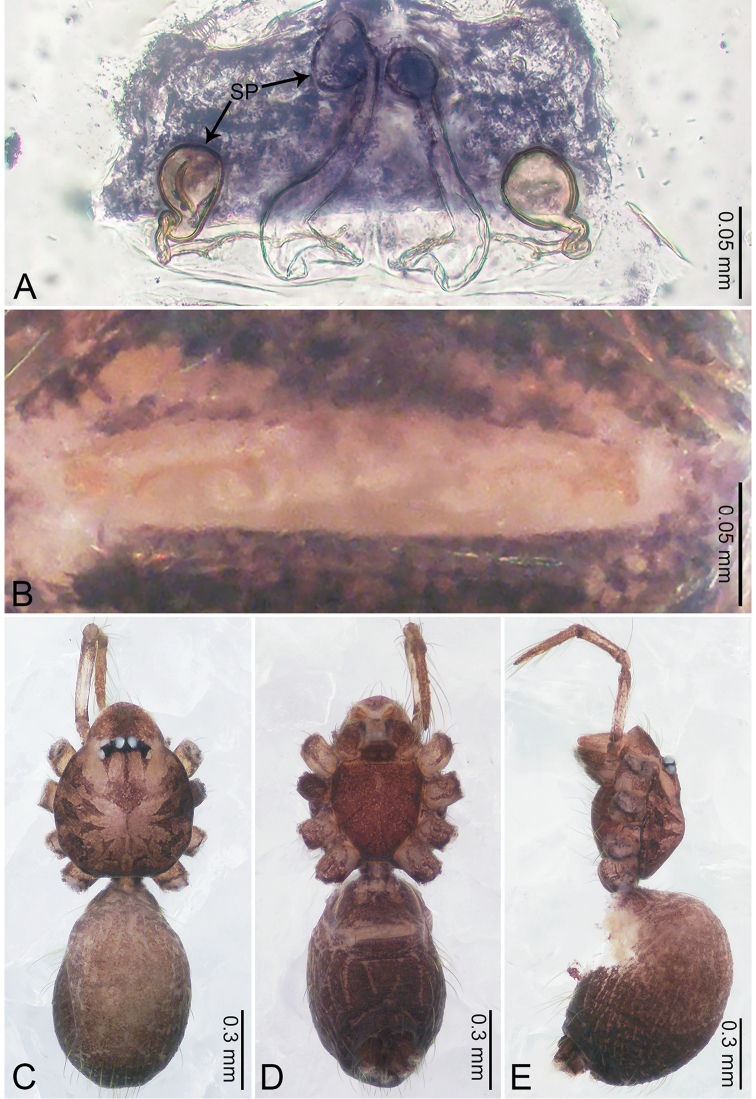
*Merizocera nyingchi* sp. nov., holotype female. **A** Endogyne, dorsal view **B** female epigastric area, ventral view **C** female habitus, dorsal view **D** female habitus, ventral view **E** female habitus, lateral view. Abbreviation: SP = spermatheca.

Females can be distinguished from congeners by two pairs of twisted stalked spermathecae bearing globose distal ends, where lateral pairs are at least half as short as median pairs and have globose ends twice the size of the former (Fig. 24A).

##### Description.

**Female** (holotype). Total length 1.42; carapace 0.65 long, 0.53 wide; abdomen 0.75 long, 0.53 wide. Carapace circular, brown, with dark brown radiating marks (Fig. 24C). Fovea shallow. Thoracic region distinctly elevated medially. Clypeus, labium, and sternum dark brown. Abdomen slightly elongated, dark brown (Fig. 24B). Legs light brown; measurements: I, II, and III missing, IV 4.06 (1.05, 0.19, 1.19, 1.00, 0.63). Epigastric area (Fig. 24B): dark brown patch, medially with pale yellow horizontal strip. Endogyne (Fig. 24A) with two pairs of twisted stalked spermathecae bearing globose distal ends, lateral pairs half as short as median pairs, globose ends of lateral pairs twice larger than median pairs.

**Male.** Unknown.

##### Distribution.

Known only from the type locality (China; Fig. 53).

#### Merizocera peraderiya

Taxon classificationAnimaliaAraneaePsilodercidae

Li sp. nov.

F944B7A9-F6E3-51DD-9368-BA3699E62B0A

http://zoobank.org/1773F0E5-4FF0-4028-AB88-4BF281731579

**Figure 25.**
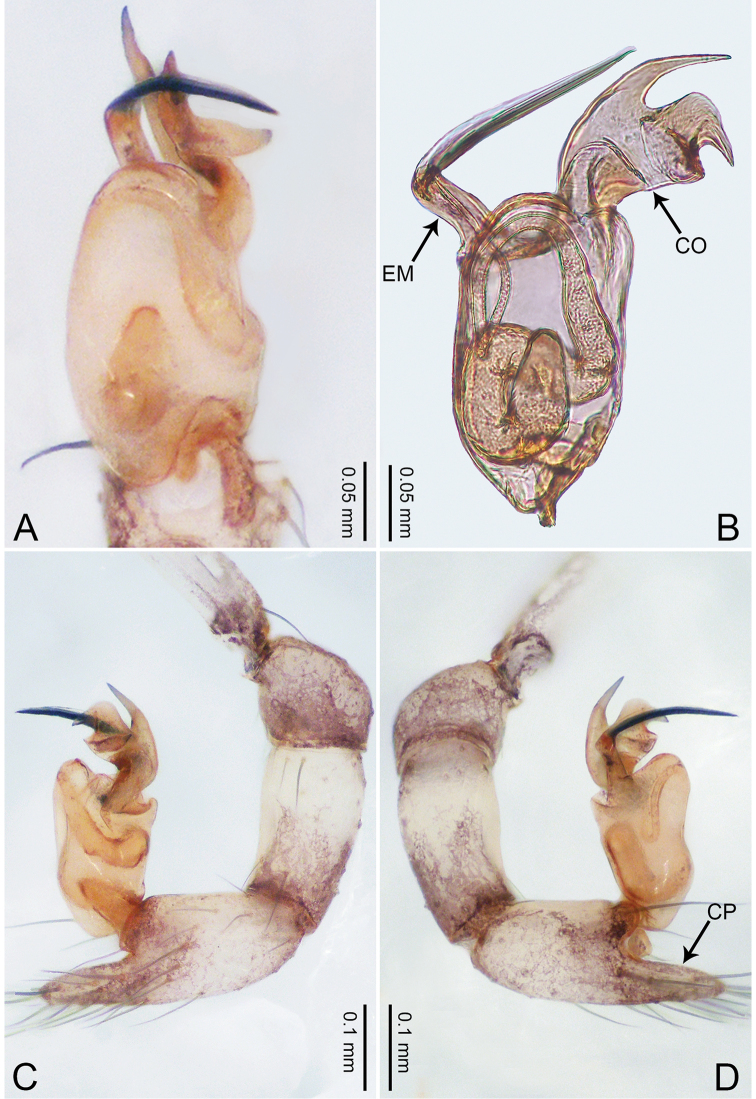
*Merizocera peraderiya* sp. nov., holotype male. **A** Bulb, dorsal view **B** bulb, dorsal view, embolus and conductor distorted **C** palp, prolateral view **D** palp, retrolateral view. Abbreviations: CO = conductor, CP = cymbial protrusion, EM = embolus.

**Figure 26.**
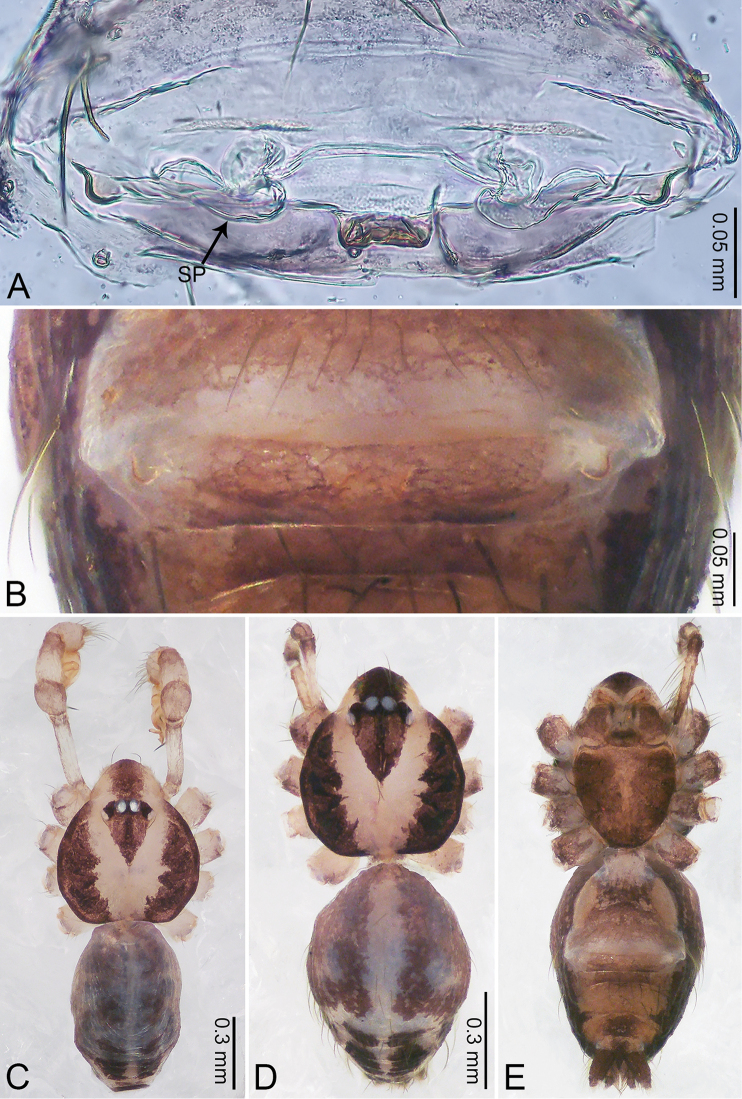
*Merizocera peraderiya* sp. nov., holotype male and paratype female. **A** Endogyne, dorsal view **B** female epigastric area, ventral view **C** male habitus, dorsal view **D** female habitus, dorsal view **E** female habitus, ventral view. Abbreviation: SP = spermatheca.

Figures 25 , 26 , 52

##### Type material.

***Holotype***: male (IZCAS), Royal Botanic Gardens (7°16.52'N, 80°35.71'E, elevation 484 m), Peraderiya Town, Kandy District, **Central Province**, **Sri Lanka**, 7 October 2014, S. Kosala leg. ***Paratype***: 1 female (IZCAS), same data as holotype.

##### Etymology.

The specific name refers to the type locality; noun in apposition.

##### Diagnosis.

Diagnosis features of males and females are discussed in *M. kurunegala* sp. nov. Males with angularly bent embolus and furcate conductor (Fig. 25B). Females with a pair of twisted spermathecae (Fig. 26A).

##### Description.

**Male** (holotype). Total length 1.33; carapace 0.66 long, 0.59 wide; abdomen 0.67 long, 0.46 wide. Carapace circular, brownish, with dark brown marks laterally and dark brown median stripe on anterior half (Fig. 26C). Fovea shallow. Thoracic region distinctly elevated medially. Clypeus brownish, with dark brown marks medially. Labium dark brown. Sternum dark brown but lighter medially. Abdomen slightly elongated, dark grey, with dark brown marks dorsally and ventrally. Legs light brown; measurements: I 7.81 (2.05, 0.22, 2.38, 2.20, 0.96), II 5.35 (1.41, 0.22, 1.60, 1.41, 0.71), III 4.11 (1.13, 0.19, 1.19, 1.06, 0.54), IV 6.49 (1.66, 0.21, 2.00, 1.78, 0.84). Palp (Fig. 25A–D): femur slender, 2.5 times longer than patella; patella not swollen; tibia slightly swollen, half as long as femur; cymbium with distal protrusion, length ratio of dorsal elongation and cymbium 0.72; bulb pale yellow, pyriform with embolus and conductor arising distally; embolus thin and dark with pointed tip, emerging laterally, bent at right-angle; conductor basally attached with embolus, bifurcate, one slender and slightly bent, the other hooked and twice as wide, conductor stem half as long as the width of bulb.

**Female** (paratype). General features and colouration similar to those of male (Fig. 26D, E). Measurements: total length 1.28; carapace 0.61 long, 0.51 wide; abdomen 0.69 long, 0.52 wide. Leg measurements: I 4.25 (1.06, 0.20, 1.28, 1.05, 0.66), II 3.25 (0.83, 0.19, 0.92, 0.80, 0.51), III 2.66 (0.67, 0.17, 0.71, 0.68, 0.43), IV missing. Epigastric area (Fig. 26B): dark brown, patch nearly elliptical. Endogyne (Fig. 26A) with a pair of twisted S-shaped spermathecae, ratio of the width of spermatheca to the interdistance of spermathecae 1:4.

##### Distribution.

Known only from the type locality (Sri Lanka; Fig. 52).

#### Merizocera phuket

Taxon classificationAnimaliaAraneaePsilodercidae

Li sp. nov.

B213F9A0-F598-5078-8DB8-260F9446E237

http://zoobank.org/01F5775C-4E6A-40F7-853A-EE6974F7037A

**Figure 27.**
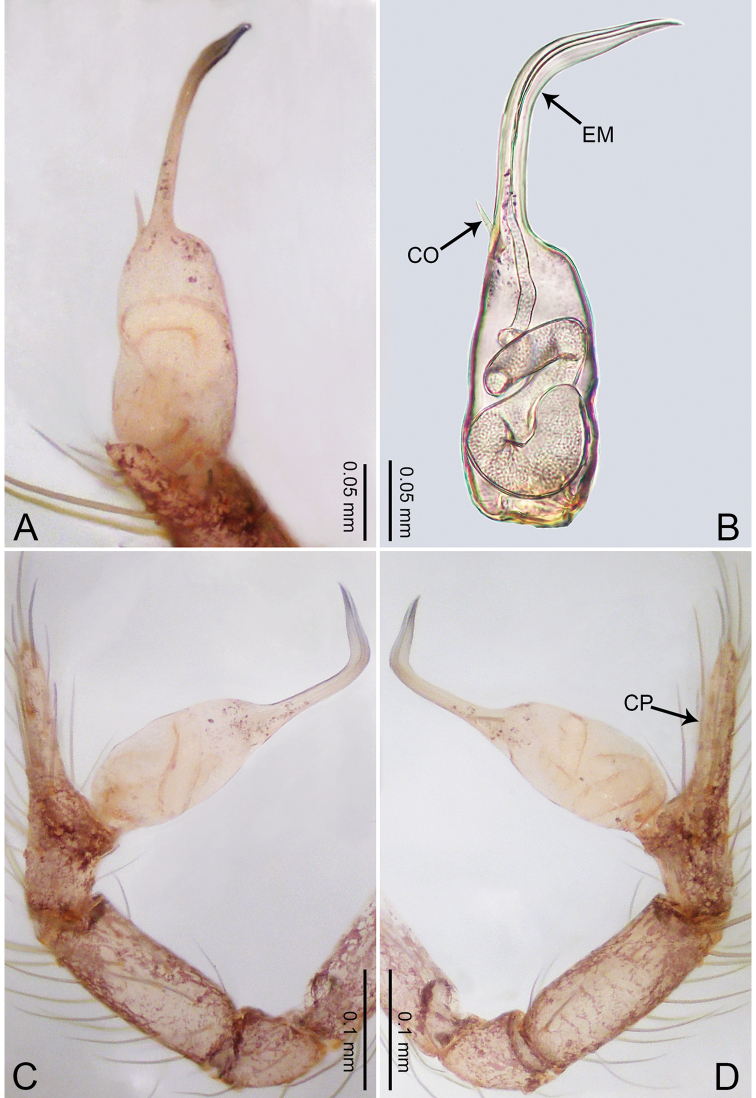
*Merizocera phuket* sp. nov., holotype male. **A** Bulb, dorsal view **B** bulb, retrolatero-dorsal view **C** palp, prolateral view **D** palp, retrolateral view. Abbreviations: CO = conductor, CP = cymbial protrusion, EM = embolus.

**Figure 28.**
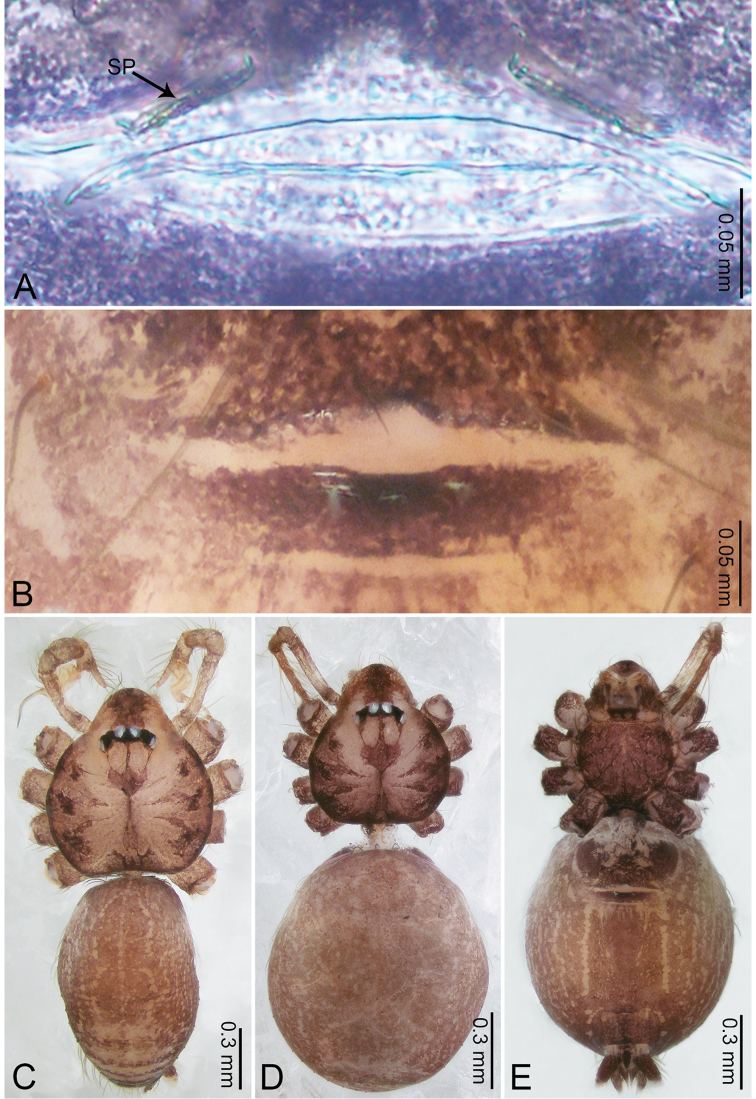
*Merizocera phuket* sp. nov., holotype male and paratype female. **A** Endogyne, dorsal view **B** female epigastric area, ventral view **C** male habitus, dorsal view **D** female habitus, dorsal view **E** female habitus, ventral view. Abbreviation: SP = spermatheca.

Figures 27 , 28 , 54

##### Type material.

***Holotype***: male (IZCAS), Toh Sae Mountain (7°53.96'N, 98°23.98'E, elevation 203 m), Mueang District, **Phuket**, **Thailand**, 29 October 2015, P. Wongprom leg. ***Paratypes***: 2 males and 2 females (IZCAS), same data as holotype.

##### Etymology.

The specific name refers to the type locality; noun in apposition.

##### Diagnosis.

Males resemble those of *M. ratnapura* sp. nov. but can be distinguished by the relatively thin and long conductor (Fig. 27B) (vs. relatively thick and short conductor (Fig. 33B)), embolus with consistent width (Fig. 27B) (vs. embolus gradually thinner towards tip (Fig. 33B)), embolus length similar to bulb length (Fig. 27B) (vs. embolus twice longer than bulb (Fig. 33B)). Females can be distinguished by their horizontally angled elongated spermathecae (Fig. 28A) (vs. wide tubular spermathecae with globose stalked spermatheca medially (Fig. 34A)).

##### Description.

**Male** (holotype). Total length 1.39; carapace 0.64 long, 0.59 wide; abdomen 0.72 long, 0.49 wide. Carapace circular, brownish, with dark brown radiating marks and narrow dark brown stripe (Fig. 28C). Fovea shallow. Thoracic region distinctly elevated medially. Clypeus brownish, with dark brown marks medially. Labium dark brown. Sternum dark brown, with dark radiating lines. Abdomen slightly elongated, dark brown. Legs light brown; measurements: I 7.37 (1.98, 0.22, 2.25, 2.08, 0.84), II 5.26 (1.44, 0.21, 1.60, 1.41, 0.60), III 3.96 (1.13, 0.20, 1.15, 0.99, 0.49), IV 6.05 (1.62, 0.21, 1.90, 1.60, 0.72). Palp (Fig. 27A–D): femur slender, four times longer than patella; patella not swollen; tibia not swollen, half length of femur; cymbium with distal protrusion, length ratio of dorsal elongation and cymbium 1.39; bulb pale yellow; pyriform with embolus and conductor arising distally; embolus slightly bent, 1/3 width of and similar length as bulb, conductor basally connected with embolus, thin, short, needle-like, 1/5 embolus length.

**Female** (paratype). Similar to male in colouration and general features but slightly larger (Fig. 28D, E). Measurements: total length 1.70; carapace 0.64 long, 0.56 wide; abdomen 0.99 long, 0.86 wide. Leg measurements: I 5.24 (1.34, 0.20, 1.56, 1.39, 0.75), II 4.04 (1.06, 0.20, 1.20, 0.99, 0.59), III 3.29 (0.86, 0.20, 0.92, 0.82, 0.49), IV 4.81 (1.21, 0.20, 1.47, 1.23, 0.70). Epigastric area (Fig. 28B): dark brown patch, medially with horizontal pale brown slit. Endogyne (Fig. 28A) with a pair of slight horizontally angled elongated spermathecae, tip pointed upright, ratio of the width of spermatheca to the interdistance of spermathecae 1:4.

##### Distribution.

Known only from the type locality (Thailand; Fig. 54).

#### Merizocera putao

Taxon classificationAnimaliaAraneaePsilodercidae

Li sp. nov.

746FA75C-B0A8-5E18-8A50-58B2655407C0

http://zoobank.org/0438A332-A054-4D27-8830-EDED85A7B8DD

**Figure 29.**
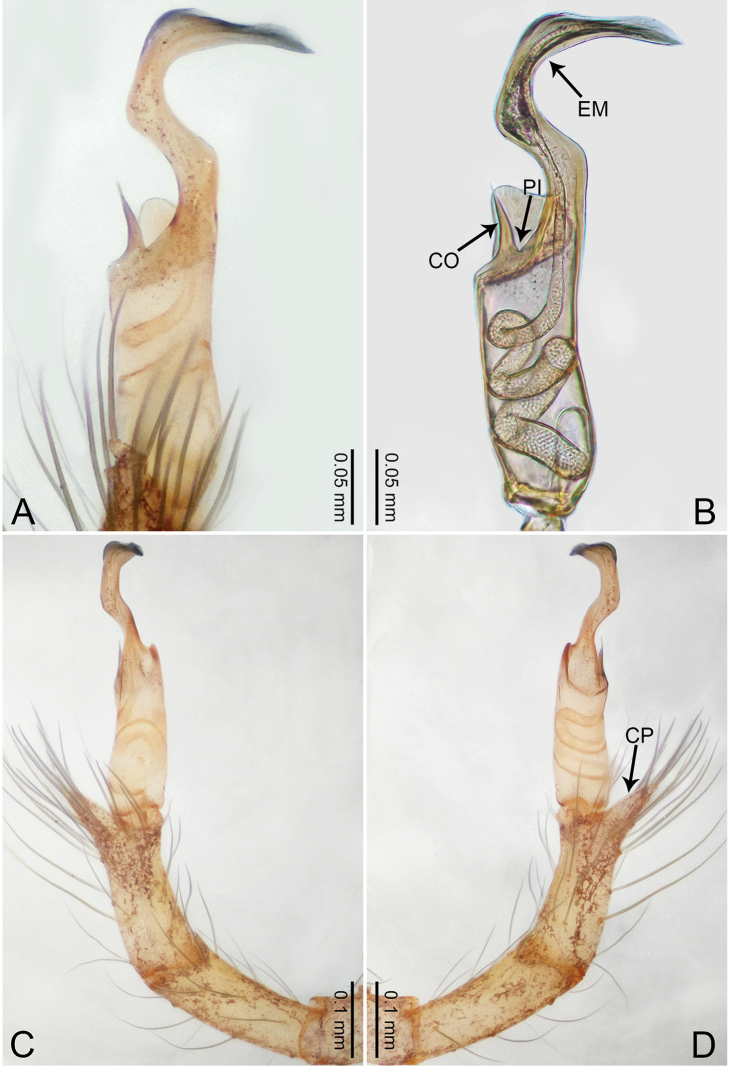
*Merizocera putao* sp. nov., holotype male. **A** Bulb, dorsal view **B** bulb, dorsal view **C** palp, prolateral view **D** palp, retrolateral view. Abbreviations: CO = conductor, CP = cymbial protrusion, EM = embolus, PI = pit.

**Figure 30.**
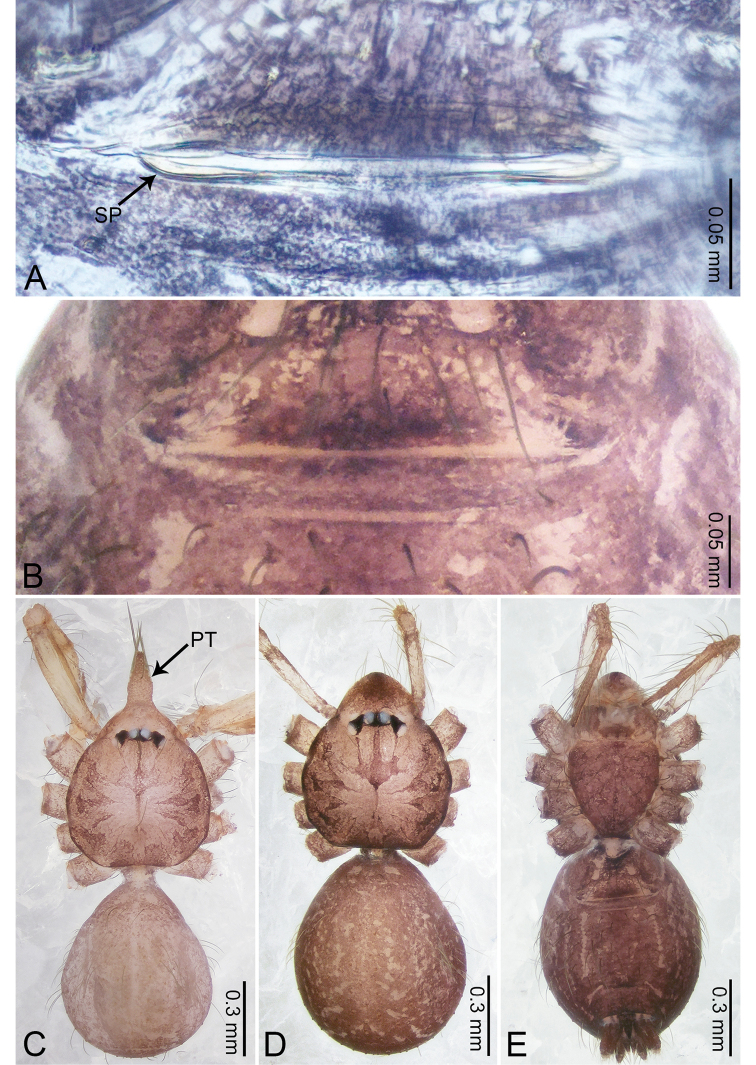
*Merizocera putao* sp. nov., holotype male and paratype female. **A** Endogyne, dorsal view **B** female epigastric area, ventral view **C** male habitus, dorsal view **D** female habitus, dorsal view **E** female habitus, ventral view. Abbreviations: PT = clypeal protrusion, SP = spermatheca.

Figures 29 , 30 , 53

##### Type material.

***Holotype***: male (IZCAS), Roadside between Upper Shankhaung Village and Wasadum (27°27.38'N, 97°13.65'E, elevation 1396 m), Putao, **Kachin State**, **Myanmar**, 11 December 2016, J. Wu leg. ***Paratype***: 1 female (IZCAS), same data as holotype.

##### Etymology.

The specific name refers to the type locality; noun in apposition.

##### Diagnosis.

Diagnosis features of males and females are discussed in *M. kachin* sp. nov. Bulb with a distinct pit and hooked embolus (Fig. 29B). Clypeal protrusion present in males (Fig. 30C). Females with elongated horizontal spermathecae with globose tips (Fig. 30A).

##### Description.

**Male** (holotype). Total length 1.60; carapace 0.83 long, 0.58 wide; abdomen 0.75 long, 0.61 wide. Carapace circular, brownish, with dark brown radiating marks and dark brown median line (Fig. 30C). Fovea shallow. Thoracic region distinctly elevated medially. Clypeus brownish, with large protrusion provided with long setae. Labium dark brown. Sternum dark brown, with dark radiating lines. Abdomen ovoid, brown. Legs light brown; measurements: I 7.61 (2.23, 0.24, 2.25, 2.00, 0.89), II 5.46 (1.48, 0.22, 1.64, 1.42, 0.70), III 4.17 (1.10, 0.21, 1.15, 1.15, 0.56), IV 5.84 (1.48, 0.22, 1.74, 1.52, 0.88). Palp (Fig. 29A–D): femur slender, four times longer than patella; patella not swollen; tibia not swollen; cymbium with distal protrusion, length ratio of dorsal elongation and cymbium 0.39; bulb pale yellow, elongated pyriform with embolus and conductor arising distally, presence of distinct pit resulting from the basal connection of embolus and conductor; embolus hooked, similar in length with tegular, with pointed tip, width of anterior horizontal hooked part slightly longer than the width of bulb; conductor upright and needle-like, 1/3 length of embolus.

**Female** (paratype). Similar to male in colouration and general features but slightly larger (Fig. 30D, E). Measurements: total length 1.42; carapace 0.67 long, 0.56 wide; abdomen 0.75 long, 0.63 wide. Leg measurements: I 4.68 (1.19, 0.21, 1.42, 1.19, 0.67), II missing, III 3.17 (0.83, 0.20, 0.85, 0.79, 0.50), IV 4.50 (1.11, 0.20, 1.38, 1.09, 0.72). Epigastric area (Fig. 30B) dark brown semi-circular patch, medially with horizontal pale brown slit. Endogyne (Fig. 30A) with a pair of elongated horizontal spermathecae with blunt tips.

##### Distribution.

Known only from the type locality (Myanmar; Fig. 53).

#### Merizocera ranong

Taxon classificationAnimaliaAraneaePsilodercidae

Li sp. nov.

E2CAE1D4-594C-55E8-8EA0-C1E9A630887A

http://zoobank.org/A1FBCDBB-C979-4A5B-9156-61C7ABB01FC1

**Figure 31.**
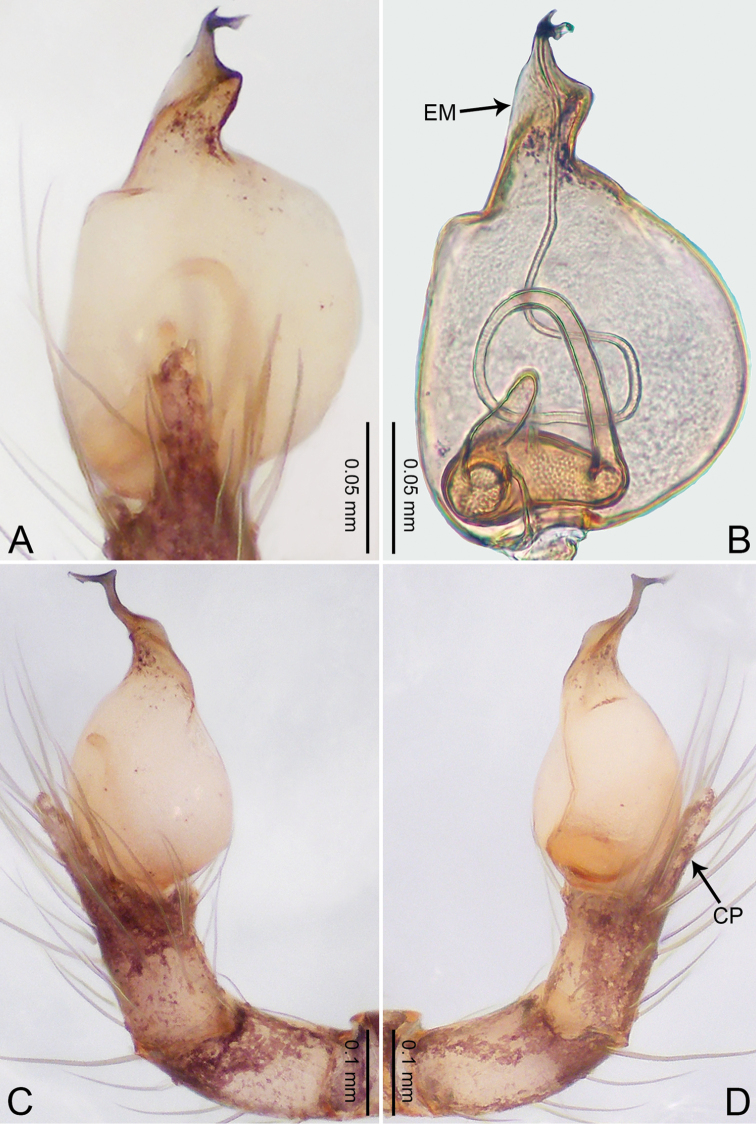
*Merizocera ranong* sp. nov., holotype male. **A** Bulb, dorsal view **B** bulb, dorsal view **C** palp, prolateral view **D** palp, retrolateral view. Abbreviations: CP = cymbial protrusion, EM = embolus.

**Figure 32.**
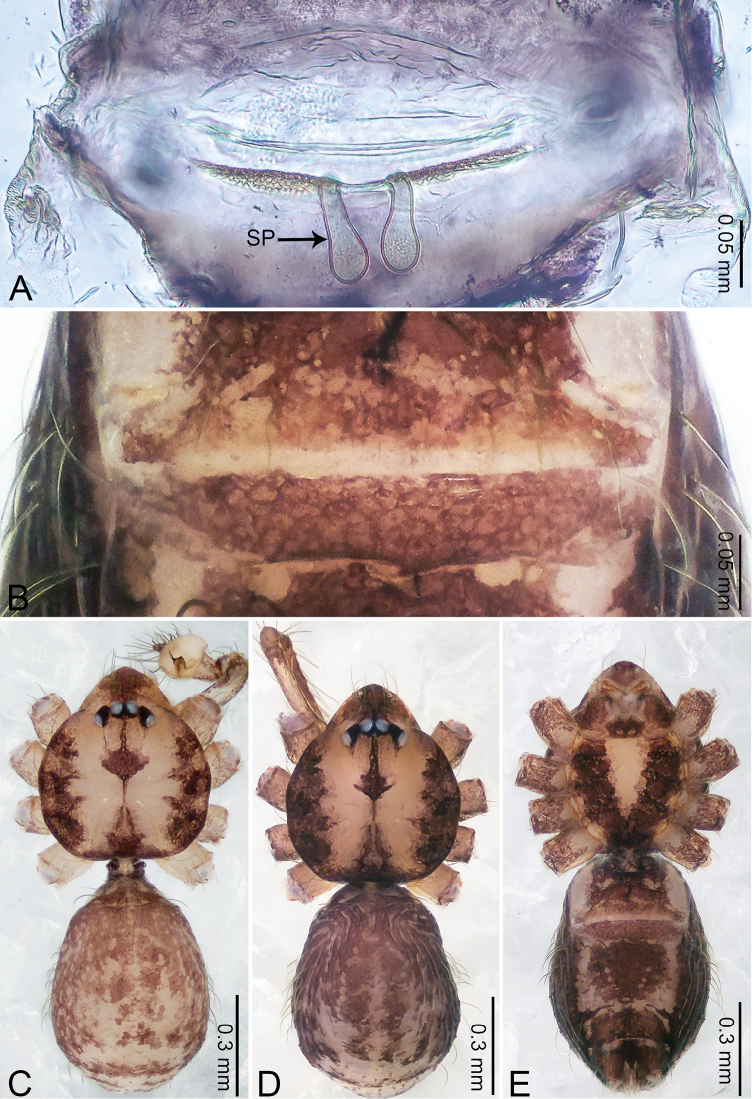
*Merizocera ranong* sp. nov., holotype male and paratype female. **A** Endogyne, dorsal view **B** female epigastric area, ventral view **C** male habitus, dorsal view **D** female habitus, dorsal view **E** female habitus, ventral view. Abbreviation: SP = spermatheca.

Figures 31 , 32 , 54

##### Type material.

***Holotype***: male (IZCAS), forest of Suk Sum Ran Village (9°28.80'N, 98°30.56'E, elevation 46 m), Kapoe District, **Ranong**, **Thailand**, 28 October 2014, P. Wongprom leg. ***Paratype***: 1 female (IZCAS), same data as holotype.

##### Etymology.

The specific name refers to the type locality; noun in apposition.

##### Diagnosis.

Diagnosis features of males and females are discussed in *M. betong* sp. nov. Males with a crinkly embolus, lamina-like embolus tip, and swollen pyriform bulb (Fig. 31B). Females with a pair of posteriorly directed tubular spermathecae (Fig. 32A).

##### Description.

**Male** (holotype). Total length 1.28; carapace 0.59 long, 0.52 wide; abdomen 0.64 long, 0.49 wide. Carapace circular, brownish, with dark brown marks laterally and dark brown median stripe on anterior half (Fig. 32C). Fovea shallow. Thoracic region distinctly elevated medially. Clypeus brownish, with dark brown marks medially. Labium dark brown. Sternum dark brown but lighter medially. Abdomen ovoid, brownish, with darker brown marks dorsally and ventrally. Legs light brown; measurements: I 6.19 (1.70, 0.21, 1.90, 1.62, 0.76), II 4.46 (1.20, 0.20, 1.33, 1.13, 0.61), III 3.40 (0.92, 0.19, 1.00, 0.84, 0.45), IV 5.05 (1.34, 0.20, 1.55, 1.27, 0.69). Palp (Fig. 31A–D): femur slender, thrice longer than patella; patella not swollen; tibia not swollen; cymbium with distal protrusion, length ratio of dorsal elongation and cymbium 0.90; bulb pale yellow, swollen pyriform with embolus arising distally; embolus with crinkly stalk and lamina-like at the tip, stalk 1/3 length of bulb; conductor absent.

**Female** (paratype). Similar to male in colouration and general features but slightly larger (Fig. 32D, E). Measurements: total length 1.28; carapace 0.62 long, 0.54 wide; abdomen 0.66 long, 0.51 wide. Leg measurements: I 5.19 (1.36, 0.21, 1.56, 1.33, 0.73), II 4.02 (1.06, 0.20, 1.15, 1.01, 0.60), III 3.25 (0.85, 0.19, 0.89, 0.83, 0.49), IV 4.75 (1.19, 0.20, 1.45, 1.20, 0.71). Epigastric area (Fig. 32B): dark brown elliptical patch, medially with horizontal pale brown slit. Endogyne (Fig. 32A) with a pair of posteriorly directed tubular spermathecae, gradually enlarged posteriorly.

##### Distribution.

Known only from the type locality (Thailand; Fig. 54).

#### Merizocera ratnapura

Taxon classificationAnimaliaAraneaePsilodercidae

Li sp. nov.

0BD3213E-0F7D-5160-B006-845D731B5F6F

http://zoobank.org/4F52770D-6E94-42C0-A1EE-CC0B32CEB28C

**Figure 33.**
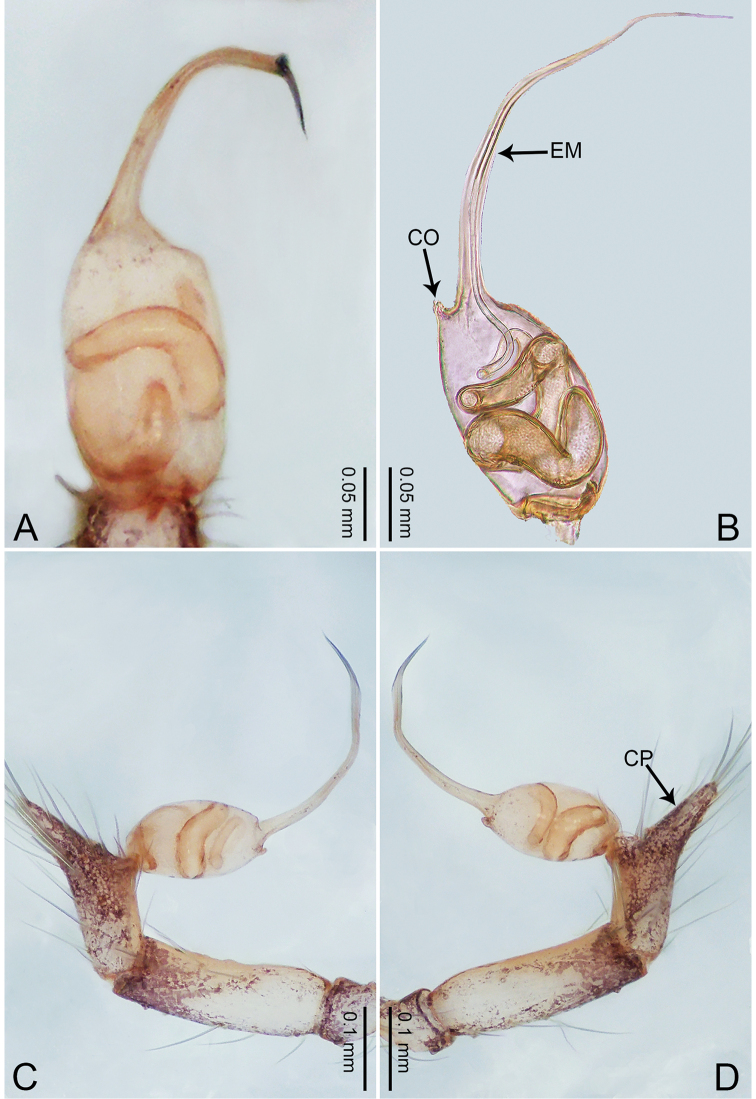
*Merizocera ratnapura* sp. nov., holotype male. **A** Bulb, dorsal view **B** bulb, retrolateral view **C** palp, prolateral view **D** palp, retrolateral view. Abbreviations: CO = conductor, CP = cymbial protrusion, EM = embolus.

**Figure 34.**
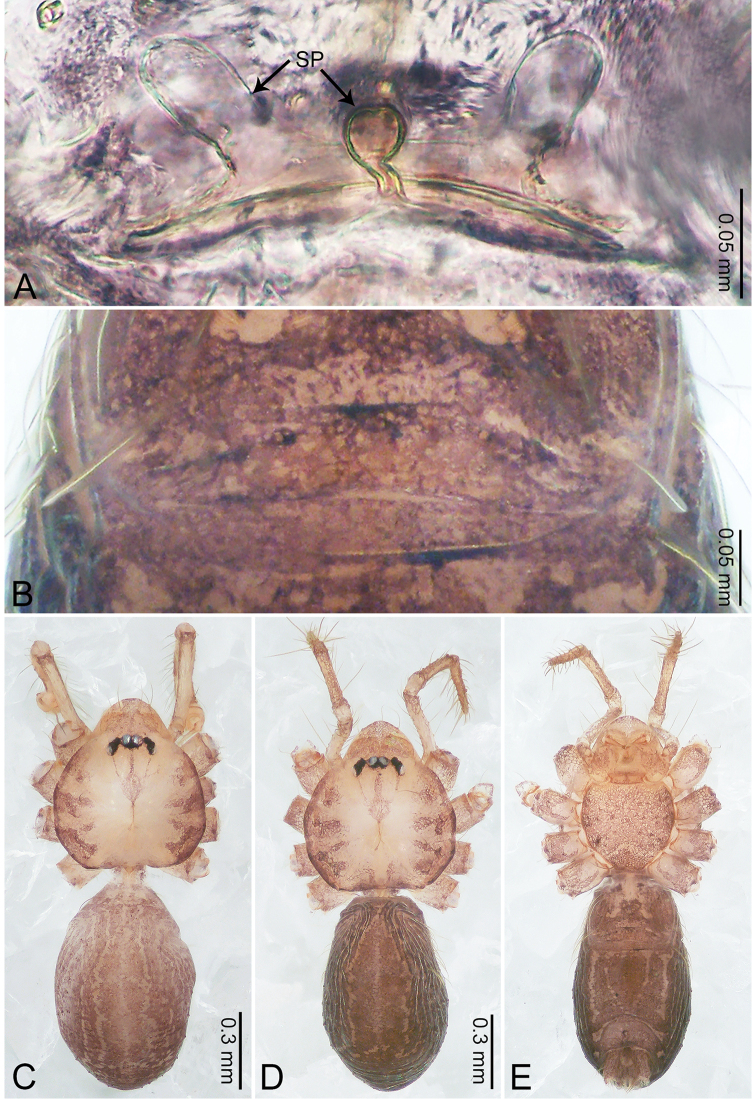
*Merizocera ratnapura* sp. nov., holotype male and paratype female. **A** Endogyne, dorsal view **B** female epigastric area, ventral view **C** male habitus, dorsal view **D** female habitus, dorsal view **E** female habitus, ventral view. Abbreviation: SP = spermatheca.

Figures 33 , 34 , 52

##### Type material.

***Holotype***: male (IZCAS), Isthripura Cave (6°49.90'N, 80°22.46'E, elevation 268 m), Batatota Village, Adam’s Peak Area, Ekneligoda Town, Kuruwita, Ratnapura District, **Sabaragamuwa**, **Sri Lanka**, 28 September 2014, S. Kosala leg. ***Paratype***: 1 female (IZCAS), same data as holotype.

##### Etymology.

The specific name refers to the type locality; noun in apposition.

##### Diagnosis.

Diagnosis features of males and females are discussed in *M. phuket* sp. nov. Males with a thick and short conductor and a gradually tapering embolus (Fig. 33B). Females with a pair of tubular spermathecae laterally and stalked spermathecae medially (Fig. 34A).

##### Description.

**Male** (holotype). Total length 1.48; carapace 0.66 long, 0.59 wide; abdomen 0.80 long, 0.55 wide. Carapace circular, brownish, with dark brown marks laterally and brown median line on anterior half (Fig. 34C). Fovea shallow. Thoracic region distinctly elevated medially. Clypeus and labium brown. Sternum dark brown. Abdomen slightly elongated, dark brown. Legs light brown; measurements: I, III and IV missing, II 5.74 (1.66, 0.24, 1.76, 1.41, 0.67). Palp (Fig. 33A–D): femur slender, five times longer than patella; patella not swollen; tibia not swollen; tibia not swollen, 1/2 femur length; cymbium with distal protrusion, 1/3 femur length, length ratio of dorsal elongation and cymbium: 0.91; bulb pale yellow, pyriform with embolus and conductor arising distally; embolus elongated and bent, 2.5 times longer than bulb, gradually tapering and darkened at the tip; conductor basally connected with embolus.

**Female** (paratype). Similar to male in colouration and general features but slightly larger (Fig. 34D, E). Measurements: total length 1.48; carapace 0.70 long, 0.62 wide; abdomen 0.76 long, 0.51 wide. Leg measurements: I 9.38 (2.59, 0.24, 2.97, 2.45, 1.13), II 6.75 (1.92, 0.24, 2.10, 1.66, 0.83), III 5.10 (1.47, 0.23, 1.53, 1.24, 0.63), IV 7.36 (2.08, 0.23, 2.35, 1.82, 0.88). Epigastric area (Fig. 34B): dark brown elliptical patch. Endogyne (Fig. 34A) with a pair of widely tubular spermathecae, medially with stalked spermathecae bearing globose end.

##### Distribution.

Known only from the type locality (Sri Lanka; Fig. 52).

#### Merizocera salawa

Taxon classificationAnimaliaAraneaePsilodercidae

Li sp. nov.

C0C443C9-F732-5641-ACBE-C92557284972

http://zoobank.org/C706A307-0868-44DA-AFAB-57E83D0924FC

**Figure 35.**
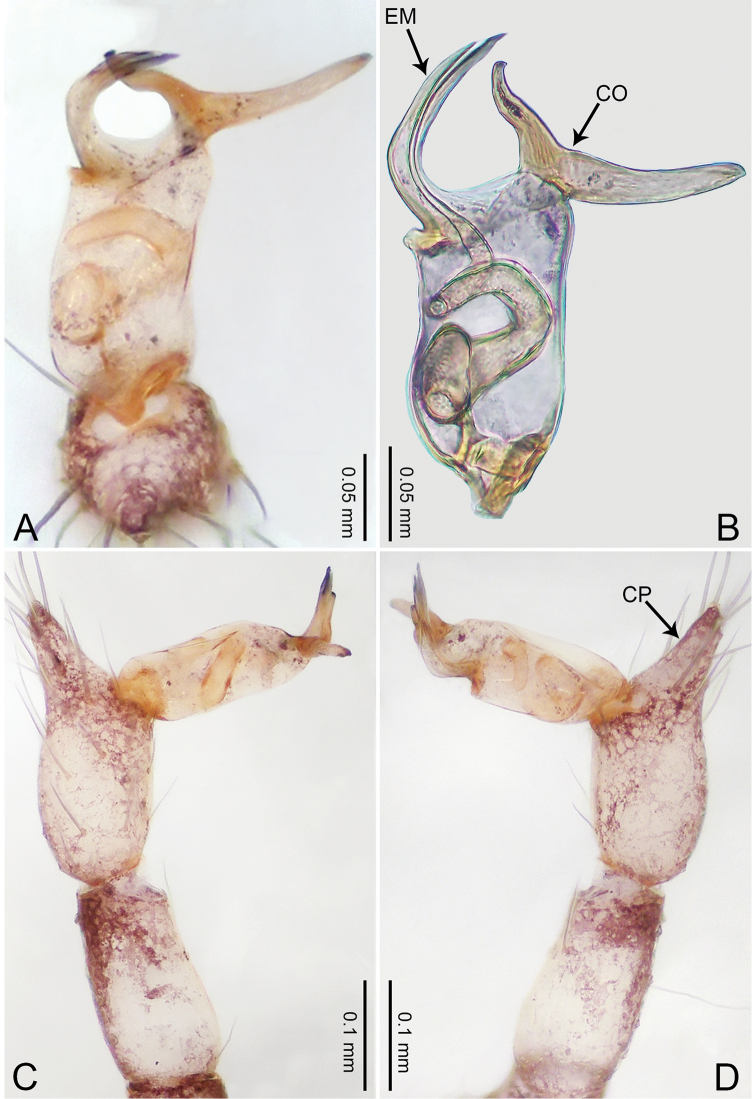
*Merizocera salawa* sp. nov., holotype male. **A** Bulb, dorsal view **B** bulb, dorsal view, embolus and conductor distorted **C** palp, prolateral view **D** palp, retrolateral view. Abbreviations: CO = conductor, CP = cymbial protrusion, EM = embolus.

**Figure 36.**
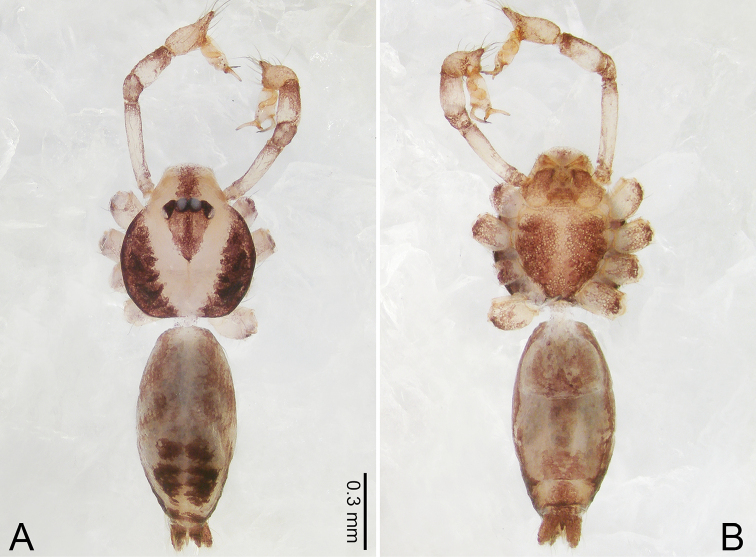
*Merizocera salawa* sp. nov., holotype male. **A** Habitus, dorsal view **B** habitus, ventral view.

Figures 35 , 36 , 52

##### Type material.

***Holotype***: male (IZCAS), Lenawara Lena (= cave) (6°56.77'N, 80°6.76'E, elevation 66 m), Salawa Village, Avissawella Town, Maniyangama, Colombo District, **Western Province**, **Sri Lanka**, 25 September 2014, S. Kosala leg.

##### Etymology.

The specific name refers to the type locality; noun in apposition.

##### Diagnosis.

Diagnosis features of males are discussed in *M. mandai* sp. nov. Bulb with a widely separated bifurcate conductor, and a curved embolus (Fig. 35B).

##### Description.

**Male** (holotype). Total length 1.52; carapace 0.61 long, 0.54 wide; abdomen 0.88 long, 0.41 wide. Carapace circular, brownish, with dark brown marks laterally and dark brown median stripe on anterior half (Fig. 36C). Fovea shallow. Thoracic region distinctly elevated medially. Clypeus brownish, with dark brown marks medially. Labium dark brown. Sternum dark brown but brownish medially. Abdomen elongated, dark grey, with dark brown marks dorsally and ventrally. Legs missing. Palp (Fig. 35A–D): femur slender, four times longer than patella; patella not swollen; tibia not swollen, 1/2 femur length; cymbium with distal protrusion, 1/2 femur length, length ratio of dorsal elongation and cymbium: 0.63; bulb pale yellow, pyriform with embolus and conductor emerging distally; embolus arising laterally, bent, with consistent width; conductor bifurcate, arising laterally, similar width as embolus, resembles a widely open crescent-shape.

**Female.** Unknown.

##### Distribution.

Known only from the type locality (Sri Lanka; Fig. 52).

#### Merizocera tak

Taxon classificationAnimaliaAraneaePsilodercidae

Li sp. nov.

945E8B4F-6652-5473-92D5-B9A30CBA7EB5

http://zoobank.org/8E300899-E573-4D65-8550-B531D5EB593C

Figures 37 , 54

##### Type material.

***Holotype***: female (IZCAS), Mae Klong Noi Subdistrict (16°14.64'N, 98°59.91'E, elevation 1228 m), Umphang District, **Tak**, **Thailand**, 17 November 2016, P. Wongprom leg.

##### Etymology.

The specific name refers to the type locality; noun in apposition.

##### Diagnosis.

**Figure 37.**
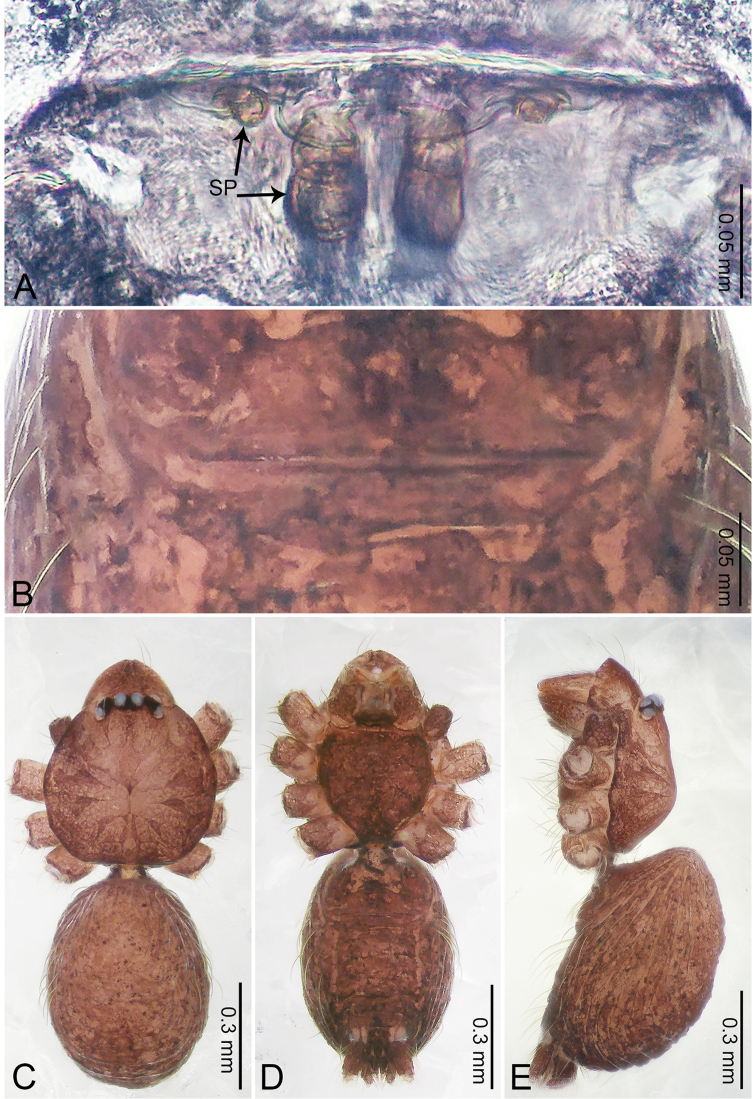
*Merizocera tak* sp. nov., holotype female. **A** Endogyne, dorsal view **B** female epigastric area, ventral view **C** female habitus, dorsal view **D** female habitus, ventral view **E** female habitus, lateral view. Abbreviation: SP = spermatheca.

Females can be distinguished from all congeners by the presence of two pairs of globose spermathecae, the median pair resembling the figure ‘8’ (Fig. 37A).

##### Description.

**Female** (holotype). Total length 1.21; carapace 0.58 long, 0.49 wide; abdomen 0.61 long, 0.47 wide. Carapace circular, brown, with dark brown radiating marks (Fig. 37C). Fovea shallow. Thoracic region distinctly elevated medially. Clypeus, labium, and sternum dark brown. Abdomen ovoid, dark brown (Fig. 37B). Legs light brown; measurements: I 3.47 (0.84, 0.19, 1.03, 0.85, 0.56), II 2.93 (0.73, 0.18, 0.84, 0.68, 0.50), III 2.41 (0.59, 0.16, 0.65, 0.58, 0.43), IV 3.48 (0.85, 0.19, 1.00, 0.85, 0.59). Epigastric area (Fig. 37B): dark brown semi-circular patch, medially with a slit. Endogyne (Fig. 37A) with two pairs of globose spermathecae, median pair made up of two overlying globose spermathecae resembling the figure ‘8’, lateral pairs 1/4 size of median pair.

**Male.** Unknown.

##### Distribution.

Known only from the type locality (Thailand; Fig. 54).

#### Merizocera tanintharyi

Taxon classificationAnimaliaAraneaePsilodercidae

Li sp. nov.

92B0A56F-C1B0-5146-9204-E307F32C7C24

http://zoobank.org/7ECF3725-D050-4174-9B28-FB209AD7AB8B

**Figure 38.**
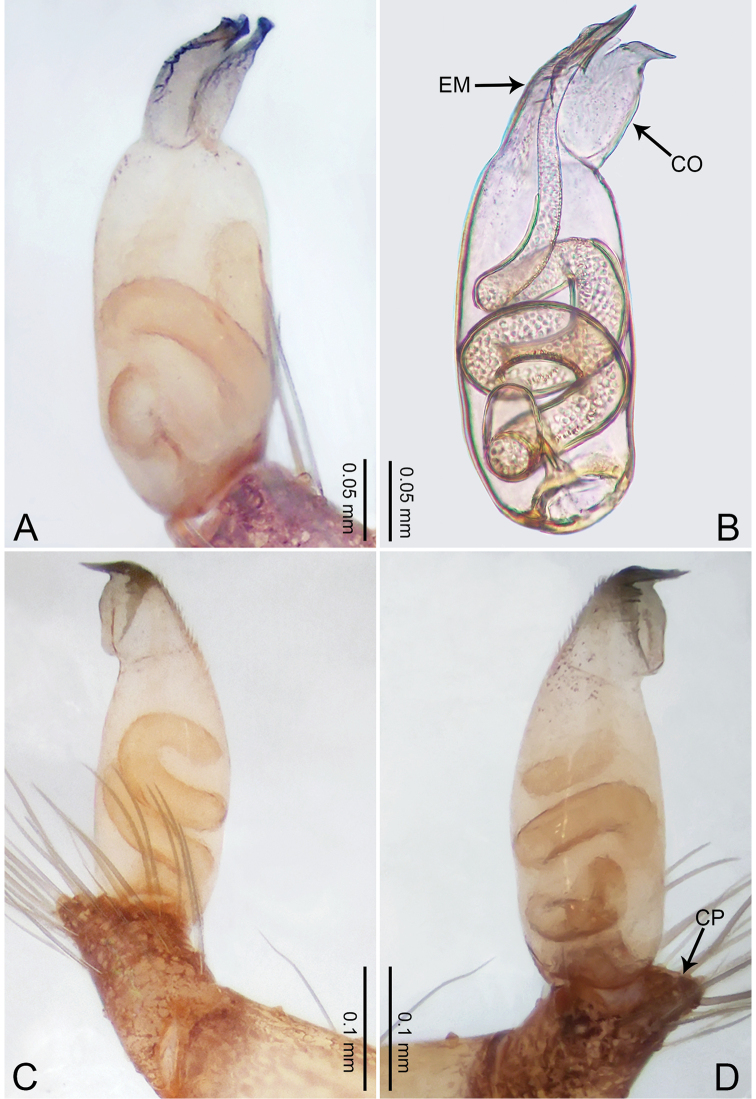
*Merizocera tanintharyi* sp. nov., holotype male. **A** Bulb, dorsal view **B** bulb, dorsal view, embolus and conductor distorted **C** palp, prolateral view **D** palp, retrolateral view. Abbreviations: CO = conductor, CP = cymbial protrusion, EM = embolus.

**Figure 39.**
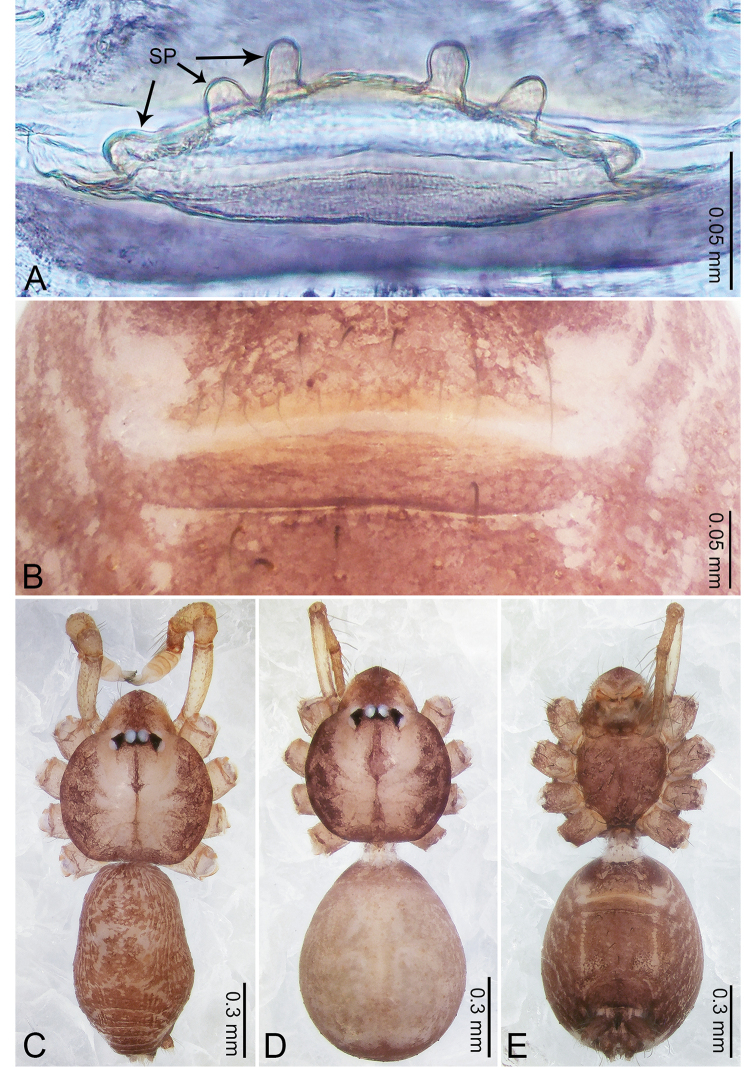
*Merizocera tanintharyi* sp. nov., holotype male and paratype female. **A** Endogyne, dorsal view **B** female epigastric area, ventral view **C** male habitus, dorsal view **D** female habitus, dorsal view **E** female habitus, ventral view. Abbreviation: SP = spermatheca.

Figures 38 , 39 , 54

##### Type material.

***Holotype***: male (IZCAS), Tanintharyi Nature Reserve (14°44.12'N, 98°11.55'E, elevation 307 m), **Myanmar**, 24 October 2017, Z. Chen leg. ***Paratypes***: 1 male and 3 females (IZCAS), same data as holotype.

##### Etymology.

The specific name refers to the type locality; noun in apposition.

##### Diagnosis.

Diagnosis features of males and females are discussed in *M. mainling* sp. nov. Recognised by a wide and appendage-like conductor as long as the embolus (Fig. 38B). It can be distinguished from the *Psiloderces leucopygius* group by an inconspicuous cymbial protrusion and a distinct conductor (vs. distinct or short cymbial protrusion and an inconspicuous conductor in the *leucopygius* group).

##### Description.

**Male** (holotype). Total length 1.76; carapace 0.85 long, 0.74 wide; abdomen 0.95 long, 0.59 wide. Carapace circular, brownish, with dark brown marks laterally and dark brown median line (Fig. 39C). Fovea shallow. Thoracic region distinctly elevated medially. Clypeus, labium and sternum dark brown. Abdomen slightly elongated, dark brown. Legs light brown; measurements: I 9.11 (2.66, 0.31, 2.97, 2.25, 0.92), II 7.14 (1.90, 0.27, 2.10, 1.98, 0.89), III 5.50 (1.53, 0.26, 1.64, 1.44, 0.63), IV 8.18 (2.20, 0.29, 2.45, 2.28, 0.96). Palp (Fig. 38A–D): femur slender, 1/3 patella length; patella not swollen; tibia not swollen, 1/2 femur length; cymbium with distal protrusion, length ratio of dorsal elongation and cymbium 0.40; bulb pale yellow, pyriform with embolus and conductor emerging distally, attached but separated at the tip; embolus pointed and darken at the tip; conductor gradually thinner at the tip.

**Female** (paratype). Similar to the male in colouration and general features but slightly larger (Fig. 39D, E). Measurements: total length 1.66; carapace 0.75 long, 0.62 wide; abdomen 0.91 long, 0.71 wide. Leg measurements: I 6.12 (1.55, 0.25, 1.82, 1.60, 0.90), II 4.80 (1.23, 0.23, 1.38, 1.24, 0.72), III 3.90 (1.03, 0.22, 1.09, 0.99, 0.57), IV missing. Epigastric area (Fig. 39B): dark brown elliptical patch, medially with pale yellow slit. Endogyne (Fig. 39A) with three pairs of short tubular spermathecae, lateral pairs relatively widely separated, median two pairs attached closely together.

##### Distribution.

Known only from the type locality (Myanmar; Fig. 54).

#### Merizocera tengchong

Taxon classificationAnimaliaAraneaePsilodercidae

Li sp. nov.

D79BD504-7236-5992-BCB2-BE7C1028717B

http://zoobank.org/5BF76A00-C9E3-4B05-93BF-06E37778DD53

**Figure 40.**
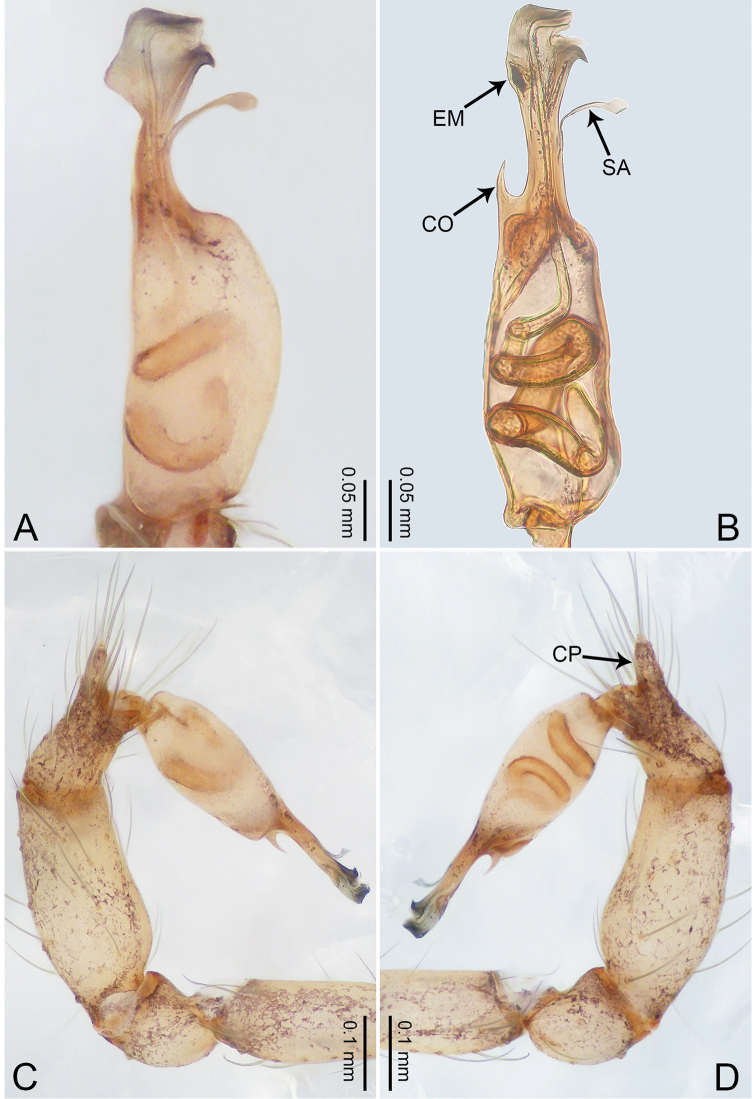
*Merizocera tengchong* sp. nov., holotype male. **A** Bulb, dorsal view **B** bulb, retrolateral view **C** palp, prolateral view **D** palp, retrolateral view. Abbreviations: CO = conductor, CP = cymbial protrusion, EM = embolus, SA = stalked apophysis.

**Figure 41.**
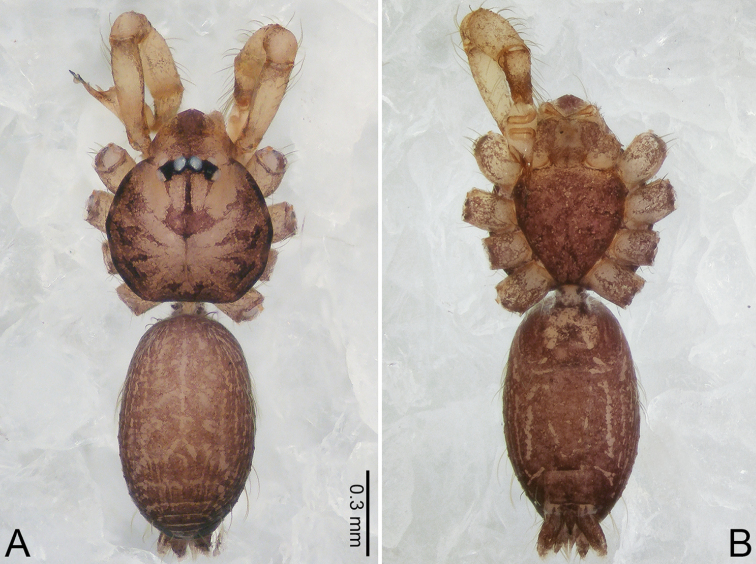
*Merizocera tengchong* sp. nov., holotype male. **A** Habitus, dorsal view **B** habitus, ventral view.

Figures 40 , 41 , 53

##### Type material.

***Holotype***: male (IZCAS), Gaoligongshan National Nature Reserve (24°49.74'N, 98°46.06'E, elevation 2177 m), Tengchong County, Baoshan, **Yunnan**, **China**, 21–22 June 2013, Z. Zhao and J. Liu leg. ***Paratype***: 1 male (IZCAS), same data as holotype.

##### Etymology.

The specific name refers to the type locality; noun in apposition.

##### Diagnosis.

Males can be recognised from congeners by the webbed feet-like embolus with a basally attached, stalked apophysis bearing a globose tip (Fig. 40B).

##### Description.

**Male** (holotype). Total length 1.53; carapace 0.67 long, 0.59 wide; abdomen 0.81 long, 0.49 wide. Carapace circular, brown, with dark brown radiating marks (Fig. 41C). Fovea shallow. Thoracic region distinctly elevated medially. Clypeus, labium, and sternum dark brown. Abdomen slightly elongated, dark brown. Legs light brown; measurements: I 5.71 (1.50, 0.25, 1.76, 1.45, 0.75), II and III missing, IV 5.00 (1.31, 0.22, 1.53, 1.23, 0.71). Palp (Fig. 40A–D): femur slender, thrice longer than patella; patella not swollen; tibia swollen proximally; cymbium with distal protrusion, 1/3 length of femur, length ratio of dorsal elongation and cymbium 0.47; bulb light brown, elongated pyriform, with embolus and conductor arising distally; embolus webbed feet-like with darken tip, attached with thin stalked apophysis bearing globose end, embolus length similar to bulb length; conductor needle-like and slightly bent, 1/4 as long as embolus.

**Female.** Unknown.

##### Distribution.

Known only from the type locality (China; Fig. 53).

#### Merizocera thenna

Taxon classificationAnimaliaAraneaePsilodercidae

Li sp. nov.

7CF45AFB-6462-5CA0-8264-66D77796471E

http://zoobank.org/17D2C8DC-6F36-43AC-A45D-0E464F936B96

**Figure 42.**
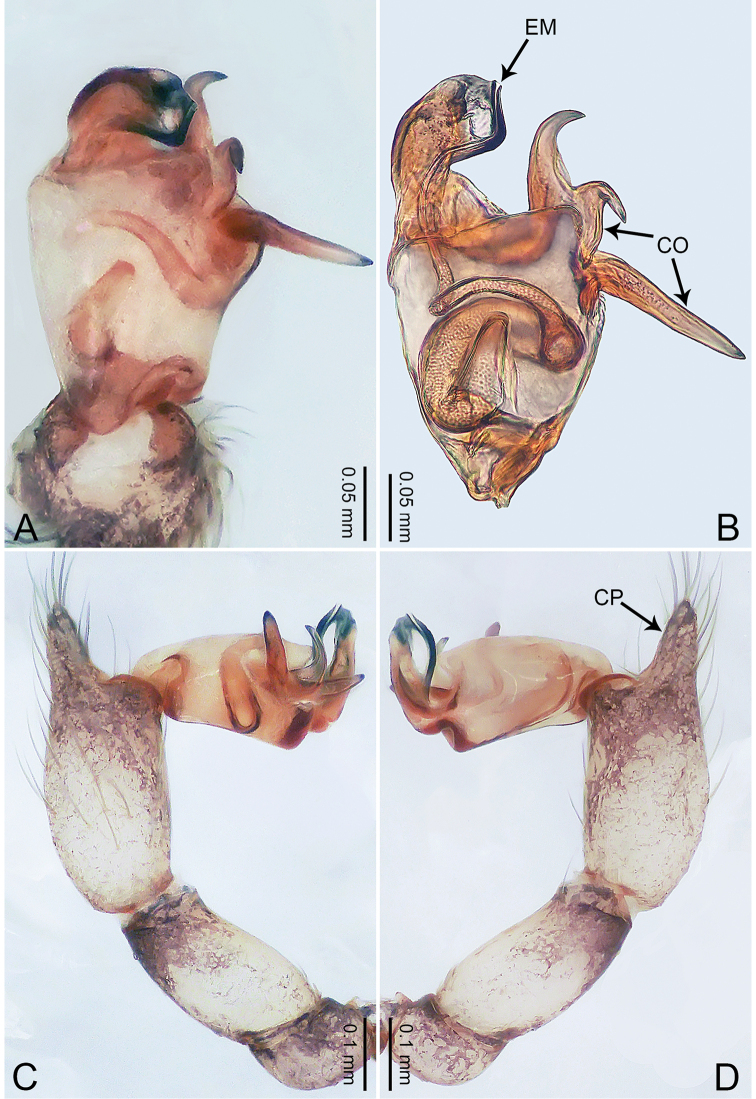
*Merizocera thenna* sp. nov., holotype male. **A** Bulb, dorsal view **B** bulb, dorsal view, embolus and conductor distorted **C** palp, prolateral view **D** palp, retrolateral view. Abbreviations: CO = conductor, CP = cymbial protrusion, EM = embolus.

**Figure 43.**
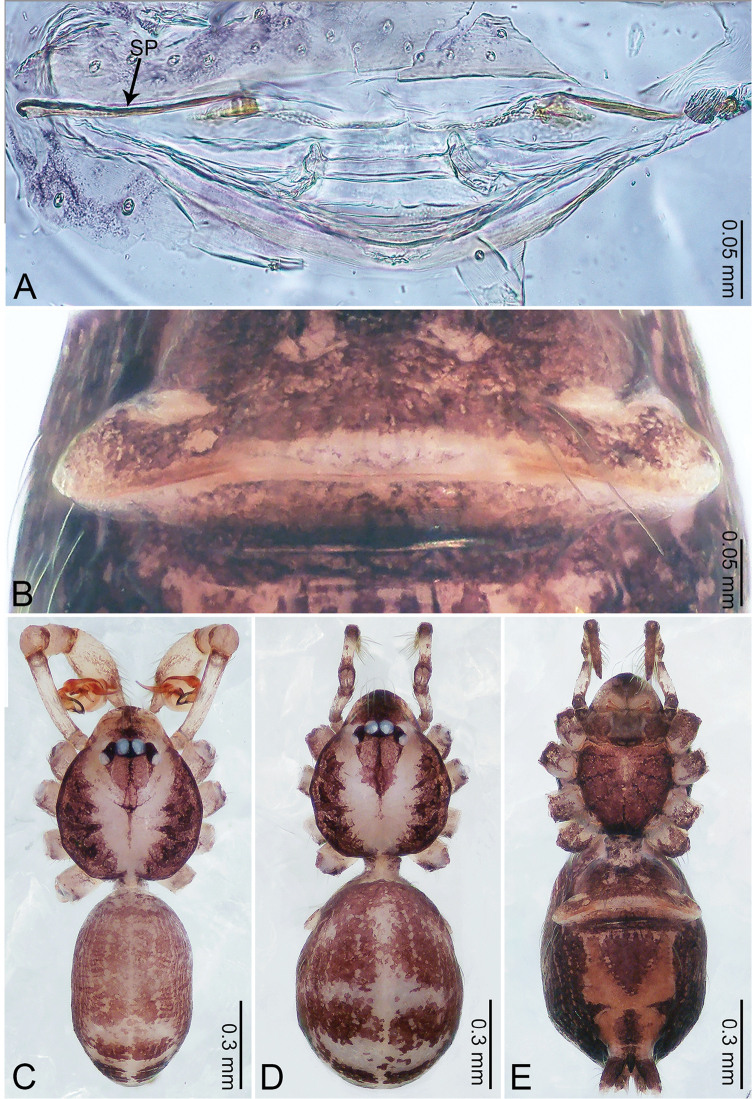
*Merizocera thenna* sp. nov., holotype male and paratype female. **A** Endogyne, dorsal view **B** female epigastric area, ventral view **C** male habitus, dorsal view **D** female habitus, dorsal view **E** female habitus, ventral view. Abbreviation: SP = spermatheca.

Figures 42 , 43 , 52

##### Type material.

***Holotype***: male (IZCAS), near the Suwargeya Cave of Archeaology place of Kuragala (6°37.45'N, 80°52.21'E, elevation 439 m), Thenna Village, Kaldoda Town, Balangoda, Ratnapura District, **Sabaragamuwa**, **Sri Lanka**, 1 October 2014, S. Kosala leg. ***Paratypes***: 2 females (IZCAS), same data as holotype.

##### Etymology.

The specific name refers to the type locality; noun in apposition.

##### Diagnosis.

Males can be recognised from all other congeners by the twisted widened embolus and a trifurcate conductor (Fig. 42B). Females can be distinguished by a pair of horizontal, slender, stalked spermathecae, both ends bearing globose distal parts (Fig. 43A).

##### Description.

**Male** (holotype). Total length 1.31; carapace 0.61 long, 0.50 wide; abdomen 0.65 long, 0.42 wide. Carapace circular, brownish, with dark brown marks laterally and dark brown median stripe on anterior half (Fig. 43C). Fovea shallow. Thoracic region distinctly elevated medially. Clypeus brownish, with dark brown marks medially. Labium dark brown. Sternum dark brown but brownish medially, with distinct dark radiating lines. Abdomen slightly elongated, brownish, with dark brown marks dorsally and ventrally. Legs light brown; measurements: I 5.33 (1.36, 0.19, 1.64, 1.41, 0.73), II 3.85 (1.00, 0.20, 1.11, 0.97, 0.57), III 3.13 (0.84, 0.17, 0.88, 0.79, 0.45), IV missing. Palp (Fig. 42A–D): femur slender, four times longer than patella; patella not swollen; tibia swollen proximally; cymbium with distal protrusion, length ratio of dorsal elongation and cymbium 0.46; bulb brown, pyriform with embolus and conductor arising distally; embolus twisted and dark at the tip, approx. half the width and length of bulb; conductor arising laterally, trifurcate, two hooked, one twice as long as the other but with pointed tip.

**Female** (paratype). Similar to male in colouration and general features but slightly larger (Fig. 43D, E). Measurements: total length 1.31; carapace 0.56 long, 0.48 wide; abdomen 0.67 long, 0.57 wide. Leg measurements: I and II missing, III 2.26 (0.58, 0.16, 0.62, 0.54, 0.36), IV 3.35 (0.82, 0.18, 1.00, 0.84, 0.51). Epigastric area (Fig. 43B): light brown elongated patch. Endogyne (Fig. 43A) with a pair of horizontally stalked spermathecae, both ends bearing globose distal ends, ratio of the width of spermatheca to the interdistance of spermathecae 1:3.5.

##### Distribution.

Known only from the type locality (Sri Lanka; Fig. 52).

#### Merizocera uva

Taxon classificationAnimaliaAraneaePsilodercidae

Li sp. nov.

331B0FA3-AE83-576C-81A0-D206DC232849

http://zoobank.org/E6D72E46-677F-4B14-AEBA-BC3FF497D2B1

Figures 44 , 52

Merizocera sp. 158: Li and Li 2018 (molecular data).

##### Type material.

***Holotype***: female (IZCAS), Udakirinda Cave 1 (6°50.24'N, 81°3.85'E, elevation 855 m), Koradogolla Village, Ella, Badulla District, **Uva**, **Sri Lanka**, 3 October 2014, S. Kosala leg. ***Paratype***: 1 female (IZCAS), same data as holotype.

##### Etymology.

The specific name refers to the type locality; noun in apposition.

##### Diagnosis.

**Figure 44.**
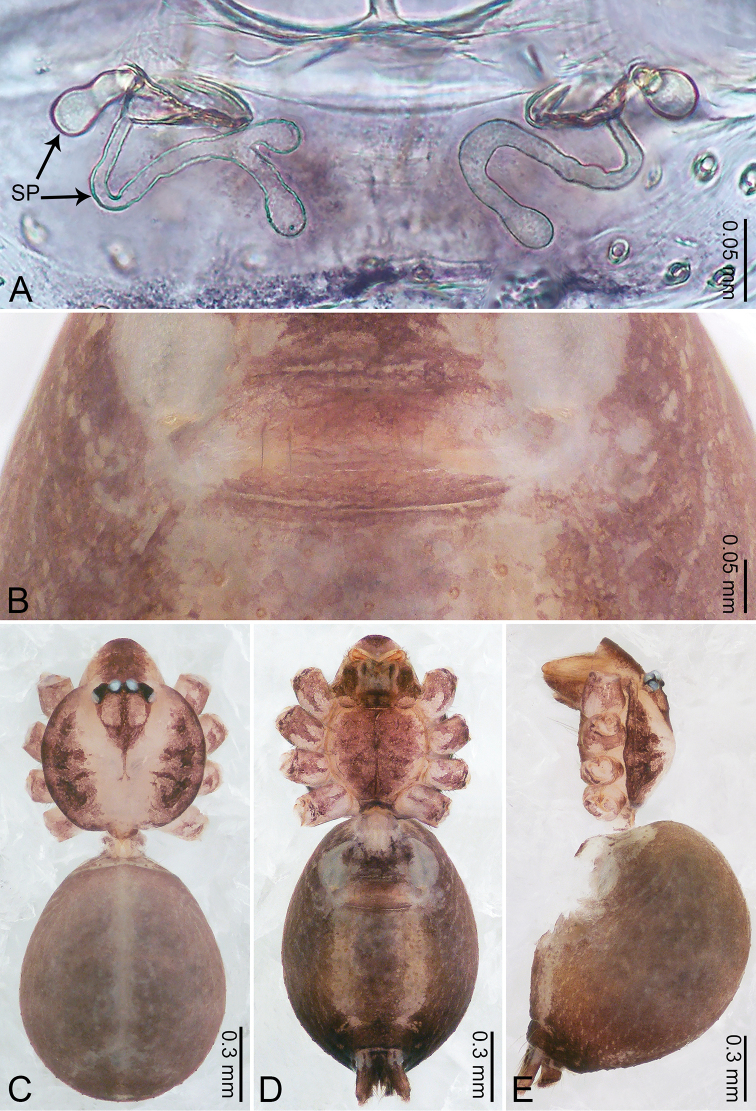
*Merizocera uva* sp. nov., holotype female. **A** Endogyne, dorsal view **B** female epigastric area, ventral view **C** female habitus, dorsal view **D** female habitus, ventral view **E** female habitus, lateral view. Abbreviation: SP = spermatheca.

Females resemble those of *M. yala* sp. nov. by having twisted stalked spermathecae but can be distinguished by having two pairs of twisted stalked spermathecae, with one end ellipsoid and the other end bifurcately globose (Fig. 44A) (vs. only globose distal ends (Fig. 49A)).

##### Description.

**Female** (holotype). Total length 1.86; carapace 0.79 long, 0.66 wide; abdomen 1.01 long, 0.84 wide. Carapace circular, brownish, with dark brown marks laterally and dark brown median stripe on anterior half (Fig. 44C). Fovea shallow. Thoracic region distinctly elevated medially. Clypeus brownish, with dark brown marks medially. Labium dark brown. Sternum dark brown but brownish laterally. Abdomen ovoid, dark grey but dark brown posteriorly and ventrally (Fig. 44B). Epigastric area (Fig. 44B): dark brown ellipsoid patch, medially with a slit.

**Male.** Unknown.

##### Distribution.

Known only from the type locality (Sri Lanka; Fig. 52).

#### Merizocera wenshan

Taxon classificationAnimaliaAraneaePsilodercidae

Li sp. nov.

7E16C9CB-2988-538A-8463-D112C0641244

http://zoobank.org/47807A72-FF91-4508-856C-F0B03DA0CB0C

**Figure 45.**
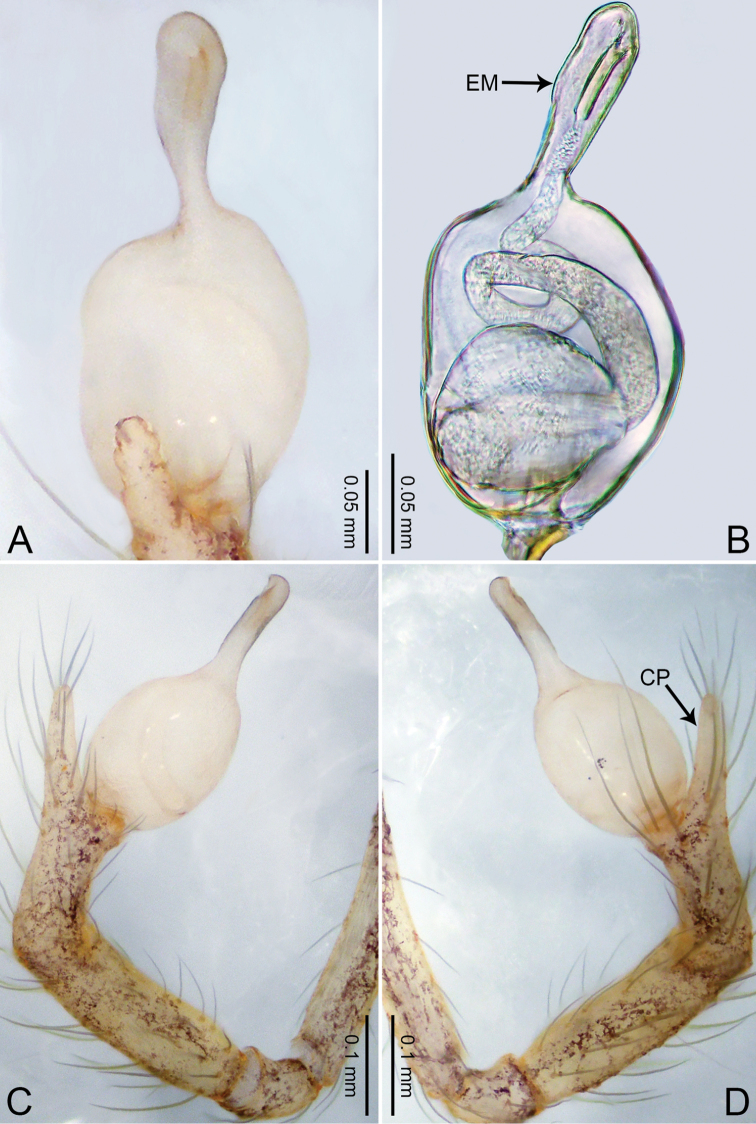
*Merizocera wenshan* sp. nov., holotype male. **A** Bulb, dorsal view **B** bulb, dorsal view **C** palp, prolateral view **D** palp, retrolateral view. Abbreviations: CP = cymbial protrusion, EM = embolus.

**Figure 46.**
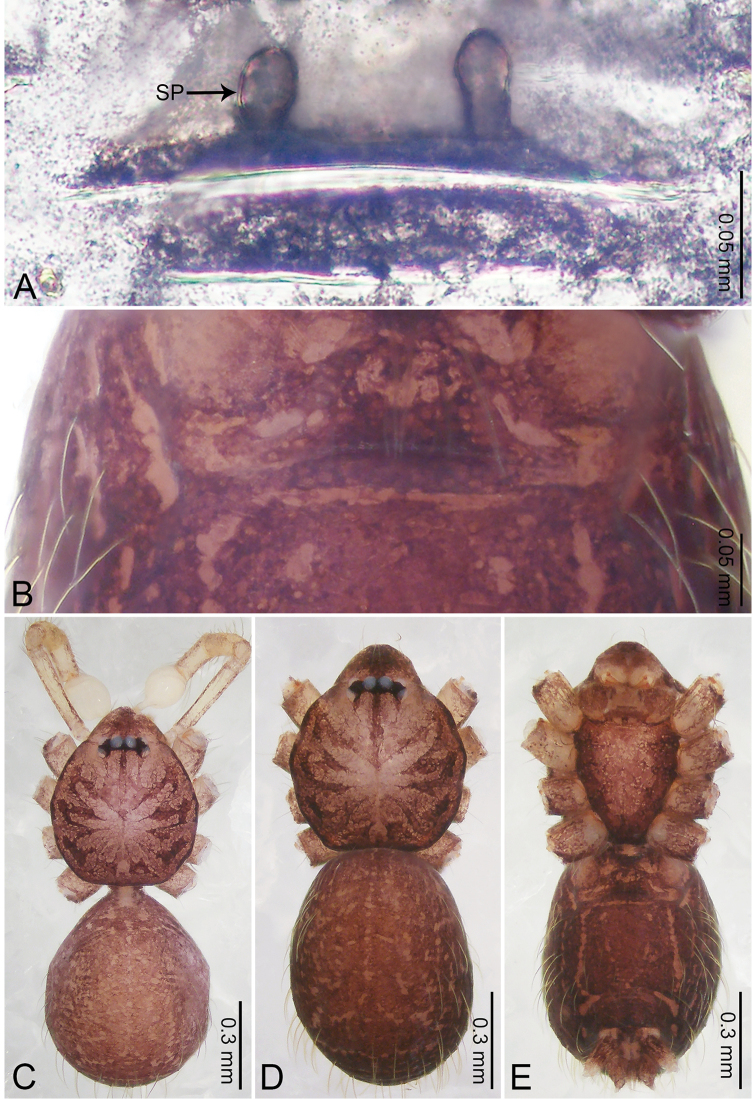
*Merizocera wenshan* sp. nov., holotype male and paratype female. **A** Endogyne, dorsal view **B** female epigastric area, ventral view **C** male habitus, dorsal view **D** female habitus, dorsal view **E** female habitus, ventral view. Abbreviation: SP = spermatheca.

Figures 45 , 46 , 53

##### Type material.

***Holotype***: male (IZCAS), near Daweishan National Nature Reserve (22°54.65'N, 103°41.78'E, elevation 2070 m), Pingbian County, Wenshan, **Yunnan**, **China**, 21 May 2015, Z. Chen and Y. Li leg. ***Paratypes***: 3 females (IZCAS), same data as holotype.

##### Etymology.

The specific name refers to the type locality; noun in apposition.

##### Diagnosis.

**Figure 47.**
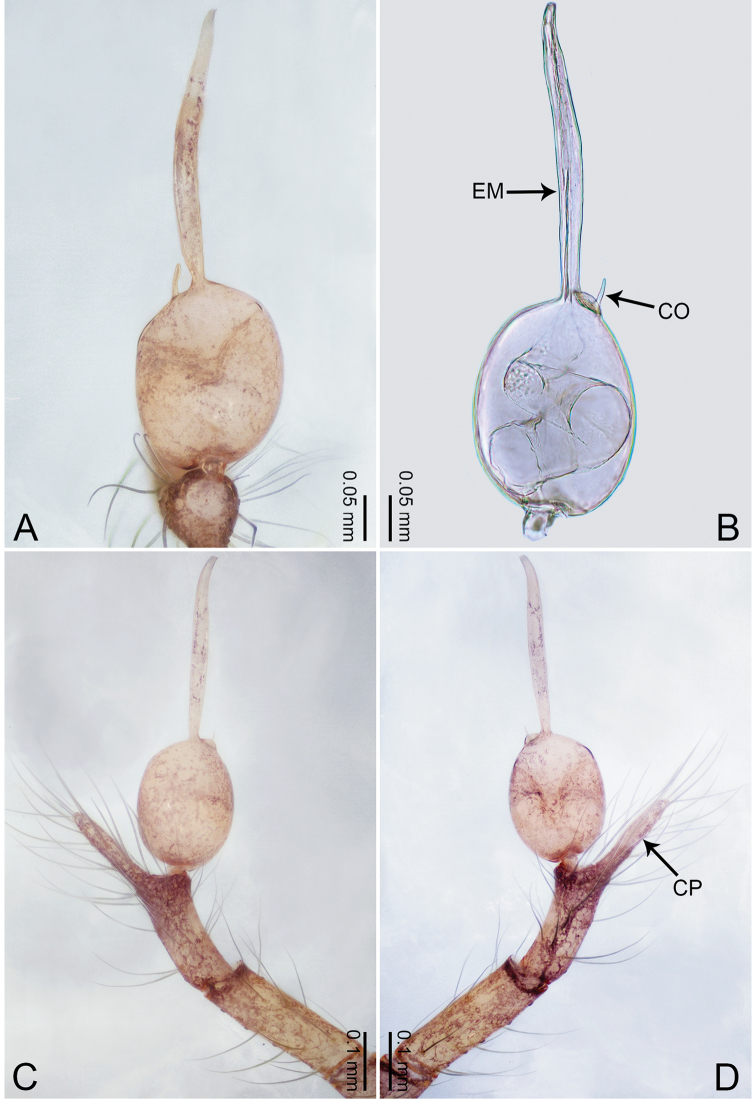
*Merizocera wui* sp. nov., holotype (**A, C, D**) and paratype (**B**) male. **A** Bulb, dorsal view **B** bulb, prolateral view **C** palp, prolateral view **D** palp, retrolateral view. Abbreviations: CO = conductor, CP = cymbial protrusion, EM = embolus.

**Figure 48.**
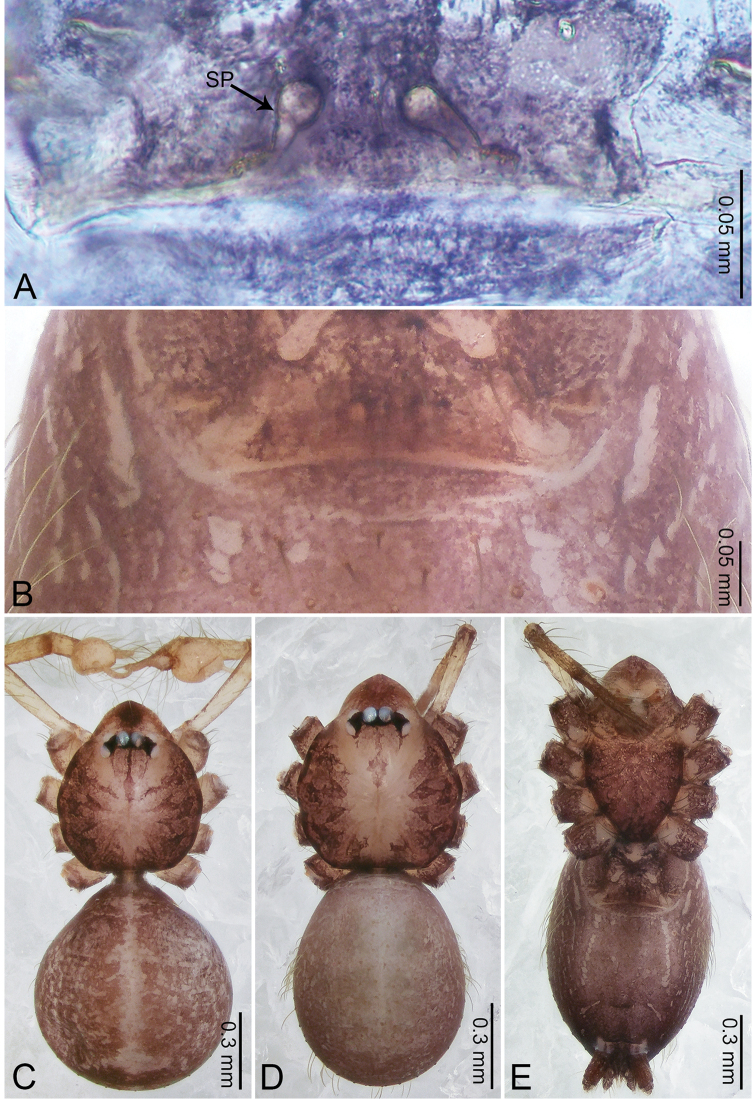
*Merizocera wui* sp. nov., holotype male and paratype female. **A** Endogyne, dorsal view **B** female epigastric area, ventral view **C** male habitus, dorsal view **D** female habitus, dorsal view **E** female habitus, ventral view. Abbreviation: SP = spermatheca.

Males resemble *M. wui* sp. nov. but can be distinguished by a broad embolus (Fig. 45B) (vs. narrow and thin embolus (Fig. 47B)), absence of a conductor (vs. presence of a short conductor (Fig. 47B)), a short and wide cymbium protrusion (Fig. 45D) (vs. long and thin cymbium protrusion (Fig. 47D)), and a pyriform bulb (Fig. 45B) (vs. spherical bulb (Fig. 47B)). Females can be distinguished by a pair of upright tubular spermathecae (Fig. 46A) (vs. a pair of angled clavate spermathecae (Fig. 48A)).

##### Description.

**Male** (holotype). Total length 1.28; carapace 0.62 long, 0.51 wide; abdomen 0.67 long, 0.58 wide. Carapace circular, brown, with dark brown radiating marks (Fig. 46C). Fovea shallow. Thoracic region distinctly elevated medially. Clypeus and labium dark brown. Sternum brown but dark brown laterally. Abdomen ovoid, dark brown. Legs light brown; measurements: I 3.45 (0.91, 0.22, 1.01, 0.80, 0.51), II missing, III 2.52 (0.67, 0.19, 0.65, 0.58, 0.43), IV 3.62 (0.94, 0.20, 1.05, 0.84, 0.59). Palp (Fig. 45A–D): femur slender, four times longer than patella; patella not swollen; tibia not swollen; cymbium with distal protrusion, half as long as femur; bulb pale yellow, pyriform with embolus merging distally; embolus clavate with blunt tip, similar in length to and approx. thrice narrower than tegular; conductor absent.

**Female** (paratype). Similar to male in colouration and general features but slightly larger (Fig. 46D, E). Measurements: total length 1.34; carapace 0.63 long, 0.55 wide; abdomen 0.71 long, 0.57 wide. Leg measurements: I 3.20 (0.81, 0.20, 0.95, 0.71, 0.53), II 2.78 (0.71, 0.22, 0.77, 0.60, 0.48), III 2.42 (0.63, 0.18, 0.63, 0.57, 0.41), IV 3.45 (0.87, 0.20, 1.04, 0.78, 0.56). Epigastric area (Fig. 46B): dark brown semi-circular patch. Endogyne (Fig. 46A) with a pair of anteriorly directed tubular spermathecae, ratio of the width of a spermatheca to the interdistance of spermathecae 1:5.

##### Distribution.

Known only from the type locality (China; Fig. 53).

#### Merizocera wui

Taxon classificationAnimaliaAraneaePsilodercidae

Li sp. nov.

00F0E486-B6DF-5BCF-87B6-282877966BC0

http://zoobank.org/E17036ED-D627-4032-AA5A-A8959CCCC170

Figures 47 , 48 , 53

##### Type material.

***Holotype***: male (IZCAS), Roadside between Wasadum and Ziradum (27°32.31'N, 97°7.54'E, elevation 978 m), Putao, **Kachin State**, **Myanmar**, 12 December 2016, J. Wu leg. ***Paratypes***: 1 male and 2 females (IZCAS), same data as holotype.

##### Etymology.

The specific name is a patronym in honour of the collector Jianglang Wu; noun (name) in genitive case.

##### Diagnosis.

Diagnosis features of males and females are discussed in *M. wenshan* sp. nov. Males with a spherical bulb, thin narrow embolus (Fig. 47B), and a narrow cymbial protrusion (Fig. 47D). Females with angled clavate spermathecae (Fig. 48A).

##### Description.

**Male** (holotype). Total length 1.53; carapace 0.67 long, 0.57 wide; abdomen 0.84 long, 0.79 wide. Carapace circular, brownish, with dark brown radiating marks (Fig. 48C). Fovea shallow. Thoracic region distinctly elevated medially. Clypeus, labium and Sternum dark brown. Abdomen ovoid, dark brown but dark grey dorso-medially. Legs light brown; measurements: I missing, II 4.34 (1.18, 0.22, 1.27, 1.10, 0.57), III 3.37 (0.88, 0.20, 0.95, 0.86, 0.48), IV 4.79 (1.19, 0.23, 1.45, 1.21, 0.71). Palp (Fig. 47A–D): femur slender, five times longer than patella; patella not swollen; tibia not swollen, half as long as femur; cymbium with distal protrusion, length ratio of dorsal elongation and cymbium 0.92; bulb spherical, with embolus and conductor arising distally; embolus slender and upright, twice longer than tegular; conductor needle-like, adjacent and basally connected to embolus.

**Female** (paratype). Similar to male in colouration and general features but slightly larger (Fig. 48D, E). Measurements: total length 1.42; carapace 0.70 long, 0.58 wide; abdomen 0.75 long, 0.60 wide. Leg measurements: I 4.11 (1.06, 0.21, 1.25, 0.99, 0.60), II missing, III 3.40 (0.89, 0.21, 0.97, 0.82, 0.51), IV 4.16 (1.06, 0.22, 1.24, 0.99, 0.65). Epigastric area (Fig. 48B): dark brown oval patch, medially with a pale brown slit. Endogyne (Fig. 48A) with a pair of clavate spermathecae, slightly angled toward each other, ratio of the width of a spermatheca to the interdistance of spermathecae 1:5.

##### Distribution.

Known only from the type locality (Myanmar; Fig. 53).

#### Merizocera yala

Taxon classificationAnimaliaAraneaePsilodercidae

Li sp. nov.

927958E4-A2A2-5D25-ADEA-8FE761B5DB28

http://zoobank.org/43BC8259-FDFE-471B-B90D-294820187195

Figures 49 , 54

##### Type material.

***Holotype***: female (IZCAS), near Suea Cave (6°31.36'N, 101°13.87'E, elevation 43 m), Mueang District, **Yala**, **Thailand**, 20 October 2015, P. Wongprom leg.

##### Etymology.

The specific name refers to the type locality; noun in apposition.

##### Diagnosis.

**Figure 49.**
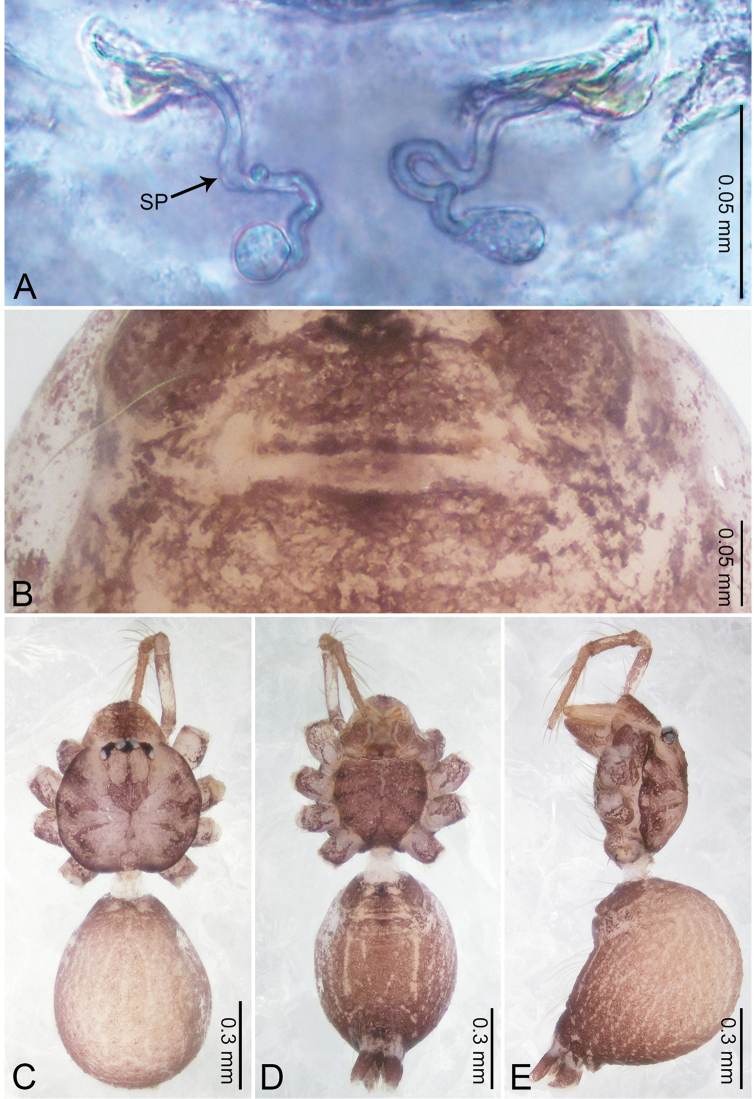
*Merizocera yala* sp. nov., holotype female. **A** Endogyne, dorsal view **B** female epigastric area, ventral view **C** female habitus, dorsal view **D** female habitus, ventral view **E** female habitus, lateral view. Abbreviation: SP = spermatheca.

Diagnosis features of females are discussed in *M. uva* sp. nov. Females with stalked spermathecae bearing globose ends (Fig. 49A).

##### Description.

**Female** (holotype). Total length 1.33; carapace 0.59 long, 0.50 wide; abdomen 0.69 long, 0.54 wide. Carapace circular, brown, with dark brown radiating marks (Fig. 49C). Fovea shallow. Thoracic region distinctly elevated medially. Clypeus and labium dark brown. Sternum dark brown, with distinct dark radiating lines. Abdomen ovoid, brownish but dark brown posteriorly and ventrally (Fig. 49B). Legs light brown; measurements: I, II, and IV missing, III 3.54 (0.97, 0.19, 1.01, 0.86, 0.51). Epigastric area (Fig. 49B): pale brown semi-circular patch, medially with dark brown horizontal slit. Endogyne (Fig. 49A) with a pair of twisted stalked spermathecae.

**Male.** Unknown.

##### Distribution.

Known only from the type locality (Thailand; Fig. 54).

#### Merizocera yuxi

Taxon classificationAnimaliaAraneaePsilodercidae

Li sp. nov.

436B4A41-38C6-546B-8A40-7382A4E6B0A2

http://zoobank.org/31847E90-FA82-42CB-A238-B99637E64341

**Figure 50.**
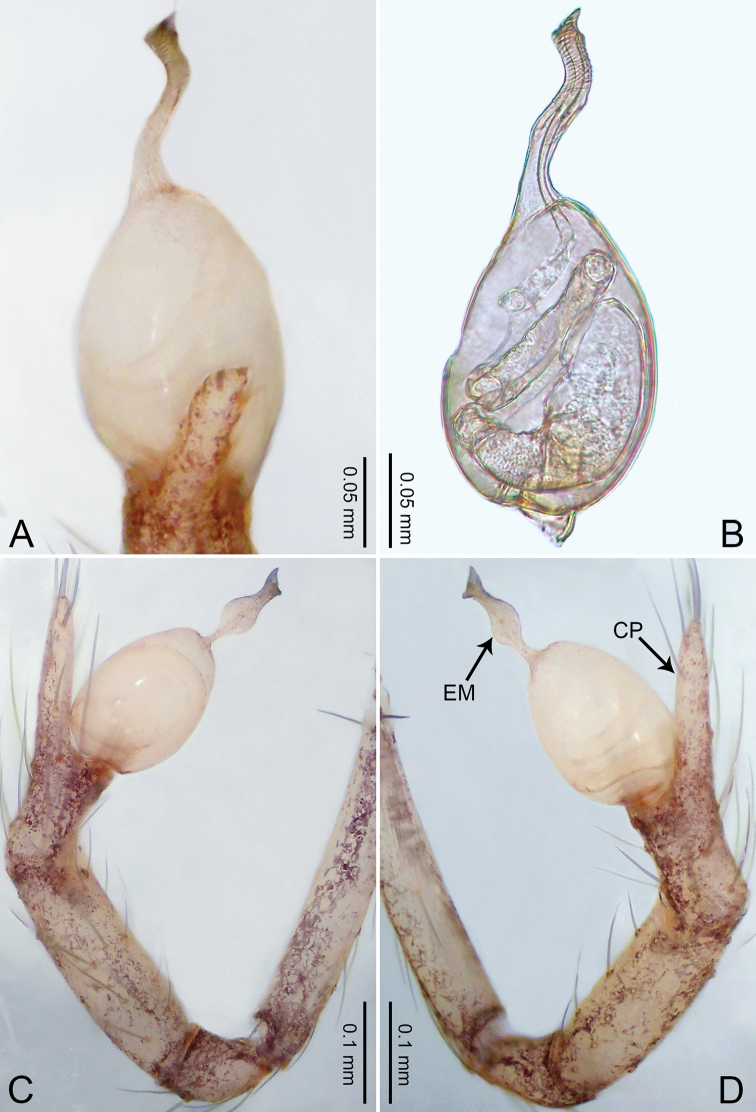
*Merizocera yuxi* sp. nov., holotype male. **A** Bulb, dorsal view **B** bulb, dorsal view **C** palp, prolateral view **D** palp, retrolateral view. Abbreviations: CP = cymbial protrusion, EM = embolus.

**Figure 51.**
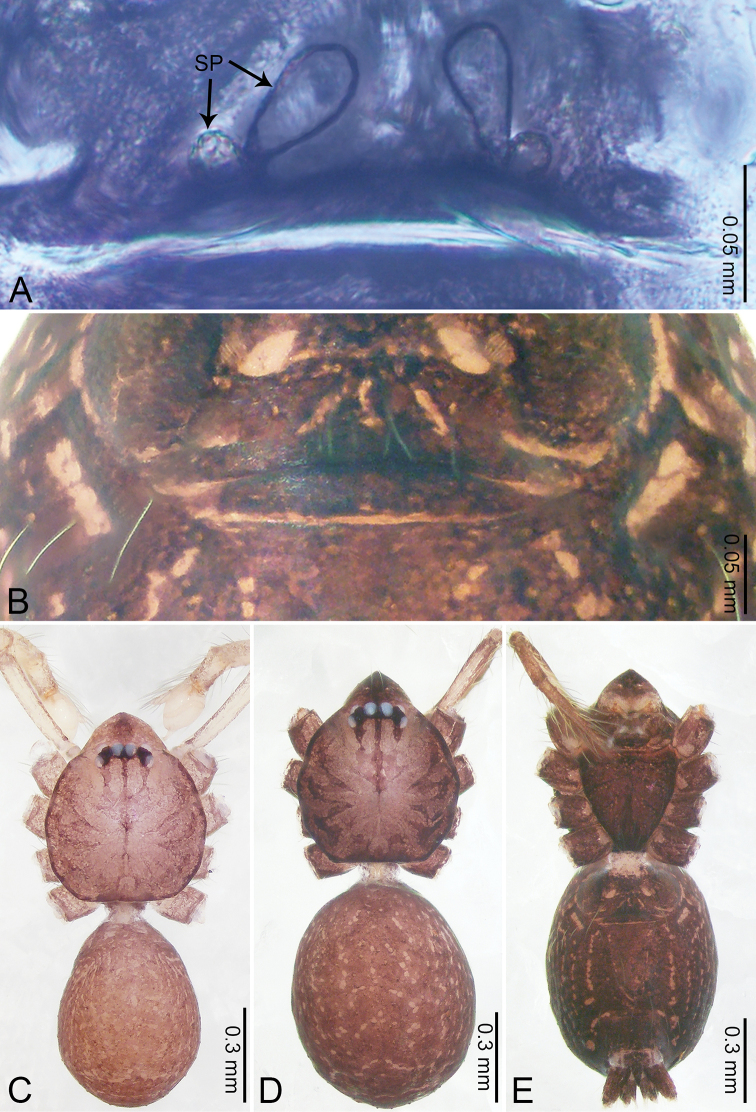
*Merizocera yuxi* sp. nov., holotype male and paratype female. **A** Endogyne, dorsal view **B** female epigastric area, ventral view **C** male habitus, dorsal view **D** female habitus, dorsal view **E** female habitus, ventral view. Abbreviation: SP = spermatheca.

Figures 50 , 51 , 53

Merizocera sp. 249: Li and Li 2018 (molecular data).

##### Type material.

***Holotype***: male (IZCAS), Guzhouyelin (24°6.63'N, 101°51.00'E, elevation 1987 m), Xinhua Town, Xinping County, Yuxi, **Yunnan**, **China**, 2 June 2015, Z. Chen and Y. Li leg. ***Paratypes***: 1 male and 2 females (IZCAS), same data as holotype.

##### Etymology.

The specific name refers to the type locality; noun in apposition.

##### Diagnosis.

Diagnosis features of males and females are discussed in *M. betong* sp. nov. Males with swollen pyriform bulb, and embolus with a crinkly and flattened tip (Fig. 50B). Females with two pairs of tubular spermathecae (Fig. 51A).

##### Description.

**Male** (holotype). Total length 1.25; carapace 0.61 long, 0.51 wide; abdomen 0.62 long, 0.48 wide. Carapace circular, brown, with dark brown radiating marks (Fig. 51C). Fovea shallow. Thoracic region distinctly elevated medially. Clypeus and labium dark brown. Sternum dark brown, with dark radiating lines. Abdomen ovoid, dark brown. Legs light brown; measurements: I, III, and IV missing, II 3.11 (0.83, 0.20, 0.89, 0.70, 0.49). Palp (Fig. 50A–D): femur slender, four times longer than patella; patella not swollen; tibia not swollen; cymbium with distal protrusion, 1/3 length of femur, length ratio of dorsal elongation and cymbium 1.13; bulb pyriform, with embolus arising distally; embolus crinkly with flattened tip, with distinct swollen section medially, similar length as the tegular; conductor absent.

**Female** (paratype). Similar to male in colouration and general features but slightly larger (Fig. 51D, E). Measurements: total length 1.45; carapace 0.66 long, 0.54 wide; abdomen 0.79 long, 0.63 wide. Leg measurements: I 3.46 (0.90, 0.22, 1.01, 0.79, 0.54), II 2.99 (0.79, 0.20, 0.84, 0.67, 0.49), III missing, IV 3.69 (0.91, 0.21, 1.10, 0.84, 0.63). Epigastric area (Fig. 51B): dark brown, nearly trapezoidal patch. Endogyne (Fig. 51A) with two pairs of spermathecae, lateral pairs globose, median pairs clavate, lateral pairs attached basally with the median pairs.

**Figure 52.**
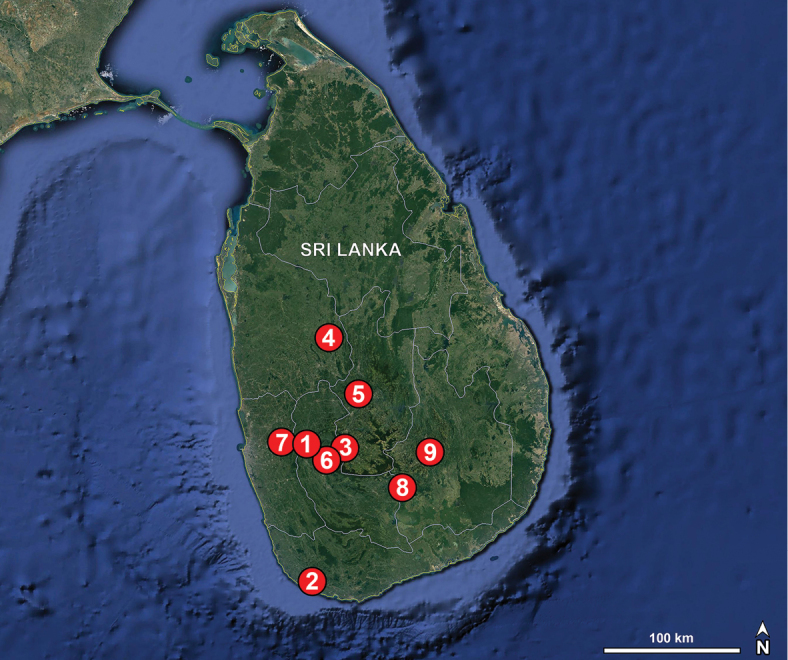
Distribution records of the new species of *Merizocera* from Sri Lanka. **1***M. colombo* sp. nov. **2***M. galle* sp. nov. **3***M. kandy* sp. nov. **4***M. kurunegala* sp. nov. **5***M. peraderiya* sp. nov. **6***M. ratnapura* sp. nov. **7***M. salawa* sp. nov. **8***M. thenna* sp. nov. **9***M. uva* sp. nov.

##### Distribution.

**Figure 53.**
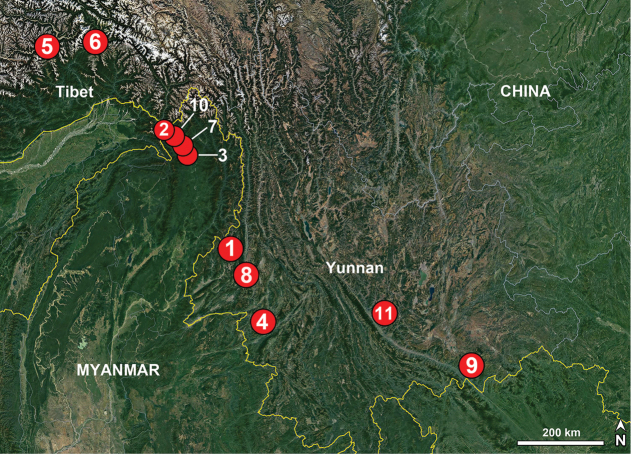
Distribution records of the new species of *Merizocera* from China and Myanmar. **1***M. baoshan* sp. nov. **2***M. hponkanrazi* sp. nov. **3***M. kachin* sp. nov. **4***M. lincang* sp. nov. **5***M. mainling* sp. nov. **6***M. nyingchi* sp. nov. **7***M. putao* sp. nov. **8***M. tengchong* sp. nov. **9***M. wenshan* sp. nov. **10***M. wui* sp. nov. **11***M. yuxi* sp. nov.

Known only from the type locality (China; Fig. 53).

**Figure 54.**
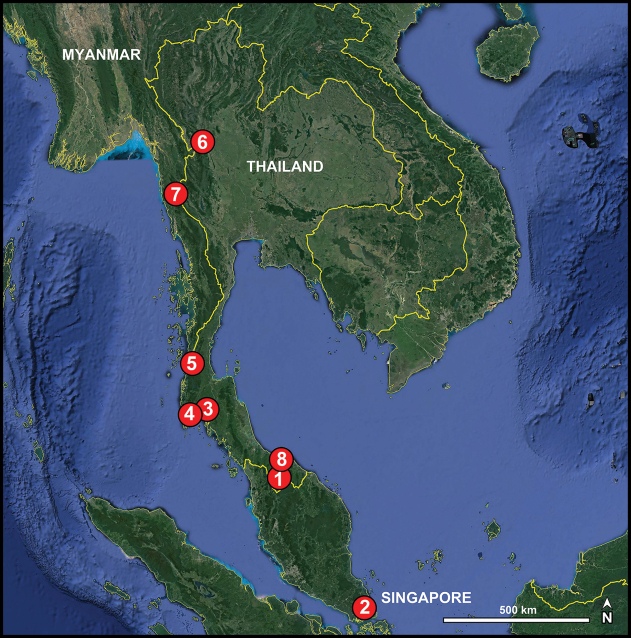
Distribution records of the new species of *Merizocera* from Thailand, Myanmar and Singapore. **1***M. betong* sp. nov. **2***M. mandai* sp. nov. **3***M. krabi* sp. nov. **4***M. phuket* sp. nov. **5***M. ranong* sp. nov. **6***M. tak* sp. nov. **7***M. tanintharyi* sp. nov. **8***M. yala* sp. nov.

## Discussion

Males of *Merizocera* can be distinguished from *Psiloderces* by the following characters: In *Merizocera* the cymbium and bulb are of similar lengths, or rarely the bulb is longer than the cymbium. In contrast, the cymbium is generally longer than the bulb in *Psiloderces*. *Merizocera* can be divided into six morphological groups of species based on male palps. *Merizocera betong* sp. nov., *M. ranong* sp. nov., *M. wenshan* sp. nov., *M. wui* sp. nov., and *M. yuxi* sp. nov. form a group of species with a rounded or almost pyriform bulb and a rather simple embolus (not overly elongated and curved); *M. thenna* sp. nov., *M. salawa* sp. nov., *M. kurunegala* sp. nov., *M. peraderiya* sp. nov., *M. colombo* sp. nov., *M. kandy* sp. nov., *M. oryzae*, and *M. picturata* form a group of species with a furcate conductor and distally arising embolus; *M. tengchong* sp. nov., *M. putao* sp. nov., *M. kachin* sp. nov., and *M. baoshan* sp. nov. form a group of species with a distinctly elongated, slender bulb, distally with bent embolus or stalked apophysis; *M. mandai* sp. nov. and *M. crinita* form a group of species with similarly slender, distally arising embolus and conductor; *M. tanintharyi* sp. nov., *M. mainling* sp. nov., *M. cruciata*, and *M. brincki* form a group of species which have a short or inconspicuous cymbial protrusion, and distally arising embolus and conductor; and *M. krabi* sp. nov., *M. ratnapura* sp. nov., *M. phuket* sp. nov., *M. hponkanrazi* sp. nov., and *M. galle* sp. nov. form a group of species with a distinctly elongated and curved embolus. It is not feasible to co-relate the species groups based on female characters.

## Supplementary Material

XML Treatment for Merizocera

XML Treatment for Merizocera baoshan

XML Treatment for Merizocera betong

XML Treatment for Merizocera colombo

XML Treatment for Merizocera galle

XML Treatment for Merizocera hponkanrazi

XML Treatment for Merizocera kachin

XML Treatment for Merizocera kandy

XML Treatment for Merizocera mandai

XML Treatment for Merizocera krabi

XML Treatment for Merizocera kurunegala

XML Treatment for Merizocera lincang

XML Treatment for Merizocera mainling

XML Treatment for Merizocera nyingchi

XML Treatment for Merizocera peraderiya

XML Treatment for Merizocera phuket

XML Treatment for Merizocera putao

XML Treatment for Merizocera ranong

XML Treatment for Merizocera ratnapura

XML Treatment for Merizocera salawa

XML Treatment for Merizocera tak

XML Treatment for Merizocera tanintharyi

XML Treatment for Merizocera tengchong

XML Treatment for Merizocera thenna

XML Treatment for Merizocera uva

XML Treatment for Merizocera wenshan

XML Treatment for Merizocera wui

XML Treatment for Merizocera yala

XML Treatment for Merizocera yuxi
